# MiRComb: An R Package to Analyse miRNA-mRNA Interactions. Examples across Five Digestive Cancers

**DOI:** 10.1371/journal.pone.0151127

**Published:** 2016-03-11

**Authors:** Maria Vila-Casadesús, Meritxell Gironella, Juan José Lozano

**Affiliations:** 1 Bioinformatics Platform, Centro de Investigación Biomédica en Red de Enfermedades Hepáticas y Digestivas (CIBEREHD), Barcelona, Catalonia, Spain; 2 Gastrointestinal and Pancreatic Oncology Group, Hospital Clínic of Barcelona, Centro de Investigación Biomédica en Red de Enfermedades Hepáticas y Digestivas (CIBEREHD), Institut d'Investigacions Biomèdiques August Pi i Sunyer (IDIBAPS), Barcelona, Catalonia, Spain; Roswell Park Cancer Institute, UNITED STATES

## Abstract

MicroRNAs (miRNAs) are small RNAs that regulate the expression of target mRNAs by specific binding on the mRNA 3'UTR and promoting mRNA degradation in the majority of cases. It is often of interest to know the specific targets of a miRNA in order to study them in a particular disease context. In that sense, some databases have been designed to predict potential miRNA-mRNA interactions based on hybridization sequences. However, one of the main limitations is that these databases have too many false positives and do not take into account disease-specific interactions. We have developed an R package (miRComb) able to combine miRNA and mRNA expression data with hybridization information, in order to find potential miRNA-mRNA targets that are more reliable to occur in a specific physiological or disease context. This article summarizes the pipeline and the main outputs of this package by using as example TCGA data from five gastrointestinal cancers (colon cancer, rectal cancer, liver cancer, stomach cancer and esophageal cancer). The obtained results can be used to develop a huge number of testable hypotheses by other authors. Globally, we show that the miRComb package is a useful tool to deal with miRNA and mRNA expression data, that helps to filter the high amount of miRNA-mRNA interactions obtained from the pre-existing miRNA target prediction databases and it presents the results in a standardised way (pdf report). Moreover, an integrative analysis of the miRComb miRNA-mRNA interactions from the five digestive cancers is presented. Therefore, miRComb is a very useful tool to start understanding miRNA gene regulation in a specific context. The package can be downloaded in http://mircomb.sourceforge.net.

## Introduction

MicroRNAs (miRNAs) are non-coding, single-stranded RNAs of 18–25 nucleotides and constitute a novel class of gene regulators that are found in both plants and animals. They negatively regulate their targets (messenger RNAs -mRNAs-) in one of two ways depending on the degree of complementarity between the miRNA and the target. One way of action (that accounts for around 80% of the cases) is promoting mRNA degradation [1], the other one is inhibiting mRNA translation.

Previous authors have used paired miRNA and mRNA data for predicting miRNA targets in specific diseases. They base their analysis on correlating miRNA and mRNA expression, and intersecting it with known databases [2,3]. However, although these studies are useful, there is no software available to reproduce the results. R [4] is a software environment for statistical computing and graphics. It has been broadly used in the scientific community due to the fact that works with any platform, is free, allows building your own packages and functions and share it with other scientists, it is well documented and kept updated. Bioconductor [5] is an R package repository focused on packages aimed to analyse biological data. There are some R/Bioconductor packages that are able to make miRNA-mRNA correlations, intersect with known databases and analyse networks, among other functionalities, such as *RmiR*, *CORNA*, *miRNApath*, *microRNA*, *MultiMiR* [6,7]. However, none of these methods allows performing an entire complete analysis in a straightforward way. Our aim was to design an R package, called miRComb, able to combine miRNA and mRNA expression data (from any format) with hybridization information, in order to find potential miRNA-mRNA targets that are likely to occur in a specific physiological or disease context. This generates a list of results that can be the basis for developing multiple hypotheses to be experimentally tested in a wet lab. Another added value is to present the results of the analysis in a standarized way with a pdf report.

We have used as examples publicly available data from The Cancer Genome Atlas (TCGA) [8] for different digestive cancers. The results highlight potential miRNA-mRNA interactomes of five digestive cancers and offer an unbiased view of miRComb functions. As far as we know, there is still no global analysis of this kind in gastrointestinal cancers.

## Materials and Methods

We have used TCGA data from 1645 samples among 5 different digestive cancers (colon cancer, rectal cancer, liver cancer, stomach cancer and esophageal cancer) that had simultaneously miRNA-seq and RNA-seq data. All data have been processed with the same procedure.

As the starting point of our package we used three broadly accepted assumptions:

- MiRNA negatively regulate expression of their mRNA targets.
- MiRNA/mRNA interactions, as they are based on RNA hybridization, can be predicted with bionformatic approaches.
- MiRNAs and mRNAs that play a role in a specific disease are deregulated in that disease.

**Fig 1:**
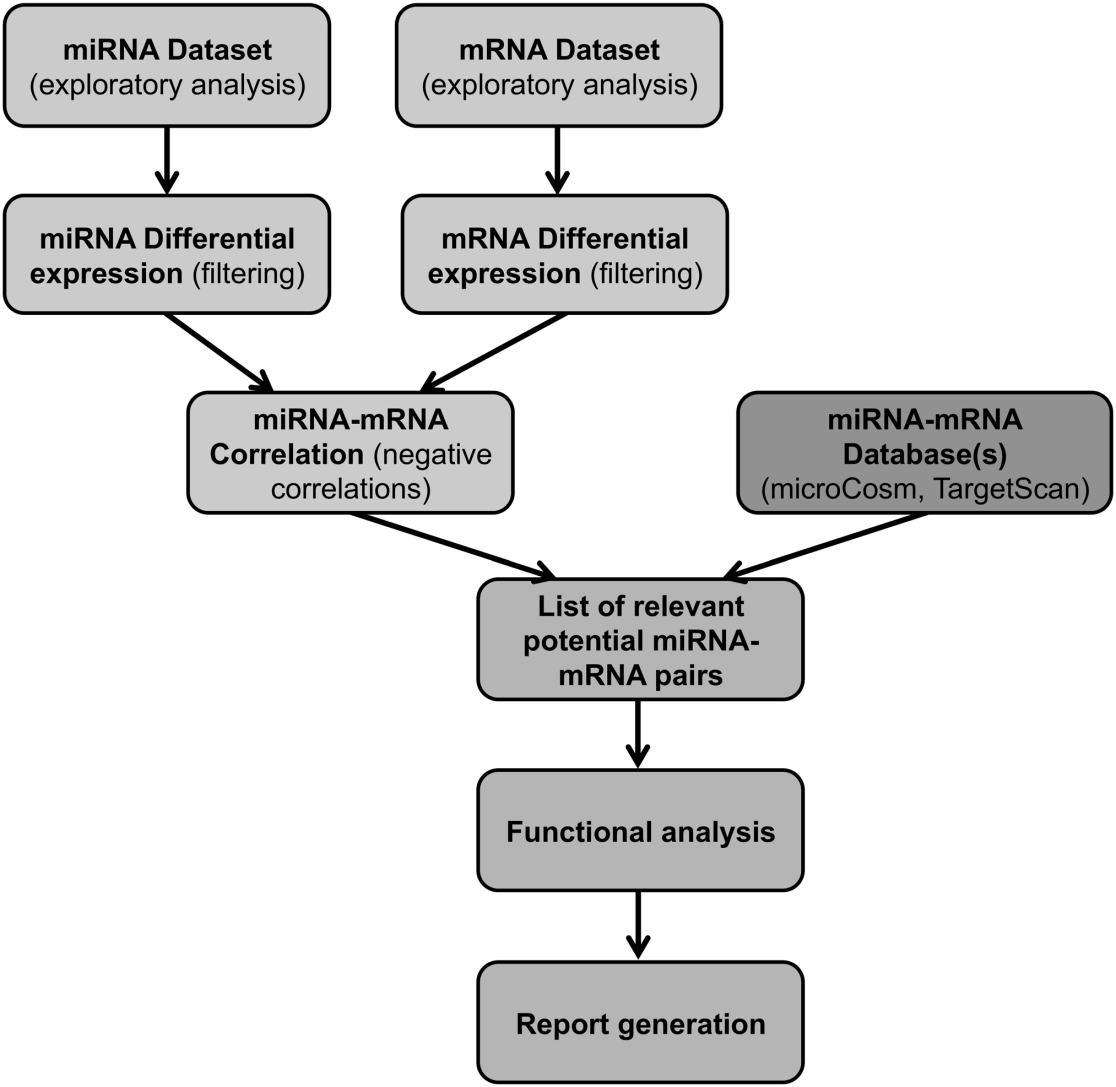
Flow diagram showing the main steps of an analysis using the *miRComb* package.

Fig 1 shows the outline of the procedure used. Raw data is processed with the aim of finding relevant miRNA-mRNA interactions in a specific biological context in order to be able to interpret them. The package is written in R and includes some code of C++ in order to speed up some computations. LaTeX [9] and Sweave [10] packages are used to generate the final pdf report. MiRComb is available at http://mircomb.sourceforge.net/.

MiRNA and mRNA expression data can come from different sources (microarrays, NGS, qRT-PCR…). The package assumes that miRNA and mRNA data are properly normalized. In the case of qRT-PCR data we suggest using -dCt units (or Ct units), for microarrays we suggest using log2 (normalized) intensity, and for NGS we suggest using log2 (normalized) counts.

### Data sources

Data was downloaded from TCGA data portal (https://tcga-data.nci.nih.gov/tcga/dataAccessMatrix.htm). We selected the following cancers to study: Colon adenocarcinoma (COAD); Esophageal carcinoma (ESCA); Liver hepatocellular carcinoma (LIHC); Rectum adenocarcinoma (READ); Stomach adenocarcinoma (STAD).

We selected only those samples that had paired miRNA and mRNA information and came from centers (properly identified with their corresponding Tissue Source Sites–TSS- codes) that collected more than one sample. Primary solid Tumor and Solid tissue Normal were used. MiRNAs with no id (on mirbase17) or median expression < 10 raw counts were removed. MRNAs with no gene id or median expression < 10 raw counts were removed. Voom transformation [11] and quantile normalization were applied, and then batch correction with ComBat [12] according to TSS centers was applied.

### Differential expression analysis

Differential expression between cases and controls was computed with limma-trend procedure. The package also implements T-test, Wilcoxon Test, LIMMA, LIMMA-trend [11], and RankProd [13] for testing differences between both groups. However, other methods for differential expression can be used and the results can be also imported to *miRComb* (the features needed are miRNA or mRNA, logratio, mean expression, *p* value and adjusted *p* value). Multiple testing procedures can be: Benjamini & Hochberg (BH), Bonferroni or others (although RankProd assumes only BH (which controls False Discovery Rate (FDR)).

For parametric approaches, the hypothesis is if the mean expression of the Control group samples (H) is different from the mean expression of the Cancer-related group samples. In the case of non-parametric approaches–Wilcoxon test and RankProd-, the median (instead of the mean) is tested.

$$\{\begin{array}{c} H_{0}:\;\mu_{Cancer}\;=\;\mu_{Healthy}\\ H_{1}:\;\mu_{Cancer}\neq \;\mu_{Healthy} \end{array}$$

### Correlation analysis

We computed Pearson correlation coefficients for all miRNA-mRNA couples available in each cancer. The package also supports Spearman and Kendall correlation (Kendall only for small datasets). Pearson correlation is suitable if both miRNA and mRNA data come from the same platform analysis (both microarrays or log2-normalised count data, for example), and a lineal relation between miRNA and mRNA can be assumed. If both analysis platforms are different or the hypotheses of a lineal relation cannot be assumed, then Spearman (or Kendall) correlation are desirable. If there is a negative relation between miRNA (X_1_, …,X_n_) and mRNA (Y_1_, …,Y_n_) the correlation coefficient would be negative, so:

$$\{\begin{array}{c} H_{0}:\;\rho_{\lambda }\geq 0\\ H_{1}:\;\rho_{\lambda }<0 \end{array}$$

Where ∈ {*Pearson*, *Kendall*, *Spearman*}. Then, multiple testing correction (Bonferroni and BH are available, among other options) is applied in order to control the false positives that could arise.

### Intersection with miRNA target prediction databases

Next step was to match the significant correlations with target information. The choice of a database is a tricky issue. Several databases are aimed to computationally predict miRNA targets [14]. They mainly take into account at least one of these parameters: seed complementarity, miRNA-mRNA complex stability (thermodynamics) and inter-species site conservation. Several databases start integrating miRNA-mRNA correlation as a predictive value, but it has been done in few datasets (GenMiR++) [15].

We selected MicroCosm [16,17] and TargetScan [18]. MicroCosm comprises 690 different miRNA and 22107 different targets, with a total of 563179 interactions described. MicroCosm computes the targets with miRanda algorithm, needing perfect complementarity at the 5'; then excludes non-stable conformations by using the Vienna RNA folding approach [19] and requires site conservation accross several species. On the other hand, TargetScan [18] is probably one of the most updated ones. It contains information for 1537 different miRNAs and 15031 targets, with a total of 520354 interactions described. It is based on seed complementarity and differentiates among conserved and non-conserved sites. In order to make it more comparable to microCosm, and more reasonable, we selected only conserved sites. The package allows to use one or both databases (and also use custom databases, if desired), and fix a minimum number of appearances on the database. The final conditions that define a miRNA-mRNA interaction are:

$$\{\begin{array}{c} H_{0}:\;\rho_{\lambda }\geq 0\;\;\;or\;\;\;!is.target\\ H_{1}:\;\rho_{\lambda }<0\;\;\;and\;\;\;is.target \end{array}$$

### Functional analysis

Although the main aim of the package is to generate a list of potential miRNA-mRNA pairs, *miRComb* also implements some functions that may help to data interpretation. Among other functions specified in the following sections, tables and barplots with the number of targets or number of miRNAs can be obtained. The package plots a network with the desired miRNA-mRNA interactions (nodes representing miRNAs and mRNAs, and arrows marking the interactions). Colours are carefully selected to help interpretation: miRNAs are represented as squares, mRNAs as circles. Furthermore, the colour of the node reflects the FoldChange direction of the node (red: upregulated, green: downregulated). A score is computed with the aim to reflect the impact of the miRNA on the cancer (higher score means that both miRNA and mRNA are highly deregulated in that disease).

$$score\;=\;-2\left(logratio_{miRNA}\cdot logratio_{mRNA}\right)$$

Arrows are also informative: the colour represents the *score* of the interaction (red means opposite and strong opposed FC between the miRNA and the mRNA, while green would represent strong concordant FC between the miRNA and the mRNA), and the width represents the number of databases where the target has been found (more databases: wider arrow). The network can also be easily exported to cytoscape in “sif” format [20], as well node and edge attributes.

*GOstats* package [21] was used to compute if any function is associated with the targets of a specific miRNA or a set of miRNAs. This helps to predict the function of a miRNA or a set of miRNAs if the number of targets is big enough. *RamiGO* R package [22] can also be used to plot the significant GO terms and their relations.

*Circlize* package [23] is used to make a circos plot of the selected miRNA-mRNA pairs. Starting from the border of the plot, the position of the mRNAs is represented on a first track, then a second track represents miRNAs position, and finally a last track (with links) shows miRNA-mRNA pairs. This would help to identify if some miRNA targets are more specifically located in one chromosome or region.

### Report generation

One of the aims of the project is to present a standarized way to present the results. At the end of the analysis is possible to generate a pdf report which includes all the mentioned sections.

## Results and Discussion

### MiRComb analysis of miRNA-mRNA interactions of 5 different digestive cancers

**Fig 2:**
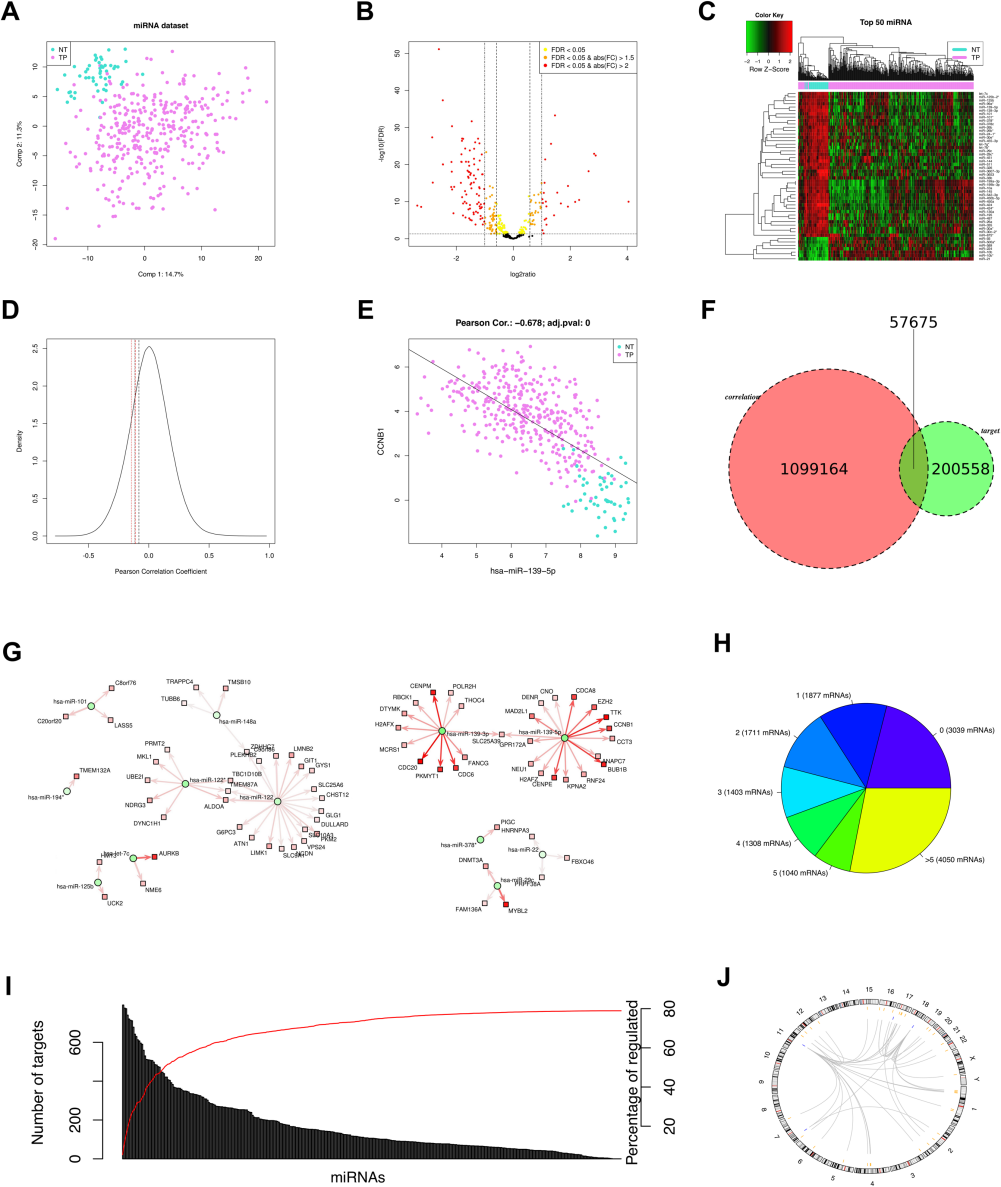
Main findings of the LIHC report. A) Principal Components Analysis (PCA) (based on correlation matrix) of miRNA samples. B) Volcano plot showing the miRNAs according to its logratio between cancer and control. C) Heatmap of the top 50 most deregulated miRNAs according to its FDR. D) Density plot of the Pearson Correlation Coefficients of all possible miRNA-mRNA interactions. Lines show different cutoff: p-value < 0.05, p-value < 0.01, FDR < 0.05 and FDR < 0.01. E) Correlation of miR-139-5p and CCNB1 as an example. F) Venn diagram showing the total number of sigifnicant correlations (FDR < 0.05), the total number of predicted interactions in at least one database (TargetScan or microcosm), and the intersection of both. G) Network of selected interactions. Each miRNA-mRNA interaction is negatively correlated (FDR < 10–33) and predicted at least in one database (Targetscan or MicroCosm). Circles represent miRNAs and squares mRNAs; red fill means upregulated miRNA/mRNA, while green fill means downregulated miRNA/mRNA; lines indicate the miRNA-mRNA pairs; red line means positive score and green line means negative score; arrow width is proportional to the number of appearances on the databases (TargetScan or MicroCosm). H) Pie chart showing the number of mRNAs regulated by 0, 1, 2, 3, 4, 5, and >5 miRNAs. I) Barplot showing the number of targets per miRNA and the percentage of mRNAs that are cumulatively regulated by the miRNAs. J) Circos plot of the top 45 miRNA-mRNA interactions sorted by FDR, a line means a miRNA-mRNA pair. Blue lines are the position of the miRNAs and orange lines are the position of the mRNAs.

Five miRComb reports for COAD, READ, ESCA, STAD and LIHC were generated and the corresponding pdf files can be found in S1–S5 Files, respectively. As an example, Fig 2 shows the main figures from the LIHC report.

#### Summary of datasets composition

Table 1 shows the number of samples available for each cancer and the total number of significant correlations. COAD, LIHC and STAD cancer had more than 400 samples available for analysis, while ESCA cancer and READ cancer data sets had 191 and 160 samples, respectively. Moreover, the ratio between cases and controls is also a term to take into account. While ESCA, LIHC and STAD disposed of a “reasonable” amount of controls (approximately 1:13 for ESCA, 1:7 for LIHC and 1:10 for STAD), in COAD and LIHC we disposed of only 8 and 3 controls respectively (a ratio of aproximately 1:50). The number of available samples influences the number of correlations with FDR < 0.05 found: the more samples we have, the higher is the power for detecting correlations different from 0. The number of significant correlations found are higher than 15% (even after FDR correction) in the data sets with more than 400 samples (STAD, LIHC, COAD), while this percentage does not reach 10% in the cases of READ and ESCA (less than 200 samples available). In short, it seems that a dataset with a bigger sample size and a balanced design should provide a greater number of correlations that one that is smaller and not balanced.

**Table 1:** Summary of the main miRComb computations of the five digestive cancer data sets analysed.

	COAD	ESCA	LIHC	READ	STAD
**Number of samples (cases, controls)**	444 (436, 8)	191 (178, 13)	407 (357, 50)	160 (157, 3)	443 (406, 37)
**Number of expressed miRNAs**	325	338	343	325	330
**Number of expressed mRNAs**	14860	18807	14428	14973	18565
**Total correlations computed**	4829500	6356766	4948804	4866225	6126450
**Significant correlations (%respect total correlations computed)**	823121 (17.04%)	568914 (8.95%)	1156839 (24.38%)	423296 (8.70%)	1390596 (22.70%)
**Significant correlations + targets**	47134	30061	57675	24941	71464

Although 20.531 mRNAs and 1025 miRNAs were sequenced, only around 32–34% of the miRNAs were considered expressed (median counts > 10 across all samples) in each cancer data set. In contrast, 70–90% of the mRNAs were detected with a median > 10 counts. In general, PCA analysis (pages 1 and 2 of the reports made by mkReport function, for example S1–S5 Files) of samples revealed a really slight control clusterization (except for miRNA dataset in COAD, READ and in both data sets in LIHC). Overall, this leads to the idea that the main drawback of the data set is the lack of a reasonable number controls, reinforcing the thoughts that differential expression between both groups can be computed and used as informative item, but not as a filtering step (that could lead to failures in the sense of false negatives).

Volcano plots (pages 3 and 4 of the reports or Fig 2B) highlight in red the selected miRNAs and mRNAs. Heatmaps are also plotted (pages 3 and 4 of the reports). Heatmap of LIHC as an example is also shown in Fig 2C.

#### Analysis of miRNA-mRNA interactions

Page 5 of the pdf reports shows the summary of the computed correlations. The next step is to intersect the significant correlations with predicted miRNA-mRNA potential interactions from Microcosm or TargetScan prediction databases (pages 6 and 7 of the reports). For the case of LIHC (Fig 2F), we observed that the predicted number of miRNA-mRNA interactions was reduced from 258233 to 57675, therefore, we could estimate that around 80% of the initial miRNA-mRNA predicted interactions from databases were false positives for this disease because they did not show a negative correlation between the specific miRNA and the specific mRNA expression *in vivo* in the tissue.

**Fig 3:**
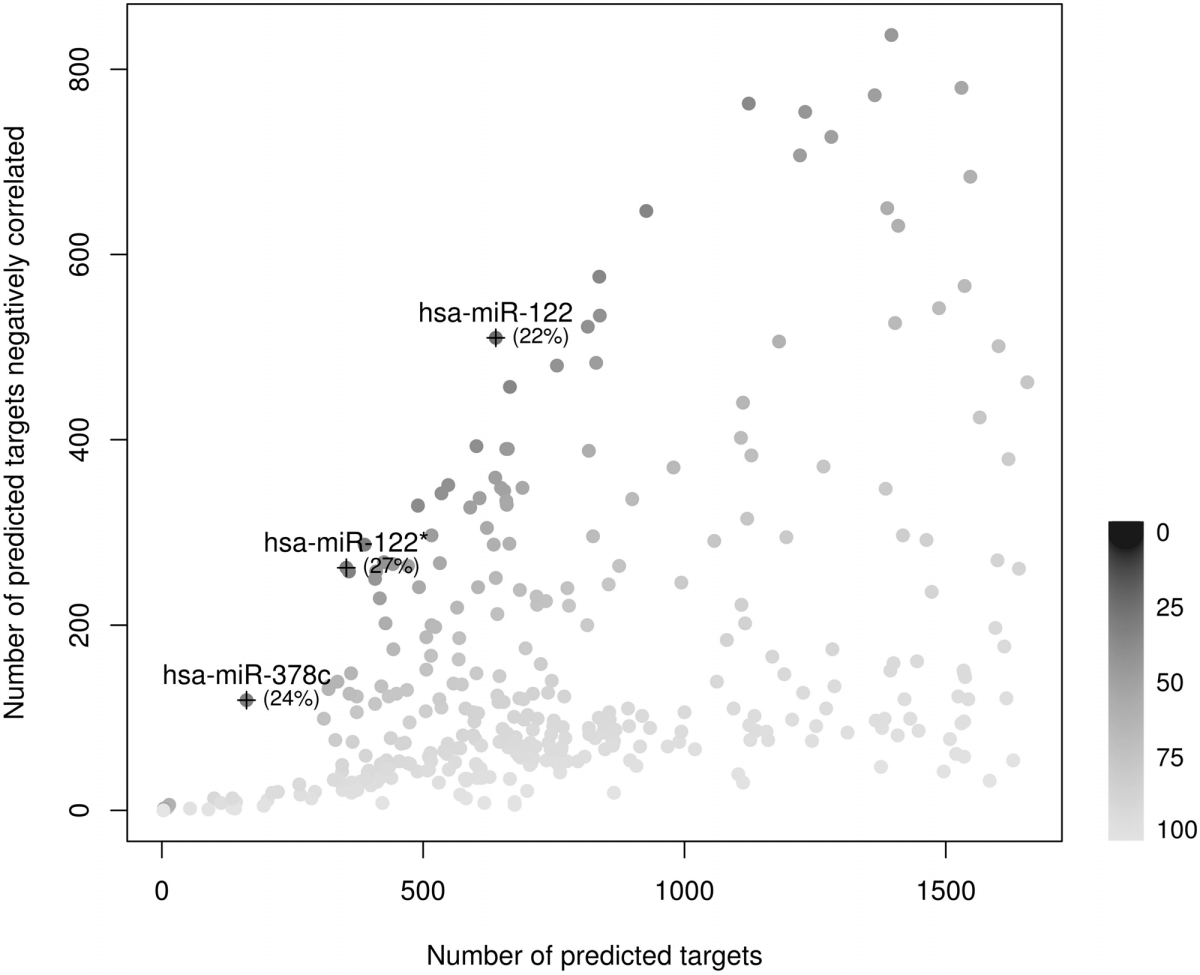
Percentage of false positive miRNA-mRNA predicted interactions in LIHC. Plot showing the ratios of negatively correlated predicted targets respect to all predicted targets according to the databases for each miRNA. The intensity of the grey color dot is related to the percentage of false postive miRNA-mRNA predicted interactions. In brackets, the exact percentages of false positivesfrom selected miRNAs (miR-122; miR-122*; miR-378c).

Furthermore, Fig 3 shows that we can also depict the proportion of false positive predicted targets of each miRNA from databases in a given situation. Concerning LIHC, the number of false postives ranges from 22% to 99%. In the case of miR-122, miR-122* or miR-378c these percentages are quite low compared to the others (22%, 27% and 24% respectively), therefore these miRNAs show a high ratio of predicted targets confirmed by miRComb. Interestingly, miR-122 is the most frequent miRNA in the adult liver, and plays a central role in liver biology and hepatocarcinoma disease [24].

Page 6 of the pdf report shows the top 15 miRNA-mRNA interactions (sorted by adjusted p-value, taking into account that they must have been predicted in at least one database) in each cancer. Page 9 of the pdf report shows the network of all the miRNA-mRNA interactions. All the interactions are plotted by default and this could result in a very dense figure difficult to interpret, as it is the case in our examples. For the case of all the interactions of LIHC (S5 File page 9) we can see two main patterns: on the left we can find mostly downregulated miRNAs in LIHC (plotted as green circles) together with their correspondant mRNA targets (plotted as red squares). On the right the roles are inverted, and the predominant miRNA-mRNA interactions shown consist on upregulated miRNAs with downregulated mRNAs. This general pattern is reproduced in all the studied cancers (S1–S5 Files). To solve this problem, we suggest to adapt in every case the number of interactions to be plotted depending on the goal of the figure. In Fig 2G we have plotted a reduced amount of interactions and we can see some of the details. For example, two targets (DNMT3A and MYBL2) of hsa-miR-29c (bottom right) are predicted by two databases, while the target FAM136A is predicted by only one database (the arrow is thiner). Moreover, regarding the targets of hsa-let7c, the AURKB is more deregulated in LIHC than the NME6, and the interactions of hsa-miR-122 or hsa-miR-122* (top left) have lower scores (lower intensity of arrow colour) than the interactions of hsa-miR-139-3p and hsa-miR-139-5p (higher intensity of arrow colour; top right).

In LIHC more than 75% of the expressed mRNAs are being targeted by at least one miRNA (Fig 2H and 2I and page 10 of the pdf report), in COAD and STAD that number is between 70% and 60%, while in READ and ESCA is less than 50%. However, we have to take into account that these percentages are partially affected by the total number of miRNA-mRNA predicted interactions: the higher number of interactions, the higher number of miRNAs per mRNA (and viceversa). For example, more than 25% of the miRNAs in LIHC are predicted to be targeted by more than five miRNAs. This percentage is lower in the other cancers, but it is still 8% in READ. It is worth to mention that this is a first approach that will require interactions to be experimentally confirmed in a wet lab. This unusual number of miRNAs targeting the same mRNA could be attributed to the fact that miRComb does not take into account competitivity between different miRNAs hybridizing to the same target.

Page 11 of the pdf report shows the first 20 miRNAs sorted by number of targets. As an example, miR-106a has 766 interactions predicted in COAD, miR-27a has 450 interactions in ESCA, miR-27b has 792 interactions in LIHC, miR-106a has 582 interactions in READ, and miR-29a has 798 interactions in STAD. Although miRNAs are expected to regulate up to hundreds of genes, these interactions should be experimentally validated in order to discard false positives or indirect relations, as mentioned above. Colours in these pages show the direction of miRNA deregulation (red: up-regulated; green: down-regulated). While in COAD, READ and ESCA the top miRNAs are in general upregulated, in LIHC and STAD they are mostly downregulated. MRNAs can also be sorted according the number of miRNAs that are targeting them (page 12 of the report) and are also coloured according to the direction of deregulation. Overall, mRNAs do not have more than 50 miRNAs regulating them. Exceptionally, in STAD there are some mRNAs with more than 60 miRNAs (eg. 74 for FOXP2). However, it is worth to take into account that the vast majority of mRNAs that are regulated by at least 1 miRNA, are simultaneously regulated by up to 4 miRNAs.

In general terms, the main direction of the top mRNAs (sorted by number of miRNA targeting them, report page 12) is the inverse of the main direction of the top miRNAs (sorted by number of targets, report page 11).

#### Functional enrichment analysis of miRNAs according to their targets

In pages 13–15 of the report, we can find the Gene Ontology (GO) and KEGG functional analysis of the results. As an example, we tested if the mRNAs that are regulated by miRNAs are enriched in any of the GO and KEGG categories. Results of this section are quite similar between all digestive cancer data sets because they include all mRNAs that are targeted by at least one miRNA and it includes more than 50% of the expressed mRNAs on average. Depending on the goal of the study different filters could be applied (differential expressed miRNAs and/or mRNAs, targets from one specific miRNA…) and, then, results would be different. In this case, BP (Biological Process) overrepresented terms include cellular process and other regulating and signalling processes. CC (Cellular Component) overrepresented terms are mostly related to intracellular-cytoplasm compartments. MF (Molecular Function) overrepresented terms are centered in protein binding and other binding (enzyme, anion binding) actions. KEGG pathways are more concise and all of them include the term “Pathways in cancer”. COAD also included prostate cancer and chronic myeloid leukemia and glioma, ESCA also small cell lung cancer, LIHC included prostate cancer, colorectal cancer, pancreatic cancer, chronic myeloid leukemia and renal cell carcinoma; READ included renal cell carcinoma, STAD also included small cell lung cancer and prostate cancer. This suggests that, as known, many cancers share similar patterns. Other pathways that are shared across the different studied data sets are: Focal adhesion, Fc-gamma R-mediated phagocytosis (COAD, ESCA, STAD), or TGF-beta signalling pathway (COAD, READ).

More targeted results can be obtained by testing for enrichment the targets of a specific miRNA. For example, the targets of miR-148a in liver cancer are enriched in antigen processing and presentation KEGG Pathway (FDR = 0.006) (S1 Fig). In a practical sense, this means that this pathway is involved in liver cancer through a deregulation of miR-148a, and that this pathway could be, at least partially, modulated by modifying miR-148a expression. Other pathways involved in liver cancer that could be modulated by altering miRNA expression are RNA transport (FDR = 0.030), Cell cycle (FDR = 0.031) and Ubiquitin mediated proteolysis (FDR = 0.031) for the miR-424, or Lysine degradation (FDR = 0.006) for miR-29c.

### Integrative analysis of the miRComb miRNA-mRNA interactions from the 5 digestive cancers

#### Shared and specific miRNA-mRNA interactions

**Fig 4:**
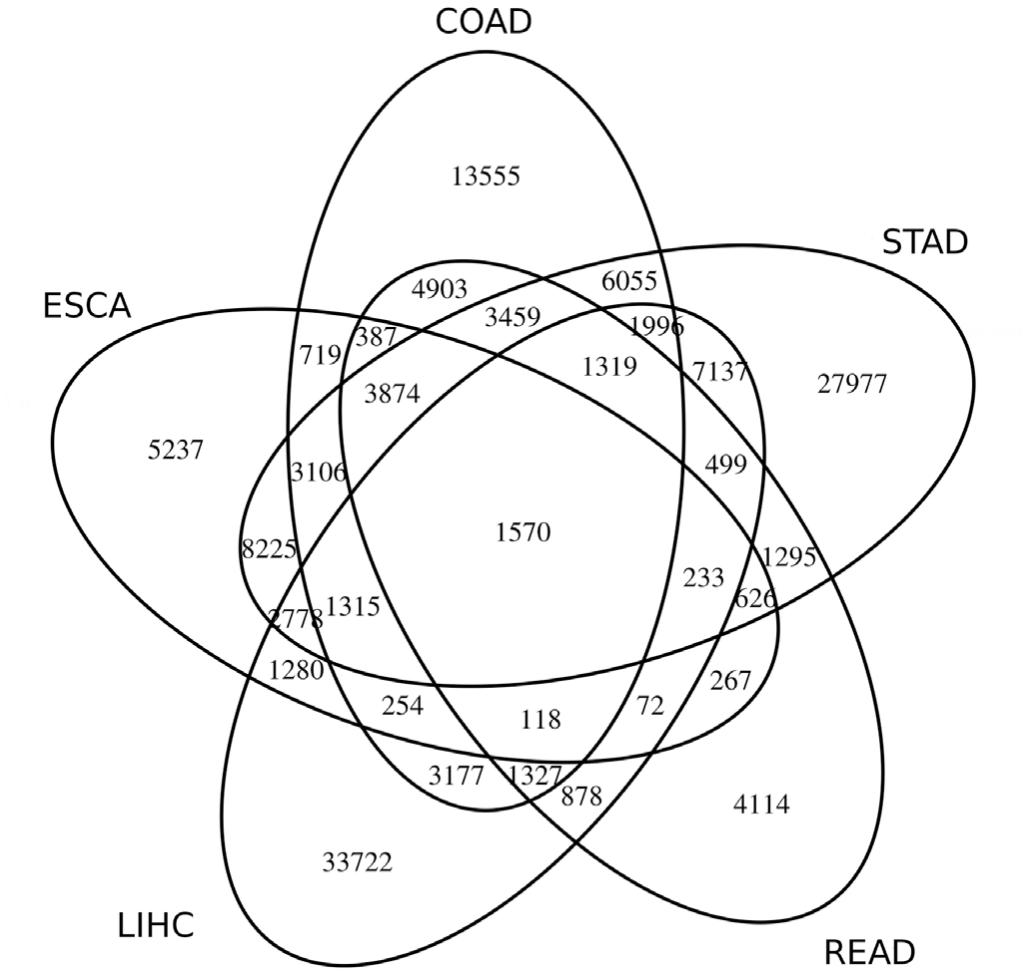
Venn diagram for miRComb miRNA-mRNA interactions between 5 digestive cancers. Venn diagram showing miRComb miRNA-mRNA interactions (FDR < 0.05 and predicted in at least one database) that are present in at least one cancer. 1570 miRNA-mRNA interactions appear in the 5 studied digestive cancers.

Fig 4 shows the number of shared miRComb miRNA-mRNA pairs among the 5 studied digestive cancer data sets. 1570 miRNA-mRNA interactions are shared for all 5 sets, but a more relevant number is shared in at least 2 or more of them, being only less than 40% of miRNA-mRNA pairs specific of each cancer data set. STAD is the one with more miRNA-mRNA interactions found.

In S2 Fig a network represents the 1570 common miRNA-mRNA interactions among the five studied mentioned data sets. We can see two networks: the big network on the left contains mostly downregulted miRNAs with their upregulated mRNA targets (780 miRNAs + mRNAs, and 1305 miRNA-mRNA pairs), while the smaller network on the right contains mostly upregulated miRNAs and their downregulated mRNA targets (173 miRNAs + mRNAs, and 187 miRNA-mRNA pairs). We have spotted the mRNAs that have KEGG terms related to cancer, such as Cell Cycle (red), Pathways in Cancer (yellow) and MAPK Signaling Patway (blue). Combinations of these terms are also displayed in different colours. The network on the right contains some mRNAs related to Cell Cycle, while the big on the left is mostly related to MAPK Signalling Pathway, Pathways in Cancer, or both terms (green).

The common interactions can be related to pathways that are shared by all the studied digestive cancers. However, it is also interesting to study the interactions that can be specific of each one. In S1 Table, all the specific miRComb miRNA-mRNA interactions for each cancer data set are shown (a specific interaction is the one that has been found significantly negatively correlated in one data set but not in the others). Tables 2–6 show the top 10 miRNAs with more miRComb miRNA-mRNA specific interactions for each cancer.

**Table 2:** Top 10 miRNAs sorted by number of specific targets in COAD. Target mRNAs are sorted according to its negative correlation value (top 20 are dislplayed).

miRNA	n.targets	mRNAs
**miR-30c**	264	MRAS, ZEB2, FCER1G, CFL2, SMNDC1, KIAA1949, CLIP4, SIGLEC5, C10orf119, CHORDC1, DZIP1L, LRCH2, SAP30, INTS2, ATF1, RPRD1A, PLXNC1, HCFC2, EIF5A2, TMEFF1
**miR-16**	213	TRANK1, KHNYN, PHLPP2, KIAA1370, C5orf41, ACOX1, CCDC88C, LCOR, CCNJL, SYNRG, CHD2, ZBTB34, SESN2, PDCD6IP, GCC2, MLL2, WNT5B, KIAA0317, NBR1, TNIP1
**miR-17**	178	TAOK3, GBF1, ZFYVE26, PPARD, TEP1, CYP26B1, KDM6B, BTBD7, CD68, NRBP1, NCOR1, KIAA1671, GOLGA2, ARHGAP21, MINK1, ALOX15B, KIAA1522, PSEN1, ARHGEF18, SEMA7A
**miR-454**	174	KIAA1211, HADHB, CSF1, FAM107B, PANK3, BTG1, ADAM28, FAM78A, MIER3, MXD1, BTD, RNASEL, MOBKL2B, GZMK, B2M, HADHA, TP53INP1, TCF7L2, DCTN2, TAGAP
**miR-106a**	173	SLC36A1, LASP1, TANC2, FGFR2, ANKFY1, BAHD1, KDM6B, SLC22A23, STAT3, CRK, C15orf17, TADA2B, ABHD5, MAP3K9, IQSEC1, ARHGEF11, NDEL1, CNNM2, KIAA1522, RCOR1
**miR-301a**	170	FAM107B, KIAA1211, ZDHHC7, MAML3, MXD1, MTF1, BTG1, RAB5B, PANK3, KLF3, LMTK2, MOBKL2B, NDEL1, ABHD5, C8orf4, FAM78A, LAMA3, KLHL20, HOXD1, TSPAN3
**miR-181c**	159	MAP3K6, ERI1, UGT8, KPNB1, SAP30, MORC4, SLC25A37, RNF125, PAX9, E2F7, ZIC2, WASF1, TUBB, PKNOX1, XKR9, MAP2K1, KITLG, XPO7, SLC25A4, C18orf55
**miR-539**	157	ZNF609, WNK1, YLPM1, HIVEP2, PHF3, MED1, DDX24, SPEN, INO80D, LRP6, SP1, SH3PXD2A, C10orf118, AHR, IWS1, SETD5, HNRNPK, RNMT, KIAA1244, LCOR
**miR-181a**	146	TM4SF19, POM121C, WDR45L, ACAN, SPP1, PROCR, ZNF207, PITPNB, EME1, STC1, RAN, MELK, EEF1E1, MRPS23, SCD, SAP30, TUBB, RNF8, CDCA4, SLC35B1
**miR-106b**	140	SMAD7, FAM102A, TEP1, NCOR1, SESN2, KIAA1522, PANK3, PPARD, GBF1, CRK, SLC22A23, WDR37, TRIM36, CYP26B1, ANKFY1, MYO1F, TMEM156, KIAA1671, MBNL3, MAP3K12

**Table 3:** Top 10 miRNAs sorted by number of specific targets in ESCA. Target mRNAs are sorted according to its negative correlation value (top 20 are dislplayed).

miRNA	n.targets	mRNAs
**miR-944**	313	SLC41A2, HNMT, LONRF3, GATA6, ARHGAP18, MGAT4A, ICA1, LPIN2, VPS13C, SLC12A2, NR3C2, HSD17B11, FOXP1, THRA, C2orf88, PTPRB, TMEM50B, C20orf112, C11orf54, SEPSECS
**miR-205**	261	ENPP4, SERPINA5, PTPRJ, SPATA13, BTNL8, SPRY1, ACACB, SLC4A4, PHF17, MGRN1, PTP4A2, MGAT4A, MAGI1, DOK1, EXOC6, PRDM16, NEK6, CASC4, HSD17B11, FBP1
**let-7b**	177	TTLL6, TFPI, TMEM135, SFMBT1, GALC, SLC46A3, CCL23, YPEL2, MUC3A, ITGB3, C7orf58, ATP8A1, SEC14L1, INSR, GXYLT1, BHMT2, KLF9, HGF, MLXIP, MAP4K3
**miR-338-5p**	141	SAFB, VRK2, NEDD1, ABHD12B, LIMK2, AEBP2, TANC2, QSER1, RAB38, CERS3, ROBO1, MBD2, SP3, SYPL1, SCPEP1, ATP2C1, UNK, CCNA1, FKBP3, NCK1
**miR-27a**	128	RALGPS1, NFATC2, EEPD1, PLEKHA6, MAN2A2, SPATA13, PPM1H, KIAA1958, FOXA3, PRDM16, KBTBD11, SEMA3B, PTPRJ, RALGAPA2, TRIM2, PPFIA2, KIAA1147, GPD1, CAPN9, NKTR
**miR-23a**	115	KLHDC7A, PTPRB, REPS2, LONRF3, ZC3H12B, ZNF420, C11orf75, FUCA1, TTC6, TBC1D12, RAB17, ZNF518A, MLPH, ZNF238, GPRC5B, C10orf68, CRBN, ZNF780B, ZNF506, ZNF253
**miR-34c-5p**	97	MLPH, TM9SF3, AHCYL2, CAPN5, LONRF3, CREB3L1, MYO7B, LGR4, C10orf81, BACE2, PARP4, MGAT4A, TGFBR2, IYD, MICAL2, LRCH1, FUT8, GOLPH3L, UBR1, TM9SF2
**miR-24**	87	MEGF11, NDST3, SNTB1, HNF1B, ATP6V0A2, AHI1, EPB41L1, SNED1, SLC12A3, C9orf96, ARHGDIG, C20orf112, FCGRT, TCAP, NLK, ARHGAP26, IDUA, SLC37A1, UBN2, SMPDL3B
**miR-27b**	87	ACAA2, PEAK1, ZFP36L2, JMJD1C, ARL14, GPD1, PLCL2, CTH, PDK4, PLEKHA6, ZC3H12B, PTPRB, GPR126, FOXA3, OXER1, NR2F2, KBTBD11, SLC46A3, PAPSS2, GORASP1
**miR-149**	81	PLEKHA6, GJB1, CREB3L3, ACHE, GAB2, GRK5, FZD5, GPR114, RILP, MIA, MMP15, RPH3AL, MUC5B, DENND3, MUC5AC, SEMA3B, C11orf86, BIN1, ANPEP, IGJ

**Table 4:** Top 10 miRNAs sorted by number of specific targets in LIHC. Target mRNAs are sorted according to its negative correlation value (top 20 are dislplayed).

miRNA	n.targets	mRNAs
**miR-122**	498	SLC9A1, G6PC3, PKM2, VPS24, TBC1D10B, NCDN, ZDHHC7, C9orf86, GYS1, CHST12, GIT1, DULLARD, ALDOA, PLEKHB2, ATN1, SLC10A3, SLC25A6, TMEM87A, LMNB2, GLG1
**miR-424**	454	APLN, AMIGO3, RECQL5, FAM189B, UBE2Q1, MXD3, SNRPC, BAT4, ZNHIT3, NSMCE2, TOMM20, MTX1, BCAP31, PUF60, E4F1, CDKN2A, DUS1L, NFKBIL1, TARBP1, DEDD
**miR-22**	451	FBXO46, RCC2, UTP18, NAT9, H2AFX, COPS7B, UBE2Z, PHF5A, MCM6, KIF18A, C17orf53, OLA1, POGK, WDR62, HNRNPH1, FAM49B, FBXL19, TPM3, ENTPD2, RFXANK
**miR-885-3p**	448	BMP1, KIAA0174, ACCN2, C9orf116, CCDC103, E2F4, CDK6, RARG, SP5, OTUD5, OSR1, RALY, EIF2B4, CLDN2, PRMT2, PLSCR3, CDYL2, GTF3C5, CCDC40, PPP1R12C
**miR-101**	444	LASS5, DNMT3A, NAP1L1, EZH2, RIT1, UCK2, SMARCA4, SUB1, C1orf77, KIAA1841, SMARCD1, RASD2, STK19, DSTYK, ATP6V1E1, ATP5G2, UBE2D2, MFSD6, C12orf34, EED
**miR-885-5p**	439	NKD1, ADAMTS9, C20orf196, CMIP, VLDLR, DNAL1, RPGRIP1L, AP2M1, CDYL2, HSPB8, MFSD5, AAK1, HIF1AN, LAMA5, WWTR1, LUZP6, TTC30A, RNASEL, CFLAR, CHMP5
**let-7c**	415	ARID3A, IGF2BP1, NAP1L1, PCBP4, NPEPL1, C7orf49, ABCC5, DLGAP4, ABCC10, BAX, SLC12A9, C15orf39, IRGQ, CYB561D1, IGF2BP3, FBXL19, GGA3, DUSP9, MMP11, AARSD1
**miR-125b**	399	SLC26A6, RBCK1, NUP210, NEU1, THOC5, P2RX4, ARID3A, ATP5G2, STK11IP, GLTP, LIMK1, MAZ, RIT1, PLXNA1, MAN1B1, CD2BP2, C15orf39, MSI1, RFXANK, TAZ
**miR-30e**	392	C8orf76, FKBP1A, MICAL1, DTX2, C19orf50, NME6, STK39, STOML1, DGKZ, TMC7, TTC39A, USF1, VOPP1, SEMA7A, TTC35, GNPDA1, FZD2, LENG9, AURKB, RPS19BP1
**miR-27b**	376	PSMD7, KIAA0513, HM13, EFNA3, WDR45, ACCN2, SLC7A11, WDR8, ATP6AP1, ELOVL1, SCAMP3, PIGT, MRPL33, BRSK1, KIAA0226, FAM21B, UNC45A, MEPCE, TSEN54, RRP12

**Table 5:** Top 10 miRNAs sorted by number of specific targets in READ. Target mRNAs are sorted according to its negative correlation value (top 20 are dislplayed).

miRNA	n.targets	mRNAs
**miR-323-3p**	262	KIAA0907, MYLIP, MACC1, RBM41, EFNA3, RBBP6, ABI1, TPR, TMEM106B, MLL5, PHF14, MKLN1, SLC25A36, AFTPH, NCBP2, ZNF292, RBM39, RSBN1, ZNF485, NCOA3
**miR-23a**	179	WASL, UBE2D1, MTM1, PLEKHM3, TMEM87B, PPP1R12A, CBLL1, WAC, MLLT4, CDC40, PTP4A2, AEBP2, RPRD2, RBBP6, CPEB2, TSR2, BMPR2, BACH2, PURB, ZYG11B
**miR-369-3p**	120	BMPR2, STYX, STON1, ZEB1, GOPC, RC3H1, RAB3GAP2, TMEM87B, PHACTR2, IQSEC1, GABPA, ZNF350, SEC63, TNRC6A, RAB11FIP2, UBE2J1, JHDM1D, VPS36, SMG1, OSTM1
**miR-382**	109	BTBD7, PRKAA1, NT5C2, FBXO28, DHX32, MBNL1, HIPK1, ZMYM2, MIER1, PLEKHA1, ZNF638, C3orf63, DDX3X, RSBN1L, ZNF197, FOXN2, CCDC132, PDE5A, C9orf68, CASP3
**miR-409-3p**	105	GPBP1L1, C9orf68, CCNT2, TCF7L2, CREB1, FANCL, ZNF14, ARHGAP5, CLK4, C5orf28, NSUN6, DPY19L4, PPHLN1, EBAG9, NDFIP2, ATXN3, TBL1XR1, SLC35F5, ZNF540, SAV1
**miR-23b**	101	NCOA6, EEA1, ADNP, TSR2, PAPD5, TAB3, TXLNG, FAM123B, IYD, ZNF81, FMR1, UBN2, WASL, GCC1, WIPF2, XIAP, ZBTB44, PICALM, KLHL15, SIAH1
**miR-381**	99	AKAP6, SORBS1, CACNB2, PBX1, ANK3, LMO3, MBNL1, ZFYVE21, BTBD7, SPPL3, TES, NBEA, MYST4, CHMP1B, ARHGAP5, CACNA1C, CASD1, KIAA1143, ADAMTSL3, RABGAP1
**miR-106a**	93	ZBTB6, GMCL1, CDC40, FAM3C, PHTF2, ZNF800, TBC1D15, HOOK3, PTP4A2, SLC4A7, LMBRD1, ZBTB41, CNOT6L, ITGB8, DEGS1, CMPK1, SNX16, SGTB, TMEM168, SNTB2
**miR-27a**	68	EGFR, STON1, CSF1, SERTAD2, MARCKS, HGSNAT, ATP2B1, SGMS1, C5orf41, SMCR8, SMCHD1, GPD2, SSH1, SEPN1, ARHGAP21, TICAM2, WIPF2, PLS1, DIRC2, C16orf54
**miR-409-5p**	62	ANKRD13C, MON2, TLK1, DYRK1A, PDE4D, FRS2, FAM129A, PDIK1L, RAB3GAP1, C9orf45, NBEA, ZBTB34, PRKAA2, USP15, ARID4B, SFRS11, ENSA, KIAA1598, BRAP, MKL2

**Table 6:** Top 10 miRNAs sorted by number of specific targets in STAD. Target mRNAs are sorted according to its negative correlation value (top 20 are dislplayed).

miRNA	n.targets	mRNAs
**miR-330-3p**	390	PRUNE2, NFIA, LMOD3, PARVA, TMEM35, KANK2, ZNF25, HCFC2, FOXP2, ATP2B4, PDE5A, TEAD1, HOXA3, DPYSL3, RNF180, NRP2, TSHZ3, SMAD9, DDR2, SHISA9
**miR-26a**	357	KIAA1737, UBR3, RANBP9, TMEM106B, G3BP2, KPNA6, ZNF148, STXBP4, ZYG11B, FAM8A1, HEATR5A, UBE2H, UBE2G1, RLF, PEX13, UBR1, SCAMP1, AHI1, LIMS1, FBXW2
**miR-1**	326	PIGW, UHMK1, CAPRIN1, MTHFD1, NXT2, POLA1, PHF6, CMTM8, AZIN1, SMG7, HOOK1, TMED5, SLC39A9, FAF2, NUP54, IPO9, SMCR7L, PASK, SF3B3, SPTLC1
**miR-340**	319	LPP, VEZF1, ETV1, RBFOX2, NEK7, SLC25A12, SLC20A2, VAMP4, SGMS2, FBXO8, ZCWPW2, TEAD1, VCL, FAT3, DIXDC1, NCAM2, SGCD, CALD1, MACF1, FBXO3
**let-7g**	315	RBFOX2, SLC8A2, DMD, CPEB1, GHR, KLHL4, NEFM, HLF, WNK3, DOCK3, FGF5, LEPR, NFASC, TGFBR3, KLF8, KIAA2022, EZH1, NOVA1, PBX1, FOXN3
**miR-129-5p**	314	TMEM62, COL11A1, NXT2, C6orf223, WDFY1, FCGR1A, DTL, NOX1, TRIAP1, PRPF40A, WDR12, TGIF2, CACYBP, SLBP, ALG6, MRPL13, TPM3, RPIA, NDUFA10, E2F7
**let-7f**	313	ACTR10, FGF5, MAP4K3, BACH1, PPAPDC1A, SNX6, RBFOX2, CALM1, DPH3, CALU, SESTD1, SLMAP, BAG2, CRBN, ELOVL4, SGCD, COPS4, FBXO32, PRKAB2, KPNA4
**miR-29a**	310	DIP2C, IL17RD, DNAL1, RMND5A, TGFB2, BACE1, FBXL20, PRICKLE2, ATP2B4, ILDR2, OXTR, SBF2, RYBP, PCYT1B, CALU, CACNA1C, C16orf72, CDKL2, KIF5A, JAZF1
**miR-15b**	290	FOXP2, NOS1, GRPR, KATNAL1, TEAD1, ANKRD53, GPR135, PENK, KY, WNK3, PRTG, CHIC1, TLE4, BAI1, AASS, KCNQ5, BCL2, SYDE2, PID1, BMPR1A
**miR-30b**	286	VSTM4, TEAD1, AFF4, ABCC9, BCL6, KLF11, ZYG11B, PRKAR1A, UBE2G1, EPN2, C3orf58, ZCCHC24, CCDC6, PCDH10, SETD7, AMOTL2, YPEL2, SAMD4A, ZNF264, PHACTR2

**Fig 5:**
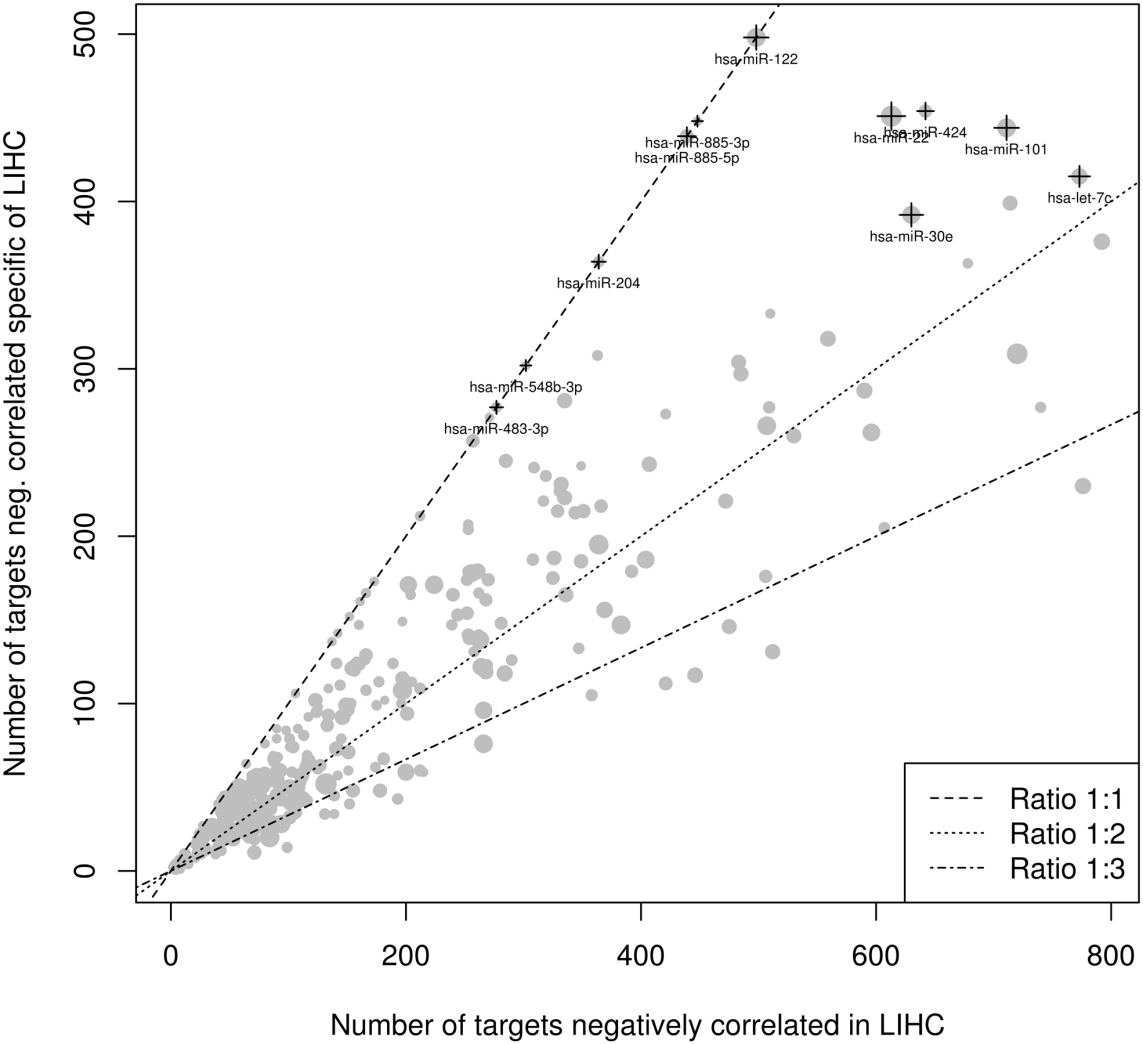
Specificity of MiRNA-mRNA interactions in LIHC. Number of total miRNA targets in LIHC versus number of miRNA targets present only in LIHC but not in COAD, ESCA, READ or STAD. Size of the points is proportional to the mean miRNA expression on the LIHC samples included.

Fig 5 also shows the number of specific interactions depending on the miRNAs involved in LIHC. MiRNAs on the line corresponding to ratio 1:1 are those that are only expressed in liver. The others are expressed in at least another cancer, but they have some specific interactions in LIHC, the closer to the ratio 1:1 line are, the higher specificity is.

#### Cluster analysis of miRNA-mRNA interactions

**Fig 6:**
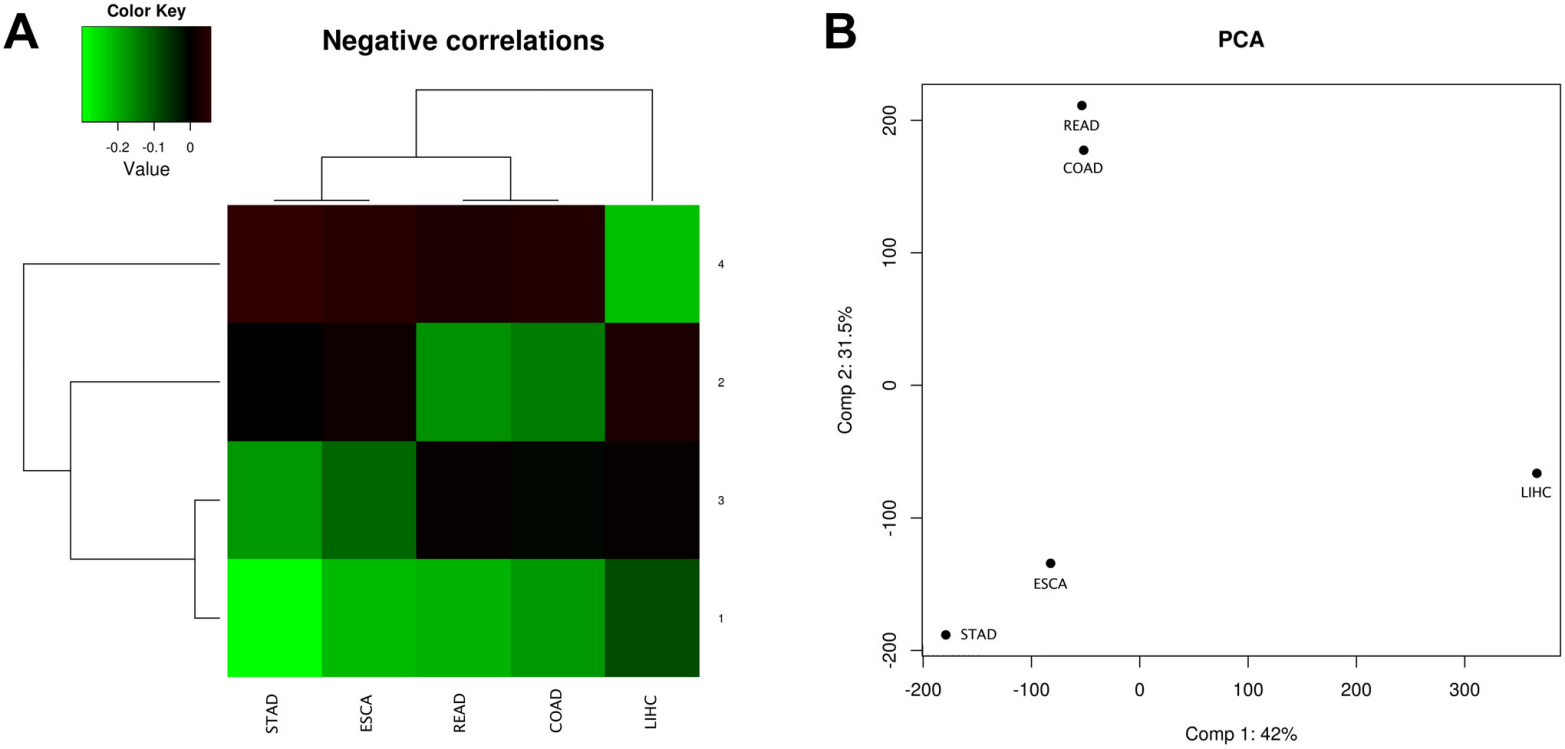
Clustering and Principal Components Analysis of the five digestive cancers. Computations are based on the correlation coefficients of the 106.426 miRNA-mRNA pairs that are expressed across all five cancer data sets. A) Heatmap showing the centers of the different clusters. Values represent the mean of the Pearson correlation coefficient of the miRNA-mRNA pairs that fall into the cluster. B) Principal Components Analysis (based on correlation matrix) of the Pearson correlation coefficient of the miRNA-mRNA pairs from the five digestive cancer data sets.

Globally, there are 106.426 miRNA-mRNA interactions measured in all cancer data sets, and significantly negatively correlated in at least one of them. In order to classify them into similar patterns, we applied clustering methods in order to summarize the main trends. We used the K-means method with 4 clusters as it gave a reasonable interpretation of the results (Fig 6). Interestingly, hierarchical clustering of cancers according to the mean correlation coefficients of the clusters gives the following result: STAD and ESCA are first grouped, as well as READ and COAD. Next, these four cancers are grouped, and finally LIHC is added to the tree. Principal Components Analysis shows the same pattern. It is an expected result and is reasonable with biological similarities of these tumors. Successive increase of the number of clusters allow to differentiate other trends according to the correlations (data not shown), but the tree structure described before (COAD+READ, ESCA+STAD and then LIHC) is always maintained.

Clusters can be interpreted as follows: Cluster 1: miRNA-mRNA interactions slightly negatively regulated across all cancers and interactions that do not fit other clusters; Cluster 2: miRNA-mRNA interactions negatively correlated in COAD and READ, but not in the other cancers; Cluster 3: miRNA-mRNA interactions negatively regulated in ESCA and STAD, but not in the other cancers; Cluster 4: miRNA-mRNA interactions negatively regulated in LIHC, but not in the other cancers. For example, miR-106a and its targets are quite specific of Cluster 2 –COAD and READ- (although they are also present in some extent in Cluster 1 –all cancers-). Another example, miR-29c targets are specific from Clusters 3 –LIHC and ESCA- and 4 –LIHC-, and have almost no presence in Cluster 2. Furthermore, miR-22 targets are specific from Cluster 4 –LIHC-, and others such as miR-30b or let-7b targets seem to not show any clear specificity (S3 Fig).

## Conclusions

The miRComb R package is structured in functions that use well-established statistical concepts and translates complex biological processes to statistical tests in a comprehensive way. That structure allows changing any step of the workflow and adapting it to further improvements making it an easy updatable tool.

MiRComb R package implements a method that elucidatesthe most suitable miRNA-mRNA interactions that are involved in a specific disease and helps us to interpret them in that biological context. Moreover, it presents the results in a standardised way (pdf report). Interesting results could be obtained from the reports, meaning that they are useful to understand gene regulation in that specific biological context.

Integrative analysis of the five studied digestive cancers revealed some similarities regarding miRNA-mRNA interactions between all cancer data sets (COAD, READ, ESCA, STAD and LIHC). However, COAD and READ; and ESCA and STAD, shared respectively much more interactions between them than with LIHC. This observation leads to a reasonable result linking the miRNA-mRNA signatures obtained from miRComb to the pathophysiology of each cancer. Furthermore, this integrative analysis allowed us to determine the specific pattern of miRNA-mRNA interactions across the five studied cancers.

The huge amount of information regarding miRNA-mRNA interactions generated in this study provides the basis for developing many testable hypotheses that could be the starting point for setting up multiple challenging projects aimed to understand gene regulation in these cancers or, even, aimed to discover new therapeutic targets.

## Supporting Information

S1 Fig KEGG Pathway for hsa-miR-148a targets in liver cancer: Antigen Processing and Presentation. Hsa-miR-148a targets (negative correlation with hsa-miR-148a (FDR < 0.05) and predicted in at least one database) are highlighted in red.
(PNG)

S2 Fig Network for common miRComb miRNA-mRNA interactions across five digestive cancers. Figure showing the common 1570 miRComb miRNA-mRNA interactions across all studied cancers. Circles are mRNAs, while diamonds are miRNAs. Fill color represents in which pathways the resulting protein of the mRNA is involved. Yellow: Pathways in cancer; Blue: MAPK signalling pathway; Red: Cell Cycle; Green: Pathways in cancer +MAPK signalling pathway; Orange: Pathways in cancer+Cell cycle; Magenta: MAPK signalling pathway+Cell cycle; Grey: MAPK signalling pathway+ Pathways in cancer+Cell cycle.
(EPS)

S3 Fig Targets distribution by clusters. Number of miRNA targets in each cluster. The represented miRNAs are the top 50 sorted by the total number of targets. Blue: Cluster 1; Green: Cluster 2; Magenta: Cluster 3; Orange: Cluster 4.
(ZIP)

S1 File MiRComb pdf reports from colon cancer TCGA data set. The report has been made by mkReport function.
(PDF)

S2 File MiRComb pdf report from rectum cancer TCGA data set. The report has been made by mkReport function.
(PDF)

S3 File MiRComb pdf report from esophagus cancer TCGA data set. The report has been made by mkReport function.
(PDF)

S4 File MiRComb pdf report from stomach cancer TCGA data set. The report has been made by mkReport function.
(PDF)

S5 File MiRComb pdf report from liver cancer TCGA data sets. The report has been made by mkReport function.
(PDF)

S1 Table Excel table showing specific miRNA-mRNA interactions for each digestive cancer compared to the remaining four. Columns: miRNA: name of the miRNA; mRNA: name of the mRNA; cor: value of the Pearson Correlation estimate between the expression of the miRNA and the mRNA; pval: p value of the Pearson Correlation estimate; adj.pval: p value of the Pearson Correlation estimate corrected for multiple testing (Benjamini & Hochberg method applied); logratio.miRNA: log-ratio of the miRNA in the comparison between Cancer vs Healthy samples; logratio.mRNA: log-ratio of the mRNA in the comparison between Cancer vs Healthy samples; meanExp.miRNA: mean expression of the miRNA across all samples; meanExp.mRNA: mean expression of the mRNA across all samples; dat.microCosm_v5_18: “1” if the miRNA-mRNA pair is a computationally predicted miRNA-mRNA pair according to microCosm database, “0” if not; dat.targetScan_v6.2_18: “1” if the miRNA-mRNA pair is a computationally predicted miRNA-mRNA pair according to TargetScan database, “0” if not; dat.sum: colum that sums the values of columns dat.microCosm_v5_18 and dat.targetScan_v6.2_18;; score: score (see section 2.5) of the miRNA-mRNA, according their log-ratios.
(XLSX)

## Abbreviations

BH: Benjamini & Hochberg
COAD: colon adenocarcinoma
ESCA: esophageal carcinoma
FDR: False Discovery Rate
H: healthy
LIHC: liver hepatocellular carcinoma
MiRNA: microRNA
MRNA: messenger RNA
qRT-PCR: quantitative reverse transcription PCR
NGS: Next-Generation Sequencing
READ: rectum adenocarcinoma
STAD: stomach adenocarcinoma
TCGA: The Cancer Genome Atlas

## References

[1] Guo H, Ingolia NT, Weissman JS, Bartel DP. Mammalian microRNAs predominantly act to decrease target mRNA levels. *Nature* 466, 835–840 (2010). 10.1038/nature09267 20703300 PMC2990499

[2] Gade S, Porzelius C, Fälth M, Brace JC, Wuttig D, Binder H, et al Graph based fusion of miRNA and mRNA expression data improves clinical outcome prediction in prostate cancer. *BMC Bioinformatics* 12, 488 (2011). 10.1186/1471-2105-12-488 22188670 PMC3471479

[3] Peng X, Li Y, Walters KA, Rosenzweig ER, Lederer SL, Aicher LD, et al Computational identification of hepatitis C virus associated microRNA-mRNA regulatory modules in human livers. *BMC Genomics* 10, 373 (2009). 10.1186/1471-2164-10-373 19671175 PMC2907698

[4] R Core Team. R: A Language and Environment for Statistical Computing. (2015). at <http://www.R-project.org>

[5] Huber W, Carey VJ, Gentleman R, Anders S, Carlson M, Carvalho BS, et al Orchestrating high-throughput genomic analysis with Bioconductor. *Nat Meth* 12, 115–121 (2015). 10.1038/nmeth.3252 PMC4509590 25633503

[6] Cogswell JP, Ward J, Taylor IA, Waters M, Shi Y, Cannon B, et al Identification of miRNA Changes in Alzheimer’s Disease Brain and CSF Yields Putative Biomarkers and Insights into Disease Pathways. *J*. *Alzheimers Dis*. 14, 27–41 (2008). 18525125 10.3233/jad-2008-14103

[7] Ru Y, Kechris KJ, Tabakoff B, Hoffman P, Radcliffe RA, Bowler R, et al The multiMiR R package and database: integration of microRNA–target interactions along with their disease and drug associations. *Nucleic Acids Res*. gku631 (2014). 10.1093/nar/gku631 PMC4176155 25063298

[8] International Cancer Genome Consortium, Hudson TJ, Anderson W, Artez A, Barker AD, Bell C, et al International network of cancer genome projects. *Nature* 464, 993–998 (2010). 10.1038/nature08987 20393554 PMC2902243

[9] LaTeX3 Project. LaTeX—A document preparation system. at <www.latex-project.org>

[10] Leisch, F. in Compstat (eds. Härdle, P. D. W. & Rönz, P. D. B.) 575–580 (Physica-Verlag HD, 2002). at <http://link.springer.com/chapter/10.1007/978-3-642-57489-4_89>

[11] Law CW, Chen Y, Shi W, Smyth GK. voom: precision weights unlock linear model analysis tools for RNA-seq read counts. *Genome Biol*. 15, R29 (2014). 10.1186/gb-2014-15-2-r29 24485249 PMC4053721

[12] Johnson WE, Li C, Rabinovic A. Adjusting batch effects in microarray expression data using empirical Bayes methods. *Biostatistics* 8, 118–127 (2007). 16632515 10.1093/biostatistics/kxj037

[13] Hong F, Breitling R, McEntee CW, Wittner BS, Nemhauser JL, Chory J. RankProd: a bioconductor package for detecting differentially expressed genes in meta-analysis. *Bioinformatics* 22, 2825–2827 (2006). 16982708 10.1093/bioinformatics/btl476

[14] Peterson SM, Thompson JA, Ufkin ML, Sathyanarayana P, Liaw L, Congdon CB. Common features of microRNA target prediction tools. *Bioinforma*. *Comput*. *Biol*. 5, 23 (2014). 10.3389/fgene.2014.00023 PMC3927079 24600468

[15] Huang JC, Babak T, Corson TW, Chua G, Khan S, Gallie BL, et al. Using expression profiling data to identify human microRNA targets. *Nat*. *Methods* 4, 1045–1049 (2007). 18026111 10.1038/nmeth1130

[16] Griffiths-Jones S, Grocock RJ, van Dongen S, Bateman A, Enright AJ. miRBase: microRNA sequences, targets and gene nomenclature. *Nucleic Acids Res*. 34, D140–D144 (2006). 16381832 10.1093/nar/gkj112 PMC1347474

[17] Rehmsmeier M, Steffen P, Höchsmann M, Giegerich R. Fast and effective prediction of microRNA/target duplexes. *RNA* 10, 1507–1517 (2004). 15383676 10.1261/rna.5248604 PMC1370637

[18] Lewis BP, Burge CB, Bartel DP. Conserved Seed Pairing, Often Flanked by Adenosines, Indicates that Thousands of Human Genes are MicroRNA Targets. *Cell* 120, 15–20 (2005). 15652477 10.1016/j.cell.2004.12.035

[19] Hofacker IL, Fontana W, Stadler PF, Bonhoeffer LS, Tacker M, Schuster P. Fast folding and comparison of RNA secondary structures. *Monatshefte Für Chem*. *Chem*. *Mon*. 125, 167–188 (1994).

[20] Shannon P, Markiel A, Ozier O, Baliga NS, Wang JT, Ramage D, et al Cytoscape: A Software Environment for Integrated Models of Biomolecular Interaction Networks. *Genome Res*. 13, 2498–2504 (2003). 14597658 10.1101/gr.1239303 PMC403769

[21] Falcon S, Gentleman R. Using GOstats to test gene lists for GO term association. *Bioinformatics* 23, 257–258 (2007). 17098774 10.1093/bioinformatics/btl567

[22] Schröder MS, Gusenleitner D, Quackenbush J, Culhane AC, Haibe-Kains B. RamiGO: an R/Bioconductor package providing an AmiGO Visualize interface. *Bioinformatics* 29, 666–668 (2013). 10.1093/bioinformatics/bts708 23297033 PMC3582261

[23] Gu Z, Gu L, Eils R, Schlesner M, Brors B. circlize implements and enhances circular visualization in R. *Bioinformatics* 30, 2811–2812 (2014). 10.1093/bioinformatics/btu393 24930139

[24] Bandiera S, Pfeffer S, Baumert TF, Zeisel MB. miR-122 –A key factor and therapeutic target in liver disease. *J*. *Hepatol*. 62, 448–457 (2015). 10.1016/j.jhep.2014.10.004 25308172

