# Supplementary figures and images for: MiRComb: An R Package to Analyse miRNA-mRNA Interactions. Examples across Five Digestive Cancers
Source: PLoS One. 2016 Mar 11;11(3):e0151127. doi: 10.1371/journal.pone.0151127 (PMC4788200; doi:10.1371/journal.pone.0151127)

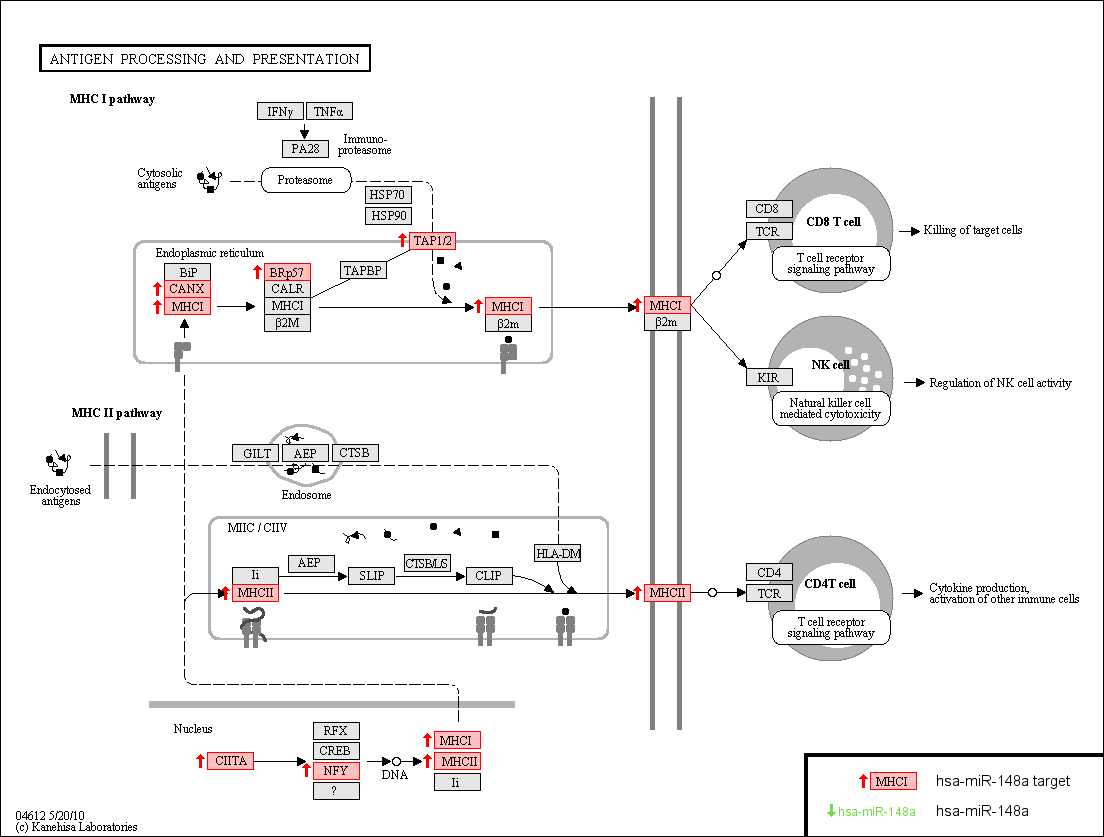

Supplement: S1 Fig — Hsa-miR-148a targets (negative correlation with hsa-miR-148a (FDR < 0.05) and predicted in at least one database) are highlighted in red. (PNG) [file pone.0151127.s001.png]
