# Supplementary material for: MiRComb: An R Package to Analyse miRNA-mRNA Interactions. Examples across Five Digestive Cancers
Source: PLoS One. 2016 Mar 11;11(3):e0151127. doi: 10.1371/journal.pone.0151127 (PMC4788200; doi:10.1371/journal.pone.0151127)
Supplement: S1 File — The report has been made by mkReport function. (PDF) [file pone.0151127.s004.pdf]

Default miRComb output

/home/mvila/Baixades/TCGA/colon

May 13, 2015

1 Exploratory analysis of miRNA dataset

Number of miRNAs analysed	325
Number of samples	444

Table 1: Basic information of the miRNA dataset.

	group.n	CvH	center	sample	batch
1	NT: 8	Min. :0.000	AA :160	TCGA-5M-AAT4-01: 1	Batch 116: 48
2	TP:436	1st Qu.:1.000	A6 : 57	TCGA-5M-AAT5-01: 1	Batch 41 : 48
3		Median :1.000	CM : 37	TCGA-5M-AAT6-01: 1	Batch 138: 46
4		Mean :0.982	D5 : 31	TCGA-5M-AATA-01: 1	Batch 89 : 42
5		3rd Qu.:1.000	G4 : 27	TCGA-5M-AATE-01: 1	Batch 76 : 38
6		Max. :1.000	DM : 25	TCGA-A6-2670-01: 1	Batch 28 : 33
7			(Other):107	(Other) :438	(Other) :189

Table 2: Summary of the phenotypical information of the miRNA dataset.

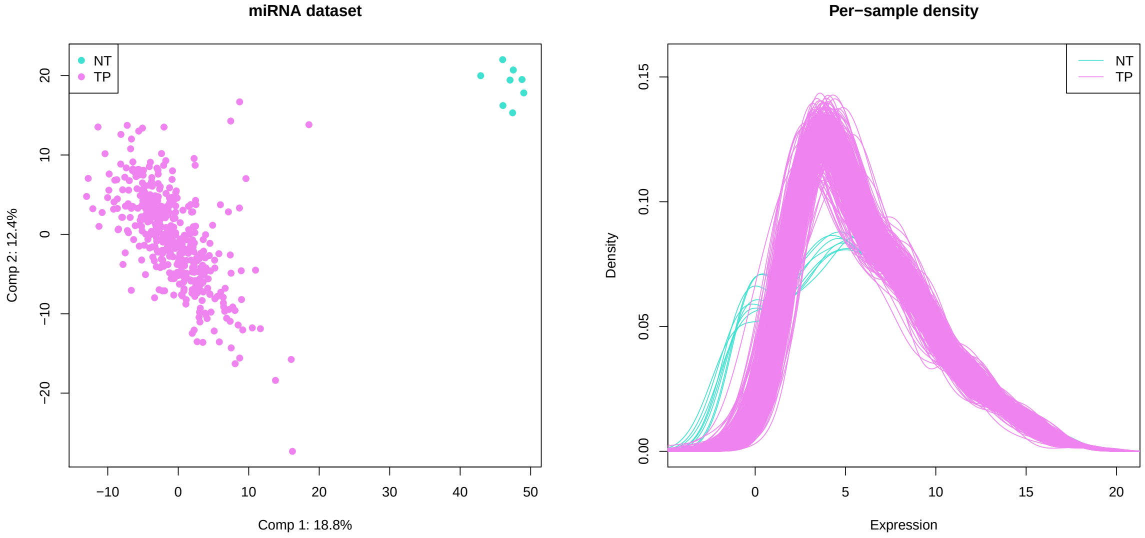

Figure 1: PCA and density plot for miRNAs.

2 Exploratory analysis of mRNA dataset

Number of mRNAs analysed	14860
Number of samples	444

Table 3: Basic information of the mRNA dataset.

	group.n	CvH	center	sample	batch
1	NT: 8	Min. :0.000	AA :160	TCGA-5M-AAT4-01: 1	Batch 116: 48
2	TP:436	1st Qu.:1.000	A6 : 57	TCGA-5M-AAT5-01: 1	Batch 41 : 48
3		Median :1.000	CM : 37	TCGA-5M-AAT6-01: 1	Batch 138: 46
4		Mean :0.982	D5 : 31	TCGA-5M-AATA-01: 1	Batch 89 : 42
5		3rd Qu.:1.000	G4 : 27	TCGA-5M-AATE-01: 1	Batch 76 : 38
6		Max. :1.000	DM : 25	TCGA-A6-2670-01: 1	Batch 28 : 33
7			(Other):107	(Other) :438	(Other) :189

Table 4: Summary of the phenotypical information of the mRNA dataset.

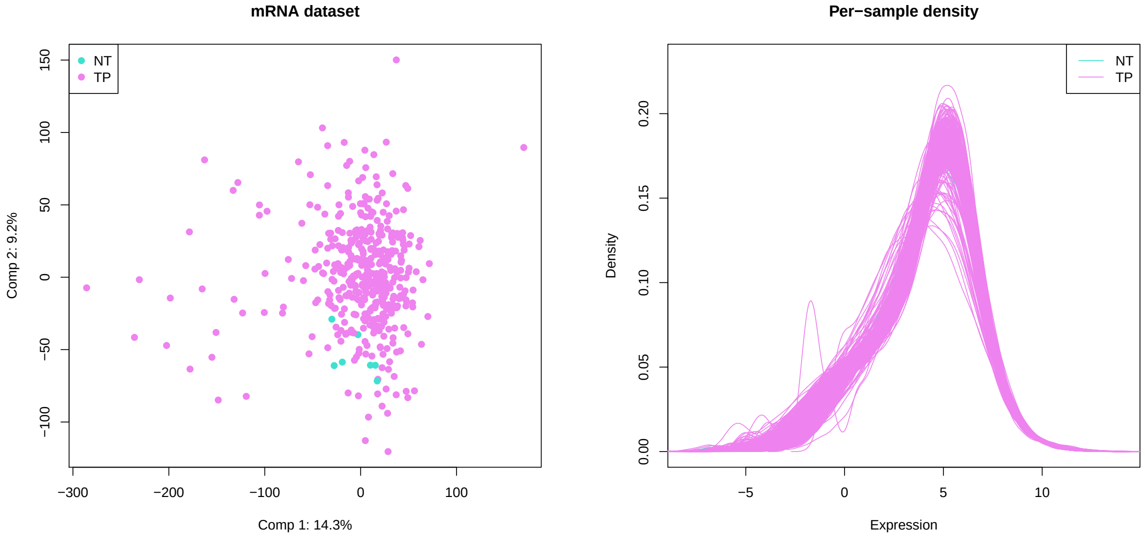

Figure 2: PCA and density plot for mRNAs.

3 Differentially expressed miRNAs

Analysis performed	Comparative used: CvH; method used: limma.
Number of differentially expressed miRNAs	325 (187 upregulated, 138 downregulated)
Number of samples	444
Criteria for selecting miRNAs	adj.pval < 1

Table 5: Basic statistics

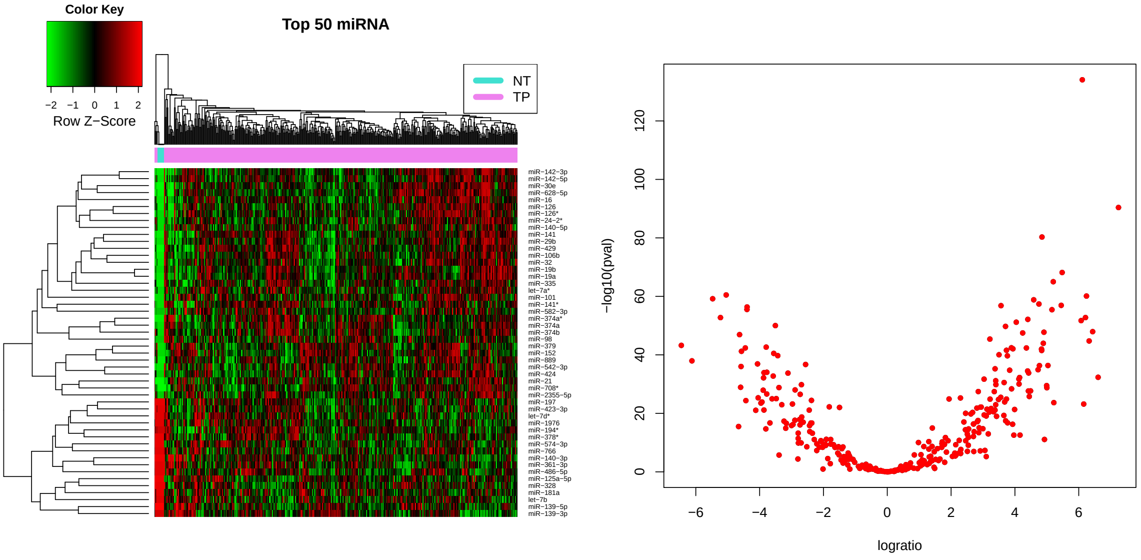

Figure 3: A) Heatmap vith the top 50 most significant miRNAs (sorted by adjusted p-value). B) Volcano plot showing the selected miRNAs.

4 Differentially expressed mRNAs

Analysis performed	Comparative used: CvH; method used: limma.
Number of differentially expressed mRNAs	14860 (8526 upregulated, 6334 downregulated)
Number of samples	444
Criteria for selecting mRNAs	adj.pval < 1

Table 6: Basic statistics

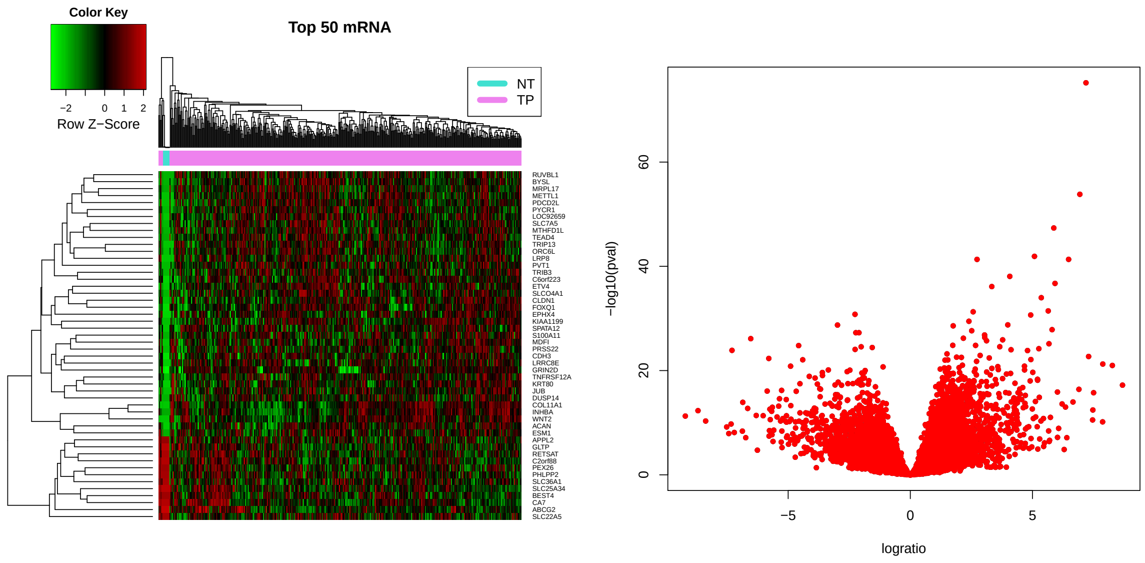

Figure 4: A) Heatmap vith the top 50 most significant mRNAs (sorted by adjusted p-value). B) Volcano plot showing the selected mRNAs.

5 Correlation & intersection with databases

Number of miRNAs	325
Number of mRNAs	14860
Total miRNA-mRNA combinations	4829500
Number of samples	444

Table 7: Number of miRNAs, mRNAs and samples used for correlation.

	Number	%
Total correlations	4829500	100
Total negative correlations	2363105	48.93
Total correlations p<0.05	1205347	24.96
Total correlations p<0.01	849917	17.6
Total correlations adj.p<0.05	823121	17.04
Total correlations adj.p<0.01	568570	11.77

Table 8: Basic statistics for correlation results. Correlation hypothesis: two.sided.

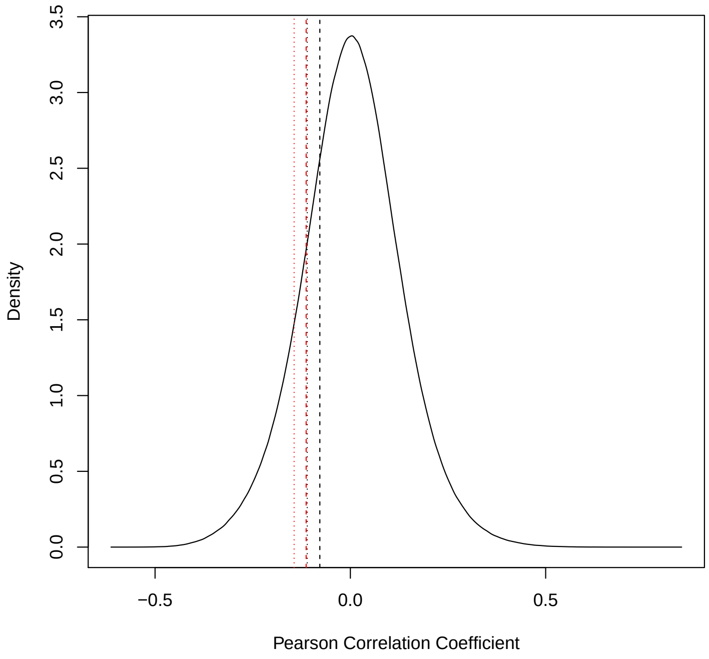

Figure 5: Density of a total of 4829500 miRNA-mRNA pairs. Dashed lines distinguish correlations whose p-value is lower than 0.05, dotted lines for 0.01. Black is for raw p-value and red for adjusted p-value.

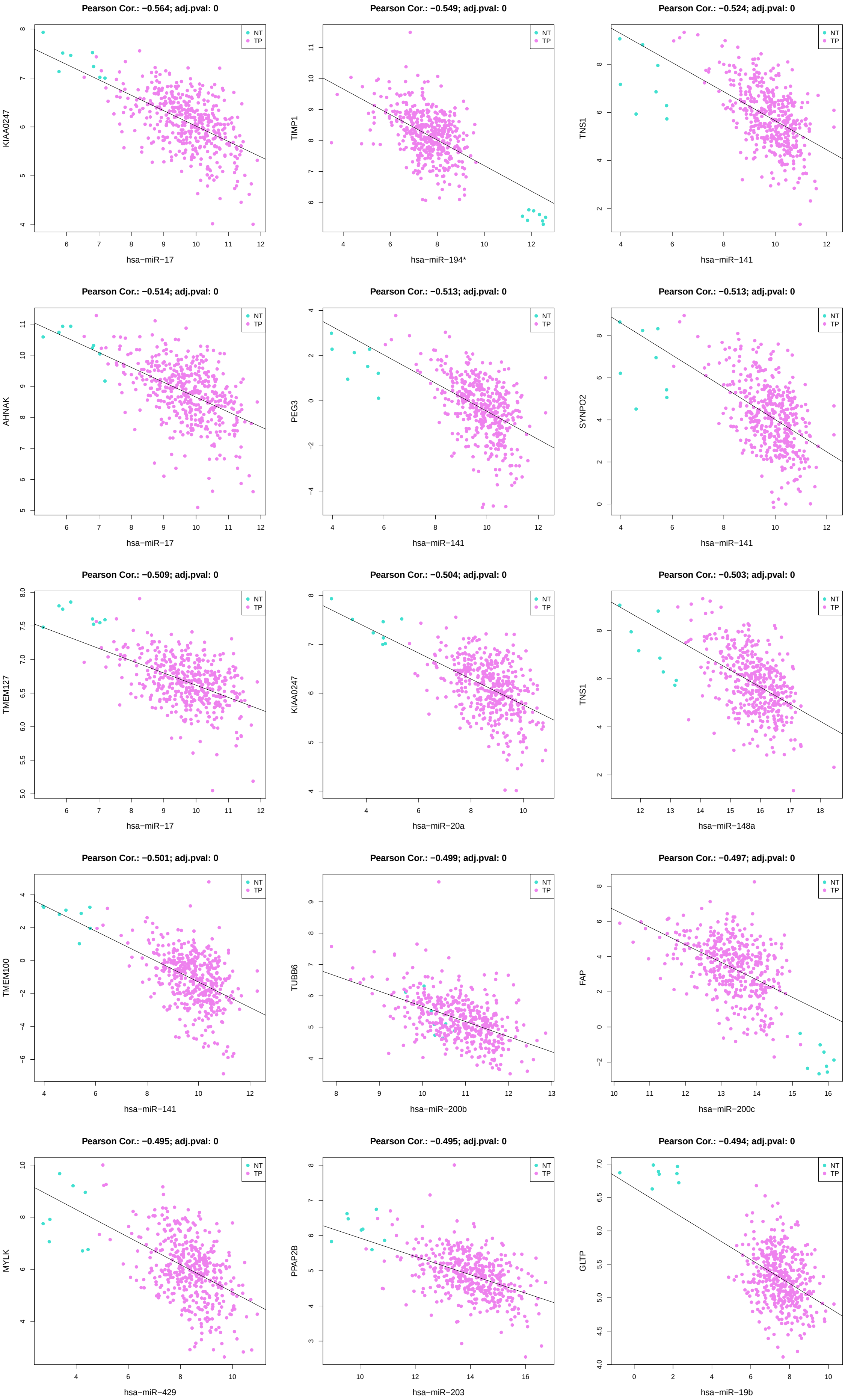

Figure 6: Plot of 15 top correlations, sorted by adjusted p-value. Databases used: microCosm_v5_18, targetScan_v6.2_18 (each miRNA-mRNA pair has to appear at least 1 times).

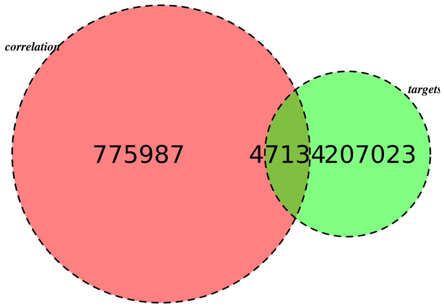

Figure 7: Venn Diagram. Left (red): number of miRNA-mRNA pairs with ajdusted p-value<0.05. Right (green): number of all the theoretical miRNA-mRNA pairs reported at least 1 times in the following databases: microCosm_v5_18, targetScan_v6.2_18. Intersection: miRNA-mRNA pairs that fulfil both conditions.

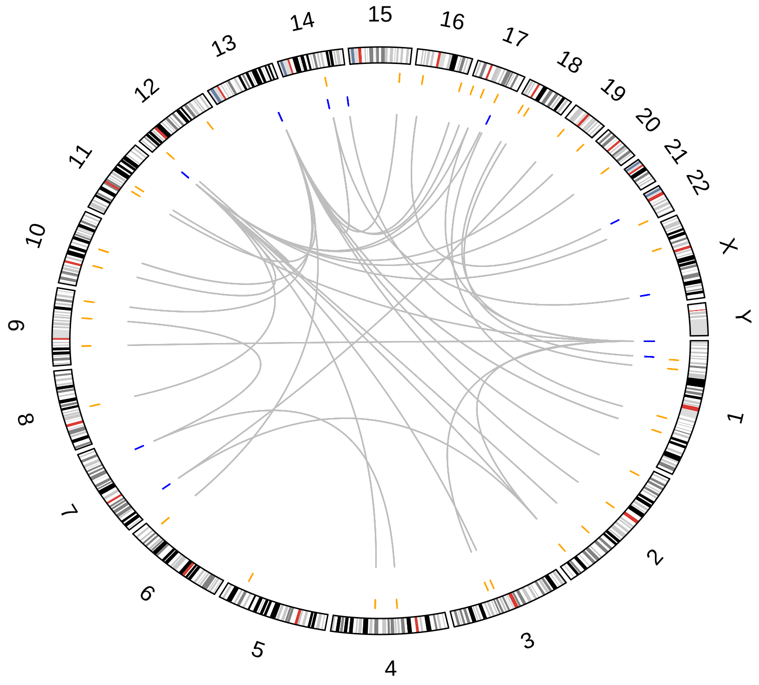

Figure 8: Circos plot for the first 45 miRNA-mRNA pairs (sorted by adjusted p-value) that have: pval-corrected<0.05 and appear at least 1 times in the following databases: microCosm_v5_18, targetScan_v6.2_18. Blue: miRNAs, Orange: target mRNAs

miRNA	mRNA	cor	adj.pval	FC.miRNA	FC.mRNA	dat.sum
hsa-miR-17	KIAA0247	-0.56	8.86e-33	10.38	-2.39	1
hsa-miR-194*	TIMP1	-0.55	5.52e-31	-23.96	6.31	1
hsa-miR-141	TNS1	-0.52	5.03e-28	28.51	-2.68	1
hsa-miR-17	AHNAK	-0.51	7.18e-27	10.38	-3.01	1
hsa-miR-141	PEG3	-0.51	1.07e-26	28.51	-4.02	1
hsa-miR-141	SYNPO2	-0.51	1.13e-26	28.51	-5.63	1
hsa-miR-17	TMEM127	-0.51	2.80e-26	10.38	-1.98	1
hsa-miR-20a	KIAA0247	-0.50	1.17e-25	21.55	-2.39	1
hsa-miR-148a	TNS1	-0.50	1.33e-25	10.59	-2.68	1
hsa-miR-141	TMEM100	-0.50	2.30e-25	28.51	-14.32	1
hsa-miR-200b	TUBB6	-0.50	3.87e-25	1.48	-1.03	1
hsa-miR-200c	FAP	-0.50	5.81e-25	-5.41	36.99	1
hsa-miR-429	MYLK	-0.50	9.52e-25	27.25	-4.06	1
hsa-miR-203	PPAP2B	-0.49	1.10e-24	15.89	-2.47	1
hsa-miR-19b	GLTP	-0.49	1.15e-24	75.71	-2.95	1
hsa-miR-592	PRDM8	-0.49	1.50e-24	30.41	-1.27	1
hsa-miR-592	DAPK1	-0.49	2.05e-24	30.41	-2.11	1
hsa-miR-200a	TNS1	-0.49	3.15e-24	9.14	-2.68	1
hsa-miR-17	FAM129A	-0.49	3.60e-24	10.38	-4.18	1
hsa-miR-141	LMO3	-0.49	4.47e-24	28.51	-6.79	1
hsa-miR-17	ZBTB4	-0.49	5.14e-24	10.38	-1.29	1
hsa-miR-17	GSN	-0.49	5.14e-24	10.38	-2.98	1
hsa-miR-106a	KIAA0247	-0.49	6.09e-24	8.97	-2.39	1
hsa-miR-200c	FAM19A5	-0.49	6.70e-24	-5.41	4.10	1
hsa-miR-200b	DNAJB5	-0.49	8.22e-24	1.48	-1.86	1
hsa-miR-200b*	P4HA3	-0.48	1.58e-23	-3.84	6.41	1
hsa-miR-17	KCNMA1	-0.48	1.90e-23	10.38	-5.31	1
hsa-miR-148a	CNN1	-0.48	2.98e-23	10.59	-3.92	1
hsa-miR-141	CCDC80	-0.48	3.02e-23	28.51	-2.29	2
hsa-miR-194*	FHL3	-0.48	3.62e-23	-23.96	2.31	1
hsa-miR-200c	RAB34	-0.48	4.16e-23	-5.41	-1.05	1
hsa-miR-625*	C20orf43	-0.48	4.50e-23	-2.55	1.33	1
hsa-miR-17	KIAA0513	-0.48	4.50e-23	10.38	-4.91	1
hsa-miR-200c	DOK5	-0.48	7.01e-23	-5.41	1.72	1
hsa-miR-200b	RAB34	-0.48	8.66e-23	1.48	-1.05	1
hsa-miR-16	SLC36A1	-0.47	4.55e-22	15.32	-4.79	1
hsa-miR-141	CXCL12	-0.47	4.68e-22	28.51	-5.30	1
hsa-miR-21*	PBXIP1	-0.47	5.59e-22	5.48	-1.83	1
hsa-miR-17	AKAP13	-0.47	5.99e-22	10.38	-1.81	1
hsa-miR-20a	FAM129A	-0.47	6.62e-22	21.55	-4.18	1
hsa-miR-552	KCTD1	-0.47	8.67e-22	8.58	-1.32	1
hsa-miR-130b	MYH11	-0.47	8.85e-22	3.28	-11.11	1
hsa-miR-200c	SULF1	-0.47	8.87e-22	-5.41	5.32	1
hsa-miR-17	OSR1	-0.47	9.24e-22	10.38	-5.28	1
hsa-miR-17	SYNE1	-0.47	9.83e-22	10.38	-2.59	1

Table 9: Top 45 miRNA-mRNA pairs(sorted by adjusted p-value) that have: pval-corrected<0.05 and appear at least 1 times in the following databases: microCosm_v5_18, targetScan_v6.2_18.

6 Functional analysis

6.1 Network analysis

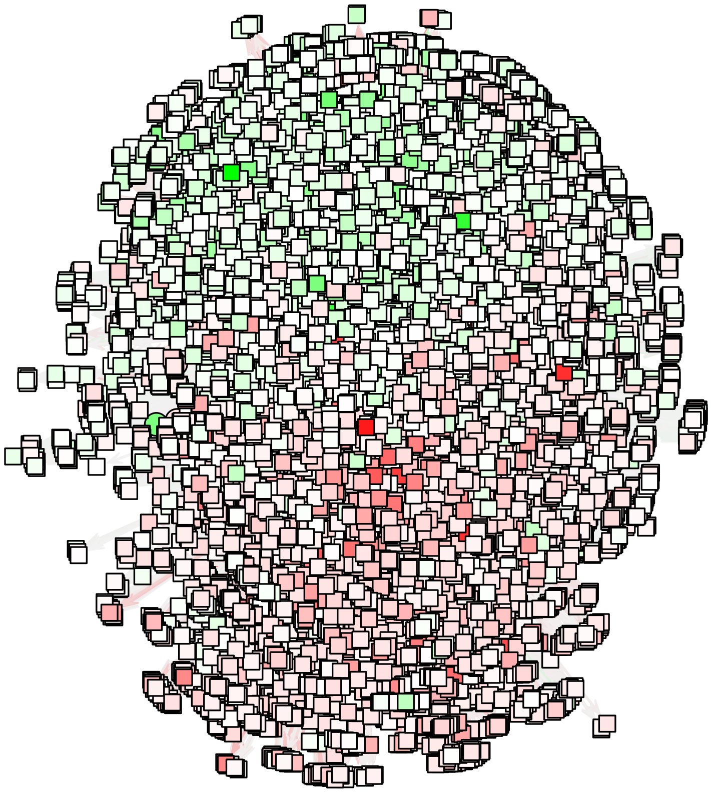

Figure 9: Network for all the miRNA-mRNA pairs that have: pval-corrected<0.05 and appear at least 1 times in the following databases: microCosm_v5_18, targetScan_v6.2_18. Circles represent the miRNAs, and squares the miRNA. Red fill means upregulated miRNAs/mRNAs, while green fill means downregulated mRNA/mRNAs in comparative CvH; lines indicate the miRNA-mRNA pairs, red line means positive score and green line means negative score.

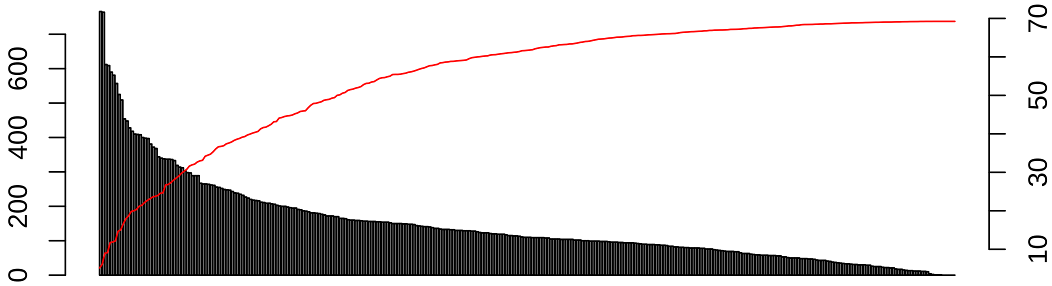

Figure 10: Barplot for miRNAs, pval-corrected<0.05 and Targets=microCosm_v5_18, targetScan_v6.2_18(minimum coincidences between databases:1). Red line (and right axis) represents the percentage of deregulated mRNAs that are targeted by the miRNAs.

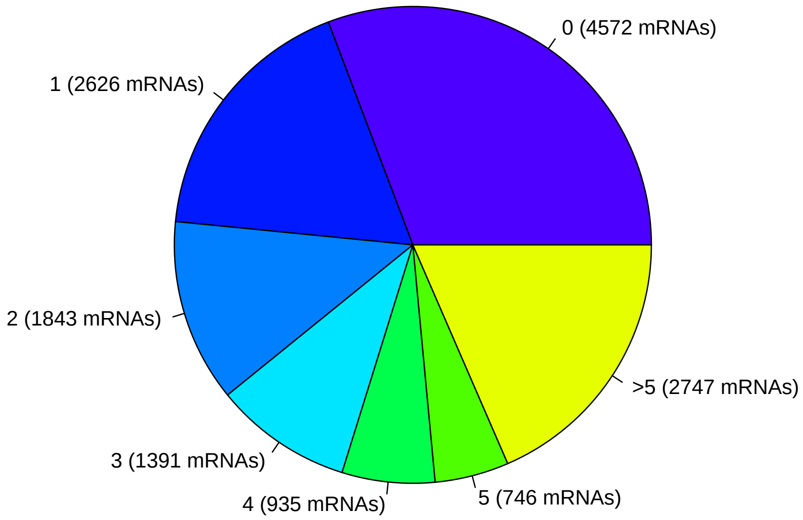

Figure 11: Pie chart representing the number of miRNAs targeting the mRNAs, pval-corrected<0.05 and Targets=microCosm_v5_18, targetScan_v6.2_18(minimum coincidences between databases:1).

miRNA	#targets	cum. %	targets (top 20)
**hsa-miR-106a**	766	5.15	KIAA0247, AKAP13, AHNAK, SH3PXD2A, PTPN21, TMEM127, GOLGA2, LUZP1, ZBTB4, MTMR3, BCL2L2, FZD4, FRMD4B, FYCO1, TRIP11, VCL, ANKRD12, SYNE1, SNRK, NCOR1
**hsa-miR-17**	764	6.1	KIAA0247, AHNAK, TMEM127, FAM129A, ZBTB4, GSN, KCNMA1, KIAA0513, AKAP13, OSR1, SYNE1, FGFR2, PSD, TNS1, PTPN21, ZBTB47, NTN1, TIMP2, ST6GALNAC6, TXNIP
**hsa-miR-19a**	612	8.99	GLTP, PTPN21, SCN9A, MYH11, SYNPO2, C10orf26, PDE5A, CBX7, CILP, PLCL2, FBXO32, TNS1, ZER1, SLC9A1, LPP, PDE7B, SCN4B, BCAR3, NPTN, SCN1B
**hsa-miR-20a**	609	9.12	KIAA0247, FAM129A, KIAA0513, KCNMA1, OSR1, TMEM127, PSD, FGFR2, NTN1, AHNAK, TNS1, SYNE1, PTPN21, FGL2, CNN1, KIAA1683, RELL1, LMO3, ST6GALNAC6, CYBRD1
**hsa-miR-16**	590	11.74	SLC36A1, LMOD1, MYLK, TMEM100, SCN4B, SMAD7, PPAP2A, CNN1, DCLK1, KIAA0247, PPAP2B, ADAMTSL3, MAP1A, PRELP, BCL2L2, CNNM2, SMPD1, IGF1, NFE2L1, DIXDC1
**hsa-miR-106b**	581	11.9	KIAA0247, TMEM127, TMEM100, KCNMA1, SLC36A1, FAM129A, FBLIM1, TNS1, LMO3, KIAA0513, CNN1, CYBRD1, PSD, RGMA, AHNAK, PTPN21, NTN1, SGCA, SYNM, SV2B
**hsa-miR-19b**	557	12.17	GLTP, MYH11, MAF, PLCL2, SLC9A1, NPTN, PTPN21, PDZD4, PDE5A, ABHD5, SYNPO2, PSD, CILP, C10orf26, BCAR3, SGK1, TNS1, CSF1, FBXO32, CLIP4
**hsa-miR-30c**	525	14.68	LOX, CALU, ADAM12, FAP, LIMS1, TPM4, ITGA5, MEX3B, SNAI2, PRR16, CTHRC1, STC1, FRMD6, GJA1, LPPR4, ZNF281, MRAS, ADAMTS3, DCBLD1, CALD1
**hsa-miR-93**	509	15.03	TIMP2, BNC2, CRYAB, CLIP4, SGCA, GPR137B, PSD, RGMA, LMO3, TNS1, TGFB1I1, KCNMA1, JAZF1, CYBRD1, ATP8B2, ZFPM2, PTGER3, FRMD6, GUCY1A3, ZBTB47
**hsa-miR-96**	454	16.65	LMOD1, TNS1, ASB2, MYL9, C20orf194, GSTM5, CRYAB, ADCY5, ITPR1, MAF, CCDC80, BNC2, SCN9A, FILIP1, C10orf54, CD36, NLGN4X, FAM110B, LDB3, NAALADL1

Table 10: Top 10 miRNA with more targets (each miRNA-mRNA pair has pval-corrected<0.05 and appears at least 1 times in the following databases: microCosm_v5_18, targetScan_v6.2_18). MiRNAs in red are upregulated in CvH, miRNAs in green are downregulated in CvH.

mRNA	#miRNAs	miRNAs (top 20)
**QKI**	52	hsa-miR-141, hsa-miR-577, hsa-miR-200a, hsa-miR-200b, hsa-miR-429, hsa-miR-96, hsa-miR-194, hsa-miR-130b, hsa-miR-93, hsa-miR-362-5p, hsa-miR-183, hsa-miR-93*, hsa-miR-200c, hsa-miR-17, hsa-miR-576-5p, hsa-miR-19a, hsa-miR-106b, hsa-miR-30c, hsa-miR-375, hsa-miR-148a
**FOXP2**	49	hsa-miR-7-1*, hsa-miR-128, hsa-miR-7, hsa-miR-203, hsa-miR-186, hsa-miR-200a, hsa-miR-141, hsa-miR-576-5p, hsa-miR-577, hsa-miR-222, hsa-miR-16, hsa-miR-590-3p, hsa-miR-20a*, hsa-miR-19a, hsa-miR-590-5p, hsa-miR-660, hsa-miR-10a*, hsa-miR-29a*, hsa-miR-15a, hsa-miR-130b*
**RORA**	48	hsa-miR-577, hsa-miR-93, hsa-miR-17, hsa-miR-141, hsa-miR-106a, hsa-miR-200a, hsa-miR-345, hsa-miR-106b, hsa-miR-16, hsa-miR-335*, hsa-miR-183, hsa-miR-20a*, hsa-miR-19a, hsa-miR-18a, hsa-miR-590-3p, hsa-miR-3613-5p, hsa-miR-576-5p, hsa-miR-20a, hsa-miR-148a, hsa-miR-19b
**IGF1**	45	hsa-miR-130b, hsa-miR-577, hsa-miR-19a, hsa-miR-16, hsa-miR-576-5p, hsa-miR-19b, hsa-miR-18a, hsa-miR-425, hsa-miR-192, hsa-miR-625, hsa-miR-186, hsa-miR-454, hsa-miR-15a, hsa-miR-335*, hsa-miR-942, hsa-miR-29b, hsa-miR-148a, hsa-miR-301a, hsa-miR-590-3p, hsa-miR-196a
**BNC2**	44	hsa-miR-141, hsa-miR-17, hsa-miR-577, hsa-miR-130b, hsa-miR-93, hsa-miR-429, hsa-miR-200a, hsa-miR-200b, hsa-miR-106b, hsa-miR-191, hsa-miR-20a, hsa-miR-590-5p, hsa-miR-96, hsa-miR-200c, hsa-miR-425, hsa-miR-576-5p, hsa-miR-183, hsa-miR-200c*, hsa-miR-186, hsa-miR-19b
**TNRC6B**	41	hsa-miR-17, hsa-miR-106a, hsa-miR-203, hsa-miR-20a, hsa-miR-362-5p, hsa-miR-590-5p, hsa-miR-335, hsa-miR-19a, hsa-miR-2355-5p, hsa-miR-19b, hsa-miR-18a, hsa-miR-16, hsa-miR-301a, hsa-miR-106b, hsa-miR-135b, hsa-miR-503, hsa-miR-148a, hsa-miR-424, hsa-miR-29b, hsa-miR-369-3p
**LPP**	40	hsa-miR-19a, hsa-miR-18a, hsa-miR-19b, hsa-miR-16, hsa-miR-96, hsa-miR-141, hsa-miR-590-3p, hsa-miR-577, hsa-miR-32, hsa-miR-15a, hsa-miR-425, hsa-miR-424, hsa-miR-203, hsa-miR-628-5p, hsa-miR-576-5p, hsa-miR-30e, hsa-miR-450b-5p, hsa-miR-142-3p, hsa-miR-2355-5p, hsa-miR-26b
**RUNX1T1**	40	hsa-miR-130b, hsa-miR-429, hsa-miR-25, hsa-miR-16, hsa-miR-200c, hsa-miR-15a, hsa-miR-203, hsa-miR-200b, hsa-miR-186, hsa-miR-500a, hsa-miR-192, hsa-miR-19a, hsa-miR-501-3p, hsa-miR-584, hsa-miR-19b, hsa-miR-148a, hsa-miR-29b, hsa-miR-15b, hsa-miR-148b, hsa-miR-215
**TRPS1**	39	hsa-miR-93, hsa-miR-362-5p, hsa-miR-17, hsa-miR-194, hsa-miR-130b, hsa-miR-203, hsa-miR-148a, hsa-miR-19a, hsa-miR-106b, hsa-miR-20a, hsa-miR-200c, hsa-miR-429, hsa-miR-200b, hsa-miR-19b, hsa-miR-186, hsa-miR-106a, hsa-miR-33a, hsa-miR-500b, hsa-miR-3613-5p, hsa-miR-345
**BACH2**	38	hsa-miR-141, hsa-miR-130b, hsa-miR-186, hsa-miR-425, hsa-miR-429, hsa-miR-200a, hsa-miR-200b, hsa-miR-148a, hsa-miR-33a, hsa-miR-454, hsa-miR-301a, hsa-miR-29b, hsa-miR-200c, hsa-miR-16, hsa-miR-183, hsa-miR-15a, hsa-miR-96, hsa-miR-148b, hsa-miR-552, hsa-miR-33b

Table 11: Top 10 mRNA with more miRNAs targeting them (each miRNA-mRNA pair has pval-corrected<0.05 and appears at least 1 times in the following databases: microCosm_v5_18, targetScan_v6.2_18). MRNAs in red are upregulated in CvH, mRNAs in green are downregulated in CvH.

6.2 GO analysis

GOBPID	Term	Count	Size	ExpCount	OddsRatio	fdr	Pvalue
GO:0035556	intracellular signal transduction	1401	2009	1137.75	1.92	1.58e-34	1.39e-38
GO:0048518	positive regulation of biological process	2490	3817	2161.66	1.62	1.18e-32	2.08e-36
GO:0044267	cellular protein metabolic process	2216	3370	1908.51	1.64	2.62e-31	6.95e-35
GO:0044237	cellular metabolic process	5373	8855	5014.80	1.52	3.88e-31	1.37e-34
GO:0048522	positive regulation of cellular process	2224	3397	1923.80	1.62	6.77e-30	3.83e-33
GO:0006464	cellular protein modification process	1733	2583	1462.82	1.71	6.77e-30	4.18e-33
GO:0036211	protein modification process	1733	2583	1462.82	1.71	6.77e-30	4.18e-33
GO:0048583	regulation of response to stimulus	1827	2741	1552.30	1.68	1.57e-29	1.17e-32
GO:0006793	phosphorus metabolic process	1868	2809	1590.81	1.67	1.57e-29	1.28e-32
GO:0006796	phosphate-containing compound metabolic process	1840	2763	1564.75	1.68	1.57e-29	1.39e-32

Table 12: Biological Process . Options used: mRNAs that are present in a mRNA-mRNA pair that has adjusted-pval cutoff <0.05; that also appears at least 1 times (databases: microCosm_v5_18, targetScan_v6.2_18); organism: human.

GOCCID	Term	Count	Size	ExpCount	OddsRatio	fdr	Pvalue
GO:0044424	intracellular part	7302	12258	6561.96	2.53	4.27e-147	3.27e-150
GO:0005622	intracellular	7360	12396	6635.83	2.53	4.63e-144	7.09e-147
GO:0005737	cytoplasm	5814	9342	5000.96	2.23	7.82e-141	1.80e-143
GO:0043227	membrane-bounded organelle	6297	10399	5566.79	2.13	4.86e-119	1.49e-121
GO:0043226	organelle	6690	11215	6003.62	2.13	4.30e-112	1.64e-114
GO:0044444	cytoplasmic part	4390	6864	3674.44	2.06	1.17e-111	5.38e-114
GO:0043231	intracellular membrane-bounded organelle	5829	9551	5112.84	2.04	4.10e-110	2.20e-112
GO:0043229	intracellular organelle	6360	10617	5683.49	2.03	7.45e-104	4.56e-106
GO:0044446	intracellular organelle part	3934	6274	3358.60	1.82	1.53e-74	1.06e-76
GO:0044422	organelle part	4008	6449	3452.28	1.77	8.90e-69	6.82e-71

Table 13: Cellular Component . Options used: mRNAs that are present in a mRNA-mRNA pair that has adjusted-pval cutoff <0.05; that also appears at least 1 times (databases: microCosm_v5_18, targetScan_v6.2_18); organism: human.

GOMFID	Term	Count	Size	ExpCount	OddsRatio	fdr	Pvalue
GO:0005515	protein binding	4960	7904	4344.09	1.94	2.97e-87	8.95e-91
GO:0005488	binding	7046	12075	6636.50	1.97	4.06e-59	2.44e-62
GO:0003824	catalytic activity	3195	5197	2856.31	1.50	9.52e-29	8.60e-32
GO:0019899	enzyme binding	821	1175	645.79	2.00	8.66e-25	1.04e-27
GO:0043168	anion binding	1585	2493	1370.17	1.53	6.65e-19	1.00e-21
GO:0043167	ion binding	3457	5793	3183.87	1.36	1.18e-17	2.13e-20
GO:0032403	protein complex binding	464	656	360.54	2.04	9.22e-15	1.94e-17
GO:0019901	protein kinase binding	288	383	210.50	2.54	3.60e-14	8.67e-17
GO:0019904	protein domain specific binding	383	532	292.39	2.16	5.48e-14	1.61e-16
GO:0097367	carbohydrate derivative binding	1335	2115	1162.42	1.48	5.48e-14	1.65e-16

Table 14: Molecular Function . Options used: mRNAs that are present in a mRNA-mRNA pair that has adjusted-pval cutoff <0.05; that also appears at least 1 times (databases: microCosm_v5_18, targetScan_v6.2_18); organism: human.

KEGGID	Term	Count	Size	ExpCount	OddsRatio	fdr	Pvalue
05200	Pathways in cancer	236	314	183.44	2.24	3.16e-08	1.40e-10
04510	Focal adhesion	150	192	112.17	2.61	4.49e-07	3.98e-09
04142	Lysosome	95	117	68.35	3.13	7.93e-06	1.05e-07
04350	TGF-beta signaling pathway	70	83	48.49	3.89	1.70e-05	3.02e-07
05215	Prostate cancer	72	87	50.83	3.47	4.19e-05	9.28e-07
05220	Chronic myeloid leukemia	61	72	42.06	4.00	4.87e-05	1.29e-06
04380	Osteoclast differentiation	93	118	68.94	2.70	6.18e-05	1.91e-06
05142	Chagas disease (American trypanosomiasis)	81	102	59.59	2.79	1.38e-04	5.15e-06
04666	Fc gamma R-mediated phagocytosis	74	92	53.75	2.97	1.38e-04	5.50e-06
05214	Glioma	53	63	36.80	3.82	1.98e-04	1.02e-05

Table 15: Kegg Pathways . Options used: mRNAs that are present in a mRNA-mRNA pair that has adjusted-pval cutoff <0.05; that also appears at least 1 times (databases: microCosm_v5_18, targetScan_v6.2_18); organism: human.
