# Supplementary material for: MiRComb: An R Package to Analyse miRNA-mRNA Interactions. Examples across Five Digestive Cancers
Source: PLoS One. 2016 Mar 11;11(3):e0151127. doi: 10.1371/journal.pone.0151127 (PMC4788200; doi:10.1371/journal.pone.0151127)
Supplement: S2 File — The report has been made by mkReport function. (PDF) [file pone.0151127.s005.pdf]

Default miRComb output

/home/mvila/Baixades/TCGA/rectum

May 13, 2015

1 Exploratory analysis of miRNA dataset

Number of miRNAs analysed	325
Number of samples	160

Table 1: Basic information of the miRNA dataset.

	group.n	CvH	center	sample	batch	platform
1	NT: 3	Min. :0.0000	AG :71	TCGA-AF-2687-01: 1	Batch 42 :36	mirnas.old:160
2	TP:157	1st Qu.:1.0000	AF :18	TCGA-AF-2689-11: 1	Batch 122:27	mrnas.v1 : 0
3		Median :1.0000	EI :17	TCGA-AF-2690-01: 1	Batch 139:25	mrnas.v2 : 0
4		Mean :0.9812	DC :13	TCGA-AF-2691-01: 1	Batch 46 :17	
5		3rd Qu.:1.0000	F5 :12	TCGA-AF-2691-11: 1	Batch 158:15	
6		Max. :1.0000	AH : 7	TCGA-AF-2692-01: 1	Batch 102:14	
7			(Other):22	(Other) :154	(Other) :26	

Table 2: Summary of the phenotypical information of the miRNA dataset.

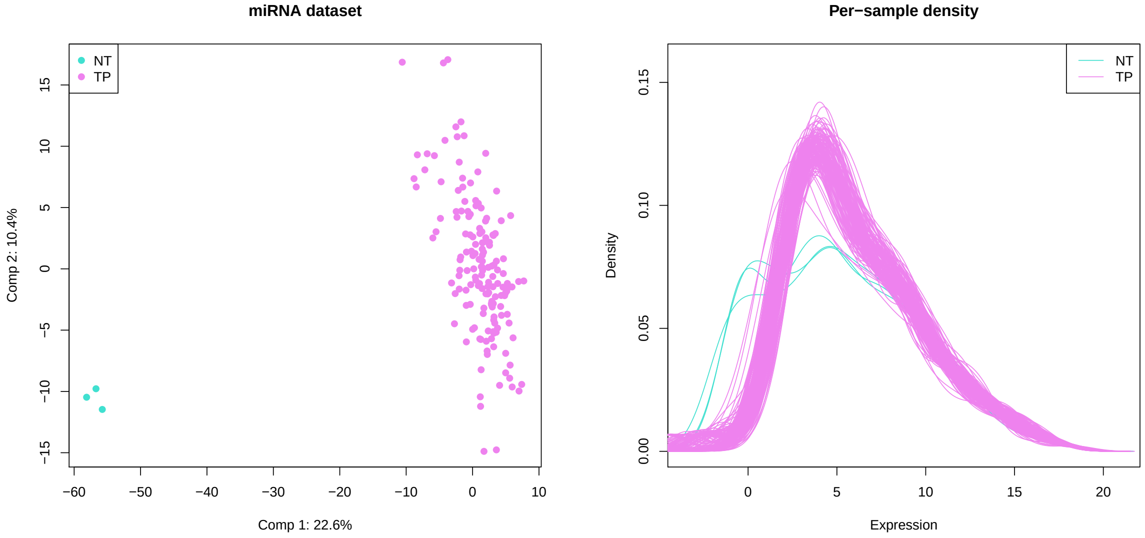

Figure 1: PCA and density plot for miRNAs.

2 Exploratory analysis of mRNA dataset

Number of mRNAs analysed	14973
Number of samples	160

Table 3: Basic information of the mRNA dataset.

	group.n	CvH	center	sample	batch	platform
1	NT: 3	Min. :0.0000	AG :71	TCGA-AF-2687-01: 1	Batch 42 :36	mirnas.old: 0
2	TP:157	1st Qu.:1.0000	AF :18	TCGA-AF-2689-11: 1	Batch 122:27	mrnas.v1 :160
3		Median :1.0000	EI :17	TCGA-AF-2690-01: 1	Batch 139:25	mrnas.v2 : 0
4		Mean :0.9812	DC :13	TCGA-AF-2691-01: 1	Batch 46 :17	
5		3rd Qu.:1.0000	F5 :12	TCGA-AF-2691-11: 1	Batch 158:15	
6		Max. :1.0000	AH : 7	TCGA-AF-2692-01: 1	Batch 102:14	
7			(Other):22	(Other) :154	(Other) :26	

Table 4: Summary of the phenotypical information of the mRNA dataset.

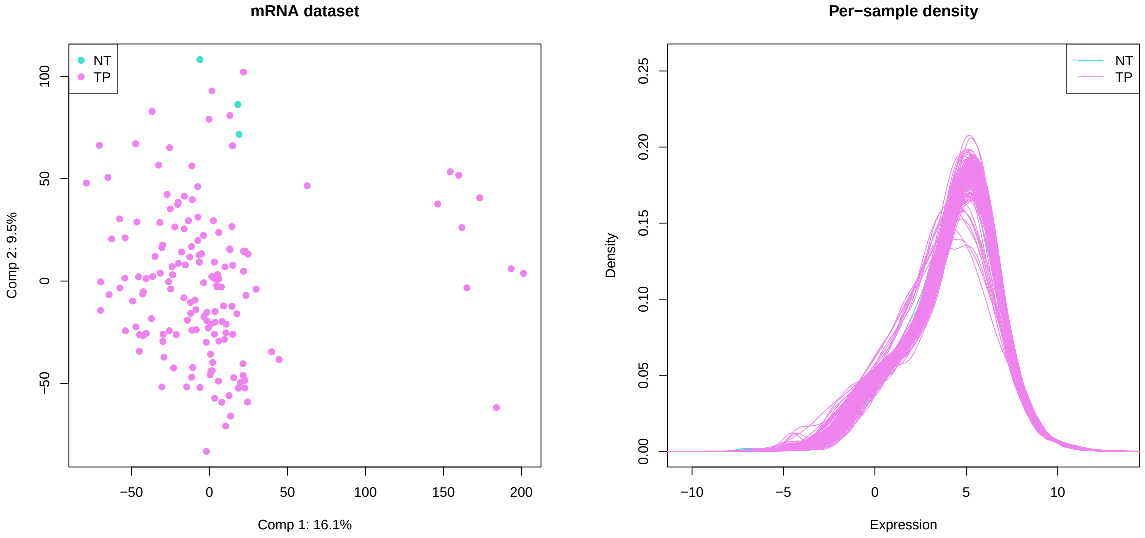

Figure 2: PCA and density plot for mRNAs.

3 Differentially expressed miRNAs

Analysis performed	Comparative used: CvH; method used: limma.
Number of differentially expressed miRNAs	325 (202 upregulated, 123 downregulated)
Number of samples	160
Criteria for selecting miRNAs	adj.pval < 1

Table 5: Basic statistics

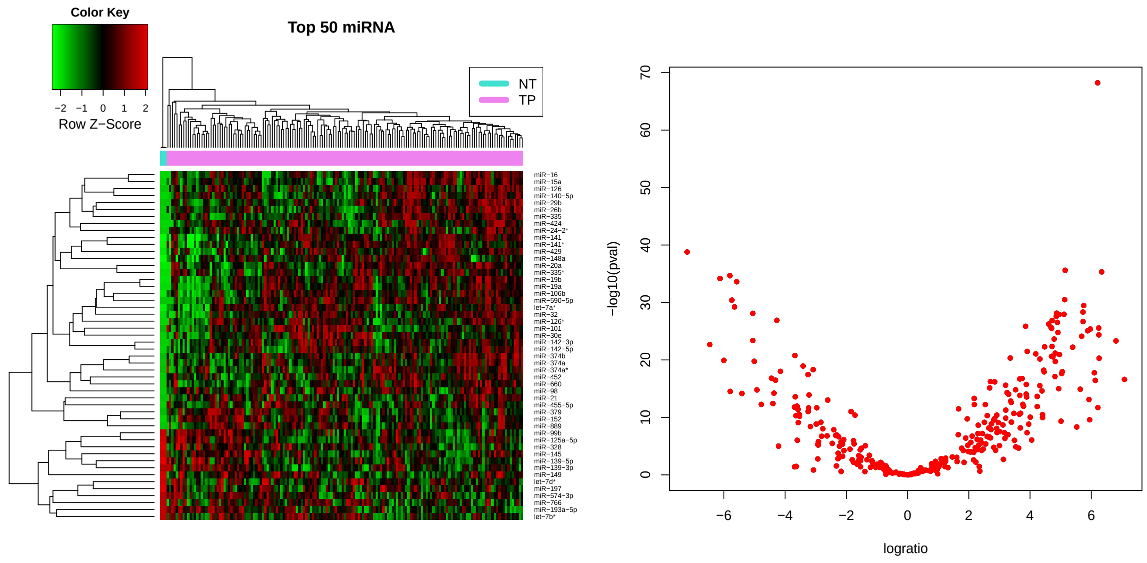

Figure 3: A) Heatmap vith the top 50 most significant miRNAs (sorted by adjusted p-value). B) Volcano plot showing the selected miRNAs.

4 Differentially expressed mRNAs

Analysis performed	Comparative used: CvH; method used: limma.
Number of differentially expressed mRNAs	14973 (8595 upregulated, 6378 downregulated)
Number of samples	160
Criteria for selecting mRNAs	adj.pval < 1

Table 6: Basic statistics

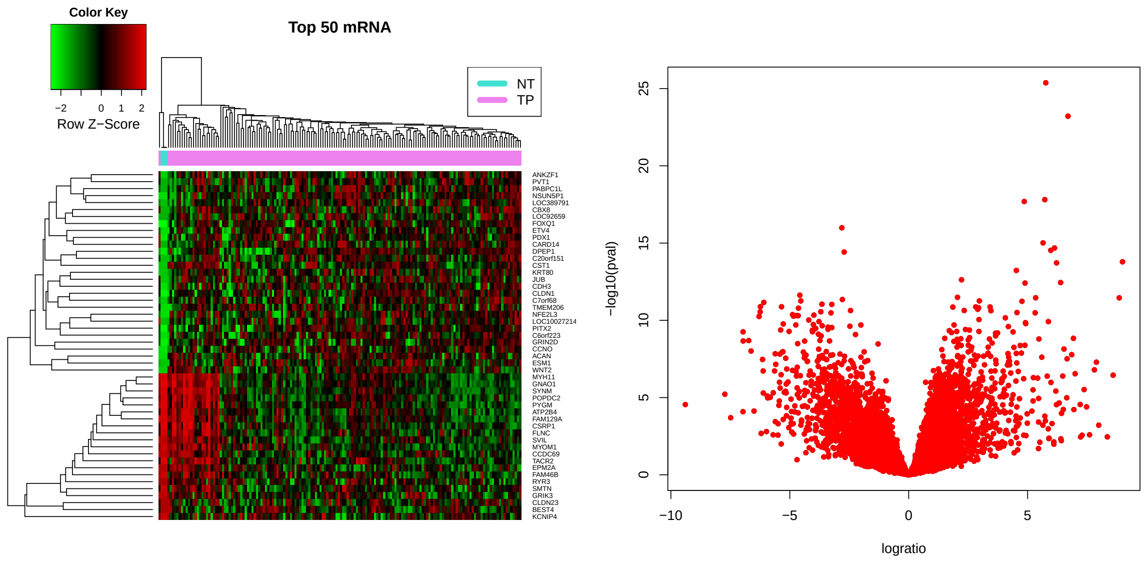

Figure 4: A) Heatmap vith the top 50 most significant mRNAs (sorted by adjusted p-value). B) Volcano plot showing the selected mRNAs.

5 Correlation & intersection with databases

Number of miRNAs	325
Number of mRNAs	14973
Total miRNA-mRNA combinations	4866225
Number of samples	160

Table 7: Number of miRNAs, mRNAs and samples used for correlation.

	Number	%
Total correlations	4866225	100
Total negative correlations	2543118	52.26
Total correlations p<0.05	979376	20.13
Total correlations p<0.01	557475	11.46
Total correlations adj.p<0.05	423296	8.7
Total correlations adj.p<0.01	204266	4.2

Table 8: Basic statistics for correlation results. Correlation hypothesis: two.sided.

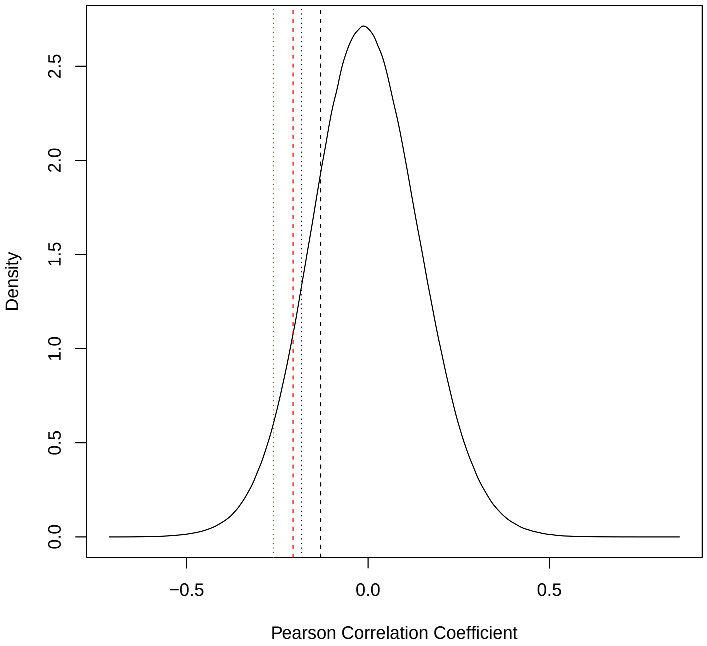

Figure 5: Density of a total of 4866225 miRNA-mRNA pairs. Dashed lines distinguish correlations whose p-value is lower than 0.05, dotted lines for 0.01. Black is for raw p-value and red for adjusted p-value.

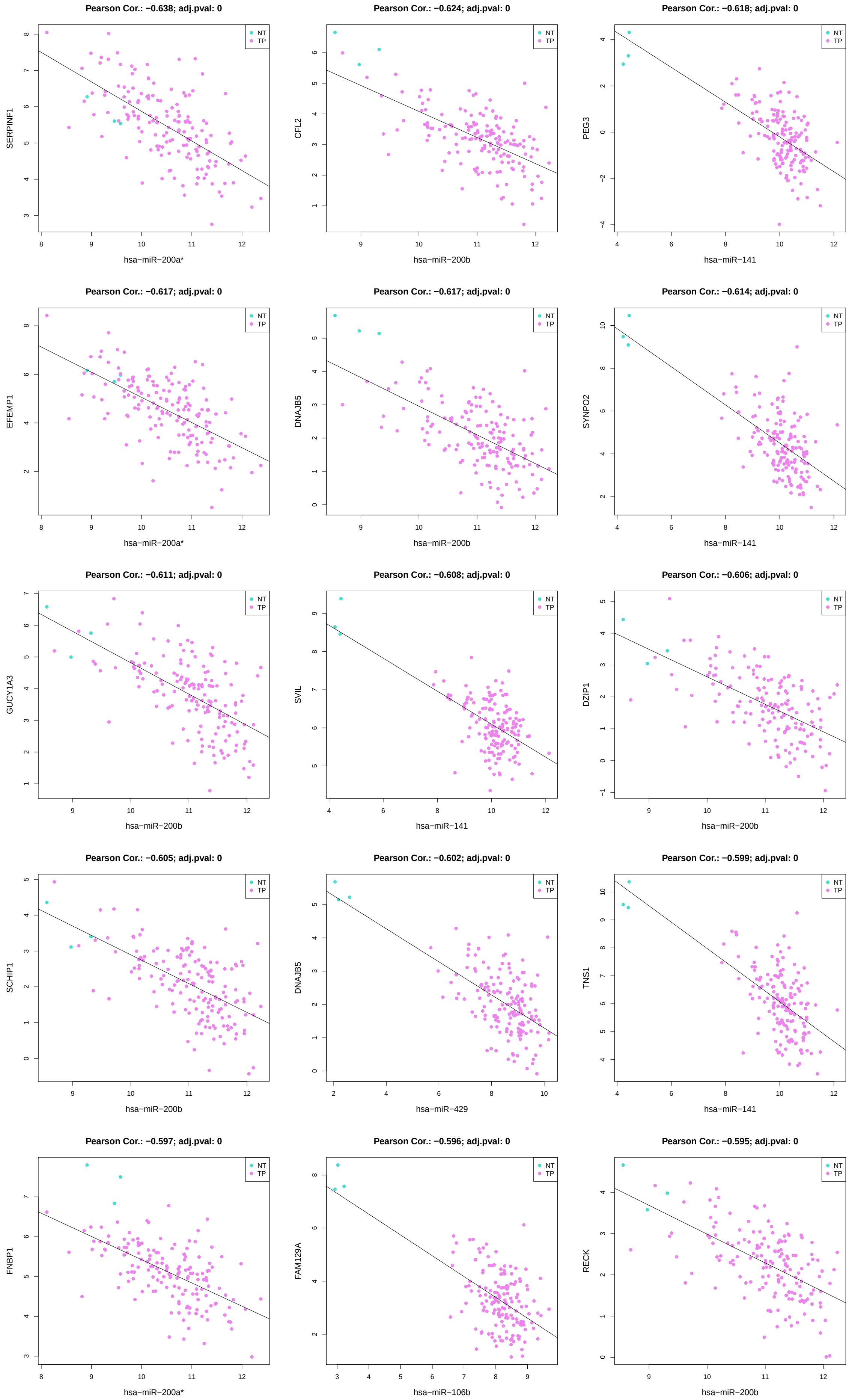

Figure 6: Plot of 15 top correlations, sorted by adjusted p-value. Databases used: microCosm_v5_18, targetScan_v6.2_18 (each miRNA-mRNA pair has to appear at least 1 times).

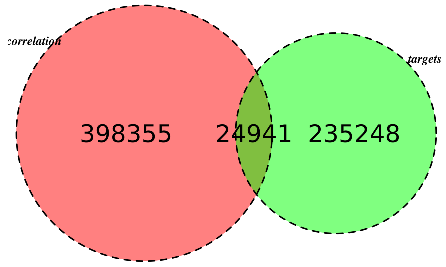

Figure 7: Venn Diagram. Left (red): number of miRNA-mRNA pairs with ajdusted p-value<0.05. Right (green): number of all the theoretical miRNA-mRNA pairs reported at least 1 times in the following databases: microCosm_v5_18, targetScan_v6.2_18. Intersection: miRNA-mRNA pairs that fulfil both conditions.

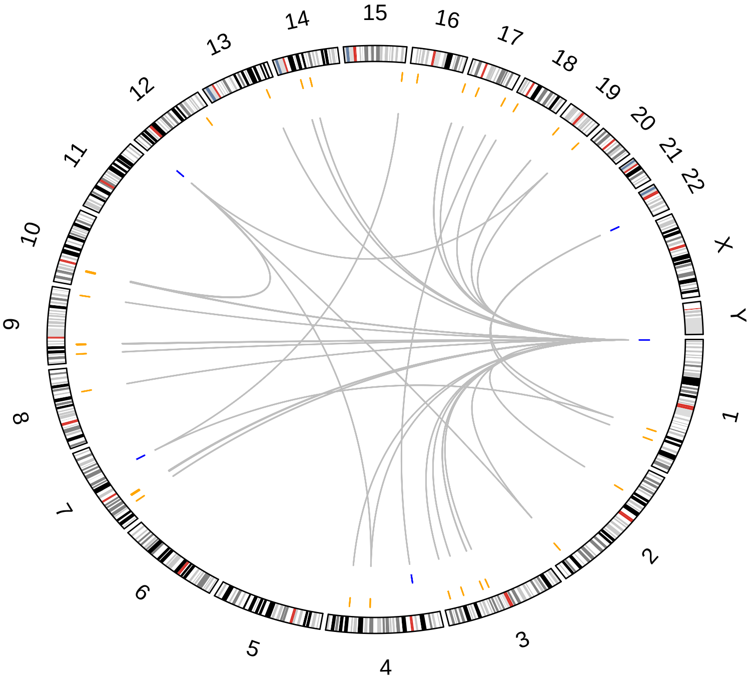

Figure 8: Circos plot for the first 45 miRNA-mRNA pairs (sorted by adjusted p-value) that have: pval-corrected<0.05 and appear at least 1 times in the following databases: microCosm_v5_18, targetScan_v6.2_18. Blue: miRNAs, Orange: target mRNAs

miRNA	mRNA	cor	adj.pval	FC.miRNA	FC.mRNA	dat.sum
hsa-miR-200a*	SERPINF1	-0.64	5.58e-15	2.32	-1.28	1
hsa-miR-200b	CFL2	-0.62	2.93e-14	4.31	-7.81	1
hsa-miR-141	PEG3	-0.62	6.11e-14	53.91	-13.95	1
hsa-miR-200a*	EFEMP1	-0.62	7.11e-14	2.32	-2.72	1
hsa-miR-200b	DNAJB5	-0.62	7.11e-14	4.31	-10.05	1
hsa-miR-141	SYNPO2	-0.61	1.03e-13	53.91	-38.72	1
hsa-miR-200b	GUCY1A3	-0.61	1.36e-13	4.31	-4.01	2
hsa-miR-141	SVIL	-0.61	2.19e-13	53.91	-6.91	1
hsa-miR-200b	DZIP1	-0.61	2.71e-13	4.31	-3.80	1
hsa-miR-200b	SCHIP1	-0.61	2.88e-13	4.31	-2.99	1
hsa-miR-429	DNAJB5	-0.60	4.43e-13	75.95	-10.05	1
hsa-miR-141	TNS1	-0.60	5.68e-13	53.91	-13.80	1
hsa-miR-200a*	FNBP1	-0.60	7.15e-13	2.32	-4.91	1
hsa-miR-106b	FAM129A	-0.60	8.53e-13	35.05	-23.87	1
hsa-miR-200b	RECK	-0.59	9.47e-13	4.31	-3.56	1
hsa-miR-141	ZEB1	-0.59	1.24e-12	53.91	-5.27	1
hsa-miR-200b	RAB34	-0.59	1.25e-12	4.31	-2.05	1
hsa-miR-200a*	AEBP1	-0.59	1.31e-12	2.32	1.03	1
hsa-miR-200b*	SERPINF1	-0.59	1.33e-12	-1.55	-1.28	1
hsa-miR-429	MYLK	-0.59	1.54e-12	75.95	-16.10	1
hsa-miR-200b	BNC2	-0.59	2.62e-12	4.31	-6.84	1
hsa-miR-200a	ZEB1	-0.58	2.91e-12	21.99	-5.27	1
hsa-miR-574-3p	CBX8	-0.58	2.91e-12	-50.33	4.66	1
hsa-miR-200b	ZEB1	-0.58	3.22e-12	4.31	-5.27	2
hsa-miR-200a	JAZF1	-0.58	3.23e-12	21.99	-4.46	1
hsa-miR-200a	PEG3	-0.58	3.48e-12	21.99	-13.95	1
hsa-miR-429	KANK2	-0.58	3.71e-12	75.95	-5.55	1
hsa-miR-200b	KIAA1462	-0.58	4.07e-12	4.31	-6.87	1
hsa-miR-200b	MYLK	-0.58	4.31e-12	4.31	-16.10	1
hsa-miR-200b	JAZF1	-0.58	4.48e-12	4.31	-4.46	1
hsa-let-7a*	MYH11	-0.58	4.89e-12	18.21	-78.36	1
hsa-miR-33a	FAM129A	-0.58	4.93e-12	68.60	-23.87	1
hsa-miR-200b	TUBB6	-0.58	5.01e-12	4.31	-3.01	1
hsa-miR-200b	FRMD6	-0.58	5.78e-12	4.31	-1.96	1
hsa-miR-200a	CCDC80	-0.58	6.46e-12	21.99	-6.02	1
hsa-miR-200a	TNS1	-0.58	7.23e-12	21.99	-13.80	1
hsa-miR-200a*	CHRD	-0.58	7.73e-12	2.32	-1.69	1
hsa-let-7a*	HSPB8	-0.58	7.74e-12	18.21	-26.84	1
hsa-miR-200b	SDC2	-0.58	8.20e-12	4.31	-2.20	1
hsa-miR-200b	KANK2	-0.57	8.77e-12	4.31	-5.55	1
hsa-miR-33a	ATP2B4	-0.57	9.98e-12	68.60	-9.40	1
hsa-miR-429	CFL2	-0.57	1.18e-11	75.95	-7.81	1
hsa-miR-200b	GLI3	-0.57	1.35e-11	4.31	-7.82	2
hsa-miR-200a	SYNPO2	-0.57	1.56e-11	21.99	-38.72	1
hsa-miR-106b	SYNM	-0.57	1.67e-11	35.05	-68.22	1

Table 9: Top 45 miRNA-mRNA pairs(sorted by adjusted p-value) that have: pval-corrected<0.05 and appear at least 1 times in the following databases: microCosm_v5_18, targetScan_v6.2_18.

6 Functional analysis

6.1 Network analysis

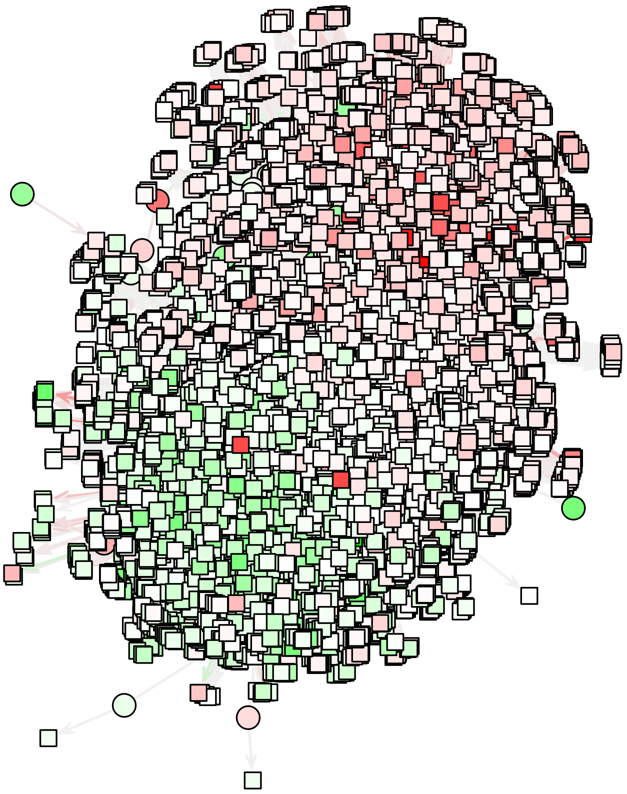

Figure 9: Network for all the miRNA-mRNA pairs that have: pval-corrected<0.05 and appear at least 1 times in the following databases: microCosm_v5_18, targetScan_v6.2_18. Circles represent the miRNAs, and squares the miRNA. Red fill means upregulated miRNAs/mRNAs, while green fill means downregulated mRNA/mRNAs in comparative CvH; lines indicate the miRNA-mRNA pairs, red line means positive score and green line means negative score.

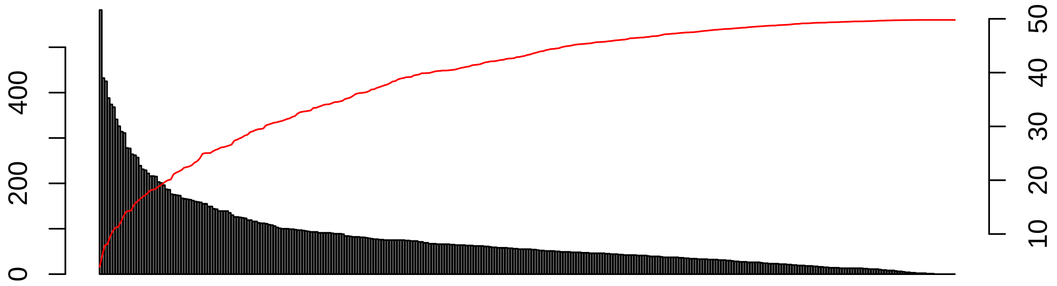

Figure 10: Barplot for miRNAs, pval-corrected<0.05 and Targets=microCosm_v5_18, targetScan_v6.2_18(minimum coincidences between databases:1). Red line (and right axis) represents the percentage of deregulated mRNAs that are targeted by the miRNAs.

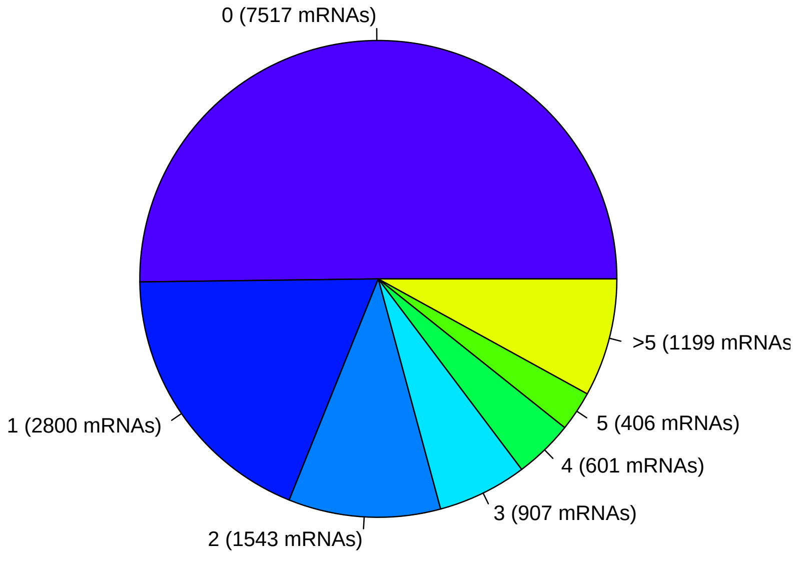

Figure 11: Pie chart representing the number of miRNAs targeting the mRNAs, pval-corrected<0.05 and Targets=microCosm_v5_18, targetScan_v6.2_18(minimum coincidences between databases:1).

miRNA	#targets	cum. %	targets (top 20)
**hsa-miR-106a**	582	3.89	AHNAK, BMPR2, CYLD, PLSCR4, OSTM1, XYLT1, PDLIM5, ANKRD12, PGM2L1, TCF4, HEG1, ZBTB4, FCHO2, ATXN1, RAB8B, KAT2B, AFF1, TAOK3, SNRK, C4orf34
**hsa-miR-19a**	432	6	SYNPO2, RNF11, ITPR1, PDE5A, NPTN, SYNM, CLIP4, MYH11, ITPKB, JAZF1, CXCL12, PRICKLE2, TNS1, SLC8A1, KIAA1462, MBNL1, FAM46B, GLTP, JAM2, IL6ST
**hsa-miR-23a**	425	7.89	WASL, NCOA1, MARCKS, MYO6, UBE2D1, RAP1A, SOS1, MTM1, ATP2B4, EIF4E3, CAB39, SMC5, NEGR1, WIPF2, JAZF1, BBX, RSBN1L, PBX1, CASD1, STX12
**hsa-miR-19b**	388	8.13	SYNPO2, NPTN, SYNM, FAM46B, CLIP4, MYH11, ITPR1, PDE5A, RNF11, CXCL12, GLTP, PRICKLE2, PDE7B, ITPKB, JAZF1, SLC8A1, MEF2C, KIAA1462, C20orf194, TNS1
**hsa-miR-369-3p**	374	9.54	NCOA1, AKAP6, MBNL1, DIXDC1, PLEKHM3, ITPR1, ARHGAP5, HLF, MKL2, ANKRD12, PRKAB2, ABI3BP, C5orf41, TMEM131, AFF1, CREB1, BMPR2, SASH1, RALGPS2, SORBS1
**hsa-miR-30e**	368	10.55	AHNAK, AMOTL1, LPP, LIFR, NEGR1, NECAB1, MYH11, CALD1, TEAD1, GNAO1, KLF9, MBNL1, CFL2, SAMD4A, GNG2, NRP2, PDE5A, NCS1, FYCO1, ITGA5
**hsa-miR-17**	341	11.18	FAM129A, KCNMA1, MYLK, CLIP4, GSN, TMEM100, BAALC, ITPKB, C4orf34, ST6GALNAC6, SYNM, CNN1, PSD, SYNE1, CFL2, ATP1A2, FBXL22, FGL2, BNC2, SPG20
**hsa-miR-106b**	326	11.29	FAM129A, SYNM, ATP1A2, TNS1, CNN1, LMO3, AHNAK, ITPKB, KCNMA1, TMEM100, CLIP4, PSD, BAALC, CFL2, JAZF1, BVES, KIAA1462, CALD1, HSD17B6, ST6GALNAC6
**hsa-miR-27a**	314	12.15	GRIK3, SDPR, LIFR, ADAMTSL3, AFF3, ABCA8, RBPMS2, FNBP1, NCOA1, ATP1A2, SFRP1, PRICKLE2, SPARCL1, SORBS1, PHLPP2, RCAN2, MYOCD, MEF2C, EGFR, VAT1L
**hsa-miR-200b**	311	13.24	CFL2, DNAJB5, GUCY1A3, DZIP1, SCHIP1, RECK, RAB34, BNC2, ZEB1, KIAA1462, MYLK, JAZF1, TUBB6, FRMD6, SDC2, KANK2, GLI3, TIMP2, SGCE, NR3C1

Table 10: Top 10 miRNA with more targets (each miRNA-mRNA pair has pval-corrected<0.05 and appears at least 1 times in the following databases: microCosm_v5_18, targetScan_v6.2_18). MiRNAs in red are upregulated in CvH, miRNAs in green are downregulated in CvH.

mRNA	#miRNAs	miRNAs (top 20)
**QKI**	43	hsa-miR-200b, hsa-miR-200a, hsa-miR-429, hsa-miR-141, hsa-miR-33b, hsa-miR-33a, hsa-miR-106a, hsa-miR-577, hsa-miR-93*, hsa-miR-19a, hsa-miR-106b, hsa-miR-30e, hsa-miR-362-5p, hsa-miR-19b, hsa-miR-27a, hsa-miR-130b, hsa-miR-135b, hsa-miR-17, hsa-miR-576-5p, hsa-miR-345
**BNC2**	38	hsa-miR-200b, hsa-miR-200a, hsa-miR-141, hsa-miR-7-1*, hsa-miR-429, hsa-miR-200c*, hsa-miR-577, hsa-miR-106b, hsa-miR-19a, hsa-miR-17, hsa-miR-19b, hsa-miR-590-5p, hsa-miR-130b, hsa-miR-20a, hsa-miR-576-5p, hsa-miR-30e, hsa-miR-532-5p, hsa-miR-96, hsa-miR-552, hsa-miR-106a
**KCNMA1**	37	hsa-miR-17, hsa-miR-20a, hsa-miR-335*, hsa-miR-106b, hsa-miR-29a*, hsa-miR-20a*, hsa-miR-29b, hsa-miR-450b-5p, hsa-miR-135b, hsa-miR-224, hsa-miR-33a, hsa-miR-93, hsa-miR-576-5p, hsa-miR-16-2*, hsa-miR-584, hsa-miR-942, hsa-miR-29a, hsa-miR-629, hsa-miR-382, hsa-miR-21*
**NRP2**	32	hsa-miR-200b, hsa-miR-200a, hsa-miR-141, hsa-miR-429, hsa-miR-30e, hsa-miR-19a, hsa-miR-106b, hsa-miR-577, hsa-miR-19b, hsa-miR-17, hsa-miR-532-5p, hsa-miR-130b, hsa-miR-942, hsa-miR-146a, hsa-miR-106a, hsa-miR-188-5p, hsa-let-7g*, hsa-miR-20a, hsa-miR-16, hsa-miR-425*
**MBNL1**	31	hsa-miR-369-3p, hsa-miR-19a, hsa-miR-590-5p, hsa-miR-30e, hsa-miR-19b, hsa-miR-18a, hsa-miR-199b-3p, hsa-miR-199a-3p, hsa-miR-130b, hsa-miR-889, hsa-miR-590-3p, hsa-miR-203, hsa-miR-141, hsa-miR-21, hsa-miR-301a, hsa-miR-450b-5p, hsa-miR-135b, hsa-miR-382, hsa-miR-223, hsa-miR-552
**PDE4D**	31	hsa-miR-18a, hsa-miR-552, hsa-miR-130b, hsa-miR-369-3p, hsa-miR-203, hsa-miR-30e, hsa-miR-592, hsa-miR-33a, hsa-miR-493, hsa-miR-374b, hsa-miR-194, hsa-miR-452, hsa-miR-374a, hsa-miR-25, hsa-miR-409-5p, hsa-miR-454, hsa-miR-339-3p, hsa-miR-130a, hsa-miR-30b, hsa-miR-32
**TSHZ3**	30	hsa-miR-200b, hsa-miR-200a, hsa-miR-576-5p, hsa-miR-577, hsa-miR-429, hsa-miR-141, hsa-miR-19a, hsa-miR-17, hsa-miR-18a, hsa-miR-106b, hsa-let-7a*, hsa-miR-19b, hsa-miR-20a, hsa-miR-194, hsa-miR-148a*, hsa-miR-20a*, hsa-miR-335, hsa-miR-590-3p, hsa-miR-106a, hsa-miR-93
**HLF**	29	hsa-miR-369-3p, hsa-miR-106b, hsa-miR-19a, hsa-miR-429, hsa-miR-141, hsa-miR-19b, hsa-miR-200a, hsa-miR-590-3p, hsa-miR-30e, hsa-miR-374a, hsa-miR-337-3p, hsa-miR-130b, hsa-miR-20a, hsa-miR-152, hsa-miR-200b, hsa-miR-18a, hsa-miR-29b, hsa-miR-17, hsa-miR-183, hsa-miR-148a
**IGF1**	29	hsa-miR-18a, hsa-miR-625, hsa-miR-130b, hsa-miR-19a, hsa-miR-26a-2*, hsa-miR-27a, hsa-miR-19b, hsa-miR-577, hsa-miR-16, hsa-miR-192, hsa-miR-590-3p, hsa-miR-629, hsa-miR-24-2*, hsa-miR-30b, hsa-miR-30e, hsa-miR-15a, hsa-miR-222, hsa-miR-215, hsa-miR-362-5p, hsa-miR-148a
**ITPR1**	29	hsa-miR-19a, hsa-miR-19b, hsa-let-7a*, hsa-miR-130b, hsa-miR-203, hsa-miR-429, hsa-miR-369-3p, hsa-miR-200b, hsa-miR-32, hsa-miR-22*, hsa-miR-576-5p, hsa-miR-96, hsa-miR-126, hsa-miR-301a, hsa-miR-424, hsa-miR-136*, hsa-miR-192*, hsa-miR-628-5p, hsa-miR-26b, hsa-miR-450a

Table 11: Top 10 mRNA with more miRNAs targeting them (each miRNA-mRNA pair has pval-corrected<0.05 and appears at least 1 times in the following databases: microCosm_v5_18, targetScan_v6.2_18). MRNAs in red are upregulated in CvH, mRNAs in green are downregulated in CvH.

6.2 GO analysis

GOBPID	Term	Count	Size	ExpCount	OddsRatio	fdr	Pvalue
GO:0050794	regulation of cellular process	3615	7941	3259.02	1.50	1.33e-29	1.28e-33
GO:0050789	regulation of biological process	3784	8398	3446.57	1.48	3.72e-27	7.15e-31
GO:0065007	biological regulation	3982	8895	3650.54	1.49	4.24e-27	1.22e-30
GO:0048518	positive regulation of biological process	1860	3817	1566.51	1.53	7.68e-26	2.95e-29
GO:0035556	intracellular signal transduction	1055	2009	824.50	1.71	7.83e-26	3.76e-29
GO:0048522	positive regulation of cellular process	1673	3397	1394.14	1.55	2.47e-25	1.43e-28
GO:0016043	cellular component organization	2200	4619	1895.66	1.48	4.56e-25	3.11e-28
GO:0051716	cellular response to stimulus	2487	5295	2173.09	1.47	4.56e-25	3.62e-28
GO:0006464	cellular protein modification process	1310	2583	1060.07	1.61	4.56e-25	4.38e-28
GO:0036211	protein modification process	1310	2583	1060.07	1.61	4.56e-25	4.38e-28

Table 12: Biological Process . Options used: mRNAs that are present in a mRNA-mRNA pair that has adjusted-pval cutoff <0.05; that also appears at least 1 times (databases: microCosm_v5_18, targetScan_v6.2_18); organism: human.

GOCCID	Term	Count	Size	ExpCount	OddsRatio	fdr	Pvalue
GO:0044424	intracellular part	5256	12258	4750.75	2.00	1.98e-73	1.61e-76
GO:0005622	intracellular	5296	12396	4804.23	1.99	2.50e-71	4.05e-74
GO:0005737	cytoplasm	4174	9342	3620.61	1.78	1.83e-68	4.47e-71
GO:0043227	membrane-bounded organelle	4524	10399	4030.27	1.72	1.64e-57	5.34e-60
GO:0043226	organelle	4815	11215	4346.52	1.74	1.58e-55	6.42e-58
GO:0043231	intracellular membrane-bounded organelle	4183	9551	3701.61	1.66	2.68e-52	1.31e-54
GO:0043229	intracellular organelle	4573	10617	4114.76	1.67	1.85e-50	1.05e-52
GO:0044444	cytoplasmic part	3109	6864	2660.23	1.59	1.83e-45	1.19e-47
GO:0005829	cytosol	1262	2545	986.35	1.69	1.52e-31	1.11e-33
GO:0044446	intracellular organelle part	2795	6274	2431.57	1.47	9.55e-31	7.76e-33

Table 13: Cellular Component . Options used: mRNAs that are present in a mRNA-mRNA pair that has adjusted-pval cutoff <0.05; that also appears at least 1 times (databases: microCosm_v5_18, targetScan_v6.2_18); organism: human.

GOMFID	Term	Count	Size	ExpCount	OddsRatio	fdr	Pvalue
GO:0005515	protein binding	3683	7904	3143.00	1.83	2.95e-69	1.03e-72
GO:0005488	binding	5151	12075	4801.59	1.88	5.87e-46	4.10e-49
GO:0019899	enzyme binding	616	1175	467.24	1.75	4.24e-17	4.44e-20
GO:0043167	ion binding	2538	5793	2303.57	1.31	5.20e-13	7.25e-16
GO:0004672	protein kinase activity	315	565	224.67	1.96	2.25e-12	3.92e-15
GO:0008092	cytoskeletal protein binding	376	704	279.94	1.79	2.27e-11	4.75e-14
GO:0019904	protein domain specific binding	295	532	211.55	1.93	3.09e-11	7.54e-14
GO:0016773	phosphotransferase activity, alcohol group as acceptor	361	679	270.00	1.77	1.28e-10	3.58e-13
GO:0043168	anion binding	1149	2493	991.33	1.36	4.17e-10	1.31e-12
GO:0016301	kinase activity	380	732	291.08	1.68	1.84e-09	6.42e-12

Table 14: Molecular Function . Options used: mRNAs that are present in a mRNA-mRNA pair that has adjusted-pval cutoff <0.05; that also appears at least 1 times (databases: microCosm_v5_18, targetScan_v6.2_18); organism: human.

KEGGID	Term	Count	Size	ExpCount	OddsRatio	fdr	Pvalue
05200	Pathways in cancer	182	314	129.57	2.04	1.47e-07	6.50e-10
04510	Focal adhesion	118	192	79.23	2.34	8.84e-07	7.83e-09
04360	Axon guidance	78	125	51.58	2.41	8.66e-05	1.19e-06
04144	Endocytosis	113	196	80.88	1.99	8.66e-05	1.87e-06
04514	Cell adhesion molecules (CAMs)	79	128	52.82	2.34	8.66e-05	1.92e-06
04142	Lysosome	70	117	48.28	2.15	1.25e-03	3.32e-05
04810	Regulation of actin cytoskeleton	111	203	83.76	1.75	1.74e-03	5.96e-05
04350	TGF-beta signaling pathway	52	83	34.25	2.42	1.74e-03	6.17e-05
05211	Renal cell carcinoma	43	66	27.23	2.69	1.76e-03	7.04e-05
04520	Adherens junction	45	70	28.88	2.59	1.76e-03	7.80e-05

Table 15: Kegg Pathways . Options used: mRNAs that are present in a mRNA-mRNA pair that has adjusted-pval cutoff <0.05; that also appears at least 1 times (databases: microCosm_v5_18, targetScan_v6.2_18); organism: human.
