# Supplementary material for: MiRComb: An R Package to Analyse miRNA-mRNA Interactions. Examples across Five Digestive Cancers
Source: PLoS One. 2016 Mar 11;11(3):e0151127. doi: 10.1371/journal.pone.0151127 (PMC4788200; doi:10.1371/journal.pone.0151127)
Supplement: S3 File — The report has been made by mkReport function. (PDF) [file pone.0151127.s006.pdf]

Default `miRComb` output

/home/mvila/Baixades/TCGA/esophagus

May 13, 2015

1 Exploratory analysis of miRNA dataset

Number of miRNAs analysed	338
Number of samples	191

Table 1: Basic information of the miRNA dataset.

	group.n	CvH	center	sample	batch
1	NT: 13	Min. :0.0000	L5 :56	TCGA-2H-A9GF-01: 1	Batch 382 :39
2	TP:178	1st Qu.:1.0000	LN :37	TCGA-2H-A9GG-01: 1	Batch 272 :37
3		Median :1.0000	IG :20	TCGA-2H-A9GH-01: 1	Batch 374 :22
4		Mean :0.9319	VR :16	TCGA-2H-A9GI-01: 1	Batch 254 :21
5		3rd Qu.:1.0000	JY :13	TCGA-2H-A9GJ-01: 1	Batch 339 :20
6		Max. :1.0000	R6 :13	TCGA-2H-A9GK-01: 1	Batch 298 :12
7			(Other):36	(Other) :185	(Other) :40

Table 2: Summary of the phenotypical information of the miRNA dataset.

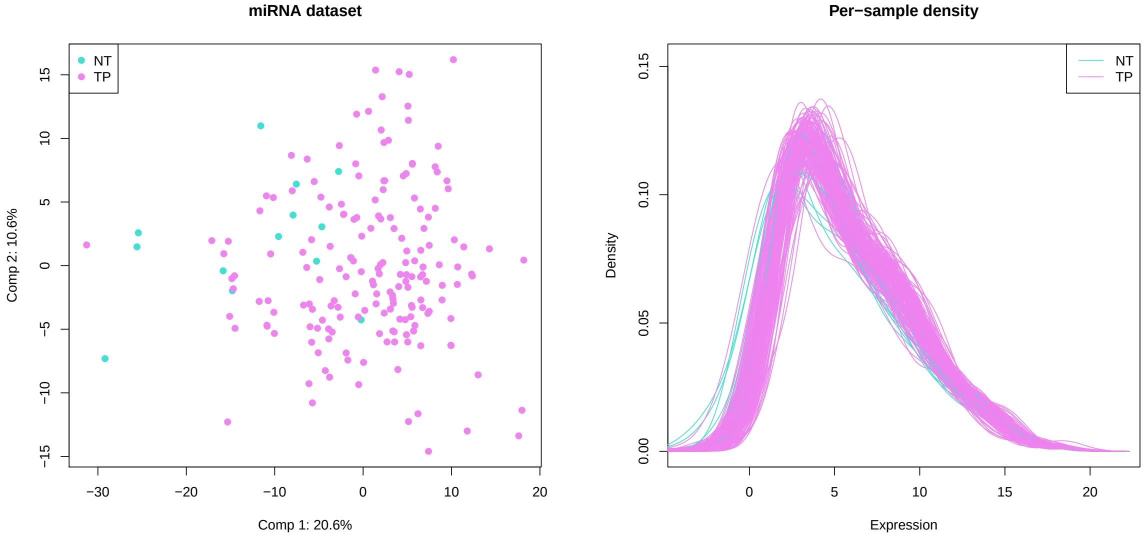

Figure 1: PCA and density plot for miRNAs.

2 Exploratory analysis of mRNA dataset

Number of mRNAs analysed	18807
Number of samples	191

Table 3: Basic information of the mRNA dataset.

	group.n	CvH	center	sample	batch
1	NT: 13	Min. :0.0000	L5 :56	TCGA-2H-A9GF-01: 1	Batch 382 :39
2	TP:178	1st Qu.:1.0000	LN :37	TCGA-2H-A9GG-01: 1	Batch 272 :37
3		Median :1.0000	IG :20	TCGA-2H-A9GH-01: 1	Batch 374 :22
4		Mean :0.9319	VR :16	TCGA-2H-A9GI-01: 1	Batch 254 :21
5		3rd Qu.:1.0000	JY :13	TCGA-2H-A9GJ-01: 1	Batch 339 :20
6		Max. :1.0000	R6 :13	TCGA-2H-A9GK-01: 1	Batch 298 :12
7			(Other):36	(Other) :185	(Other) :40

Table 4: Summary of the phenotypical information of the mRNA dataset.

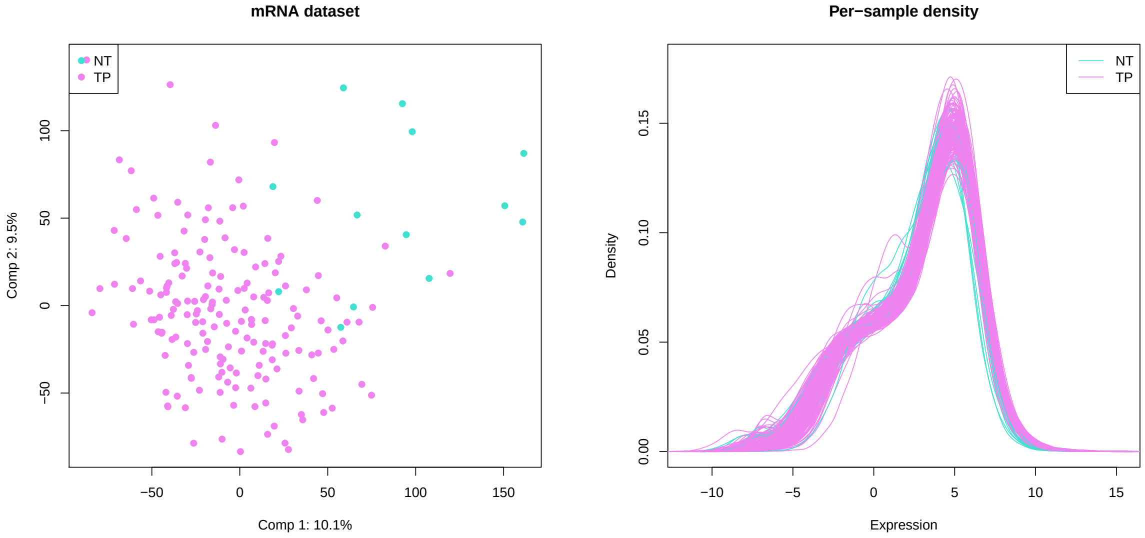

Figure 2: PCA and density plot for mRNAs.

3 Differentially expressed miRNAs

Analysis performed	Comparative used: CvH; method used: limma.
Number of differentially expressed miRNAs	338 (247 upregulated, 91 downregulated)
Number of samples	191
Criteria for selecting miRNAs	adj.pval < 1

Table 5: Basic statistics

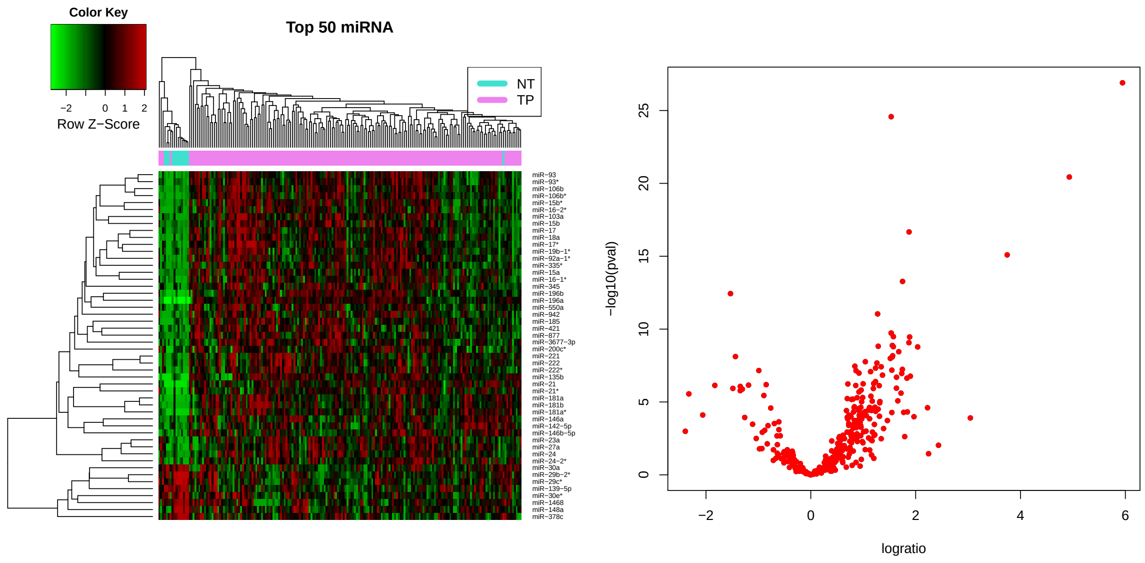

Figure 3: A) Heatmap vith the top 50 most significant miRNAs (sorted by adjusted p-value). B) Volcano plot showing the selected miRNAs.

4 Differentially expressed mRNAs

Analysis performed	Comparative used: CvH; method used: limma.
Number of differentially expressed mRNAs	18807 (13153 upregulated, 5654 downregulated)
Number of samples	191
Criteria for selecting mRNAs	adj.pval < 1

Table 6: Basic statistics

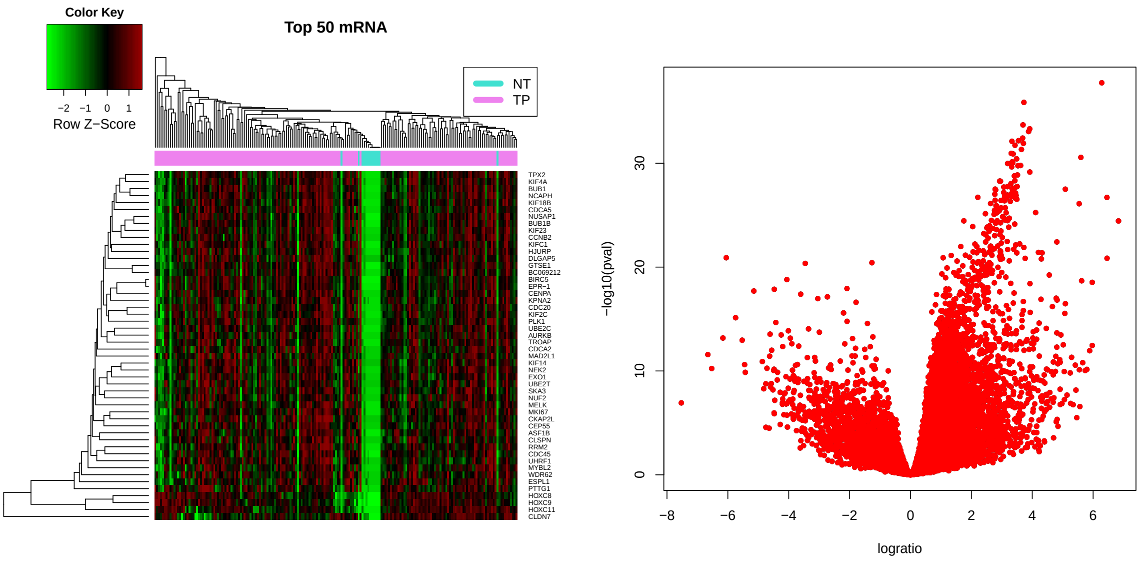

Figure 4: A) Heatmap vith the top 50 most significant mRNAs (sorted by adjusted p-value). B) Volcano plot showing the selected mRNAs.

5 Correlation & intersection with databases

Number of miRNAs	338
Number of mRNAs	18807
Total miRNA-mRNA combinations	6356766
Number of samples	191

Table 7: Number of miRNAs, mRNAs and samples used for correlation.

	Number	%
Total correlations	6356766	100
Total negative correlations	2792875	43.94
Total correlations p<0.05	1138006	17.9
Total correlations p<0.01	705933	11.11
Total correlations adj.p<0.05	568914	8.95
Total correlations adj.p<0.01	336673	5.3

Table 8: Basic statistics for correlation results. Correlation hypothesis: two.sided.

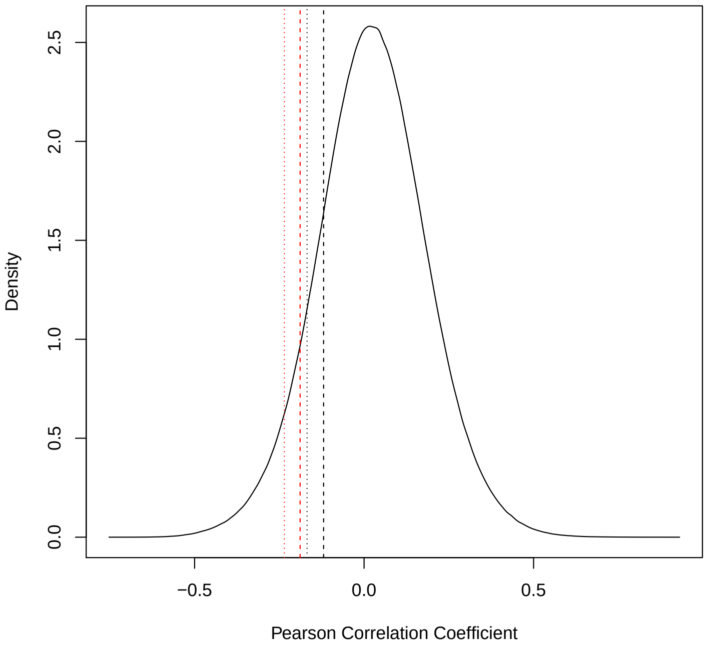

Figure 5: Density of a total of 6356766 miRNA-mRNA pairs. Dashed lines distinguish correlations whose p-value is lower than 0.05, dotted lines for 0.01. Black is for raw p-value and red for adjusted p-value.

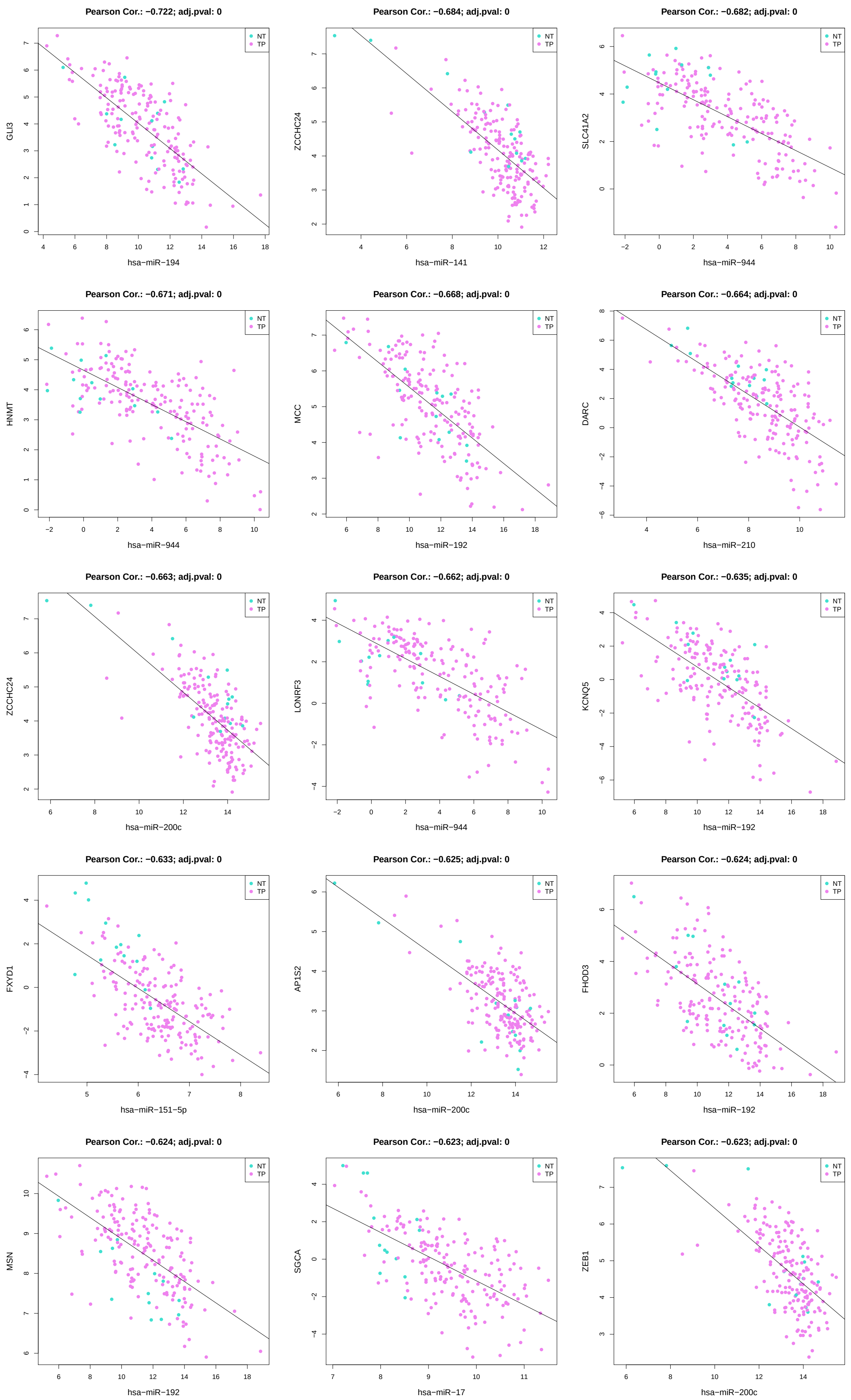

Figure 6: Plot of 15 top correlations, sorted by adjusted p-value. Databases used: microCosm_v5_18, targetScan_v6.2_18 (each miRNA-mRNA pair has to appear at least 1 times).

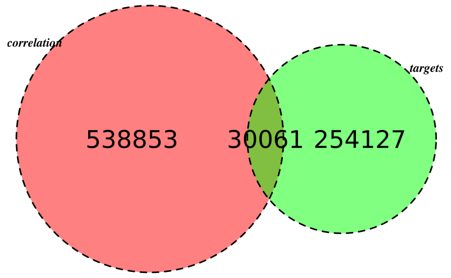

Figure 7: Venn Diagram. Left (red): number of miRNA-mRNA pairs with ajdusted p-value<0.05. Right (green): number of all the theoretical miRNA-mRNA pairs reported at least 1 times in the following databases: microCosm_v5_18, targetScan_v6.2_18. Intersection: miRNA-mRNA pairs that fulfil both conditions.

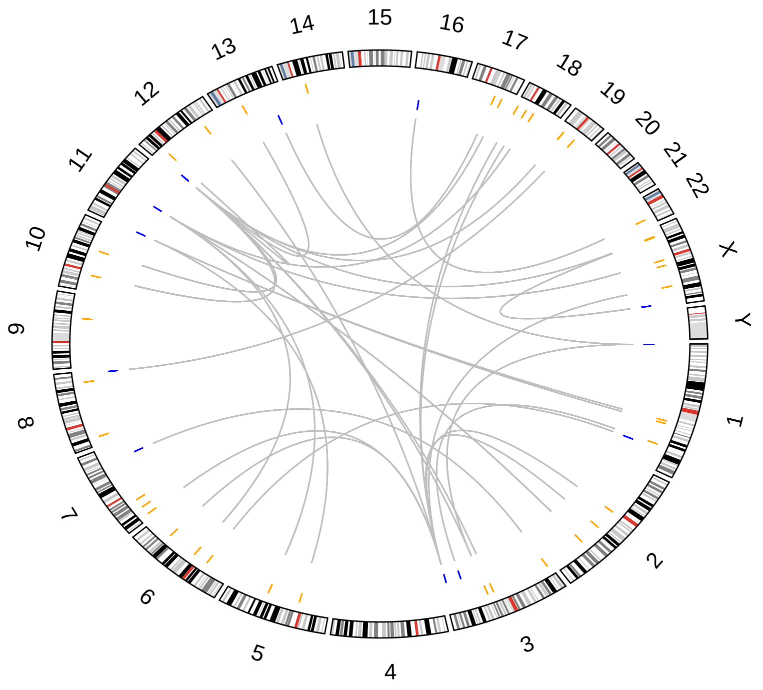

Figure 8: Circos plot for the first 45 miRNA-mRNA pairs (sorted by adjusted p-value) that have: pval-corrected<0.05 and appear at least 1 times in the following databases: microCosm_v5_18, targetScan_v6.2_18. Blue: miRNAs, Orange: target mRNAs

miRNA	mRNA	cor	adj.pval	FC.miRNA	FC.mRNA	dat.sum
hsa-miR-194	GLI3	-0.72	4.02e-26	1.15	1.06	2
hsa-miR-141	ZCCHC24	-0.68	1.71e-22	1.99	-2.01	1
hsa-miR-944	SLC41A2	-0.68	2.40e-22	8.25	-2.31	2
hsa-miR-944	HNMT	-0.67	2.80e-21	8.25	-1.38	2
hsa-miR-192	MCC	-0.67	5.45e-21	1.30	1.01	1
hsa-miR-210	DARC	-0.66	1.08e-20	2.25	-4.51	1
hsa-miR-200c	ZCCHC24	-0.66	1.17e-20	1.75	-2.01	1
hsa-miR-944	LONRF3	-0.66	1.33e-20	8.25	-1.66	1
hsa-miR-192	KCNQ5	-0.64	1.53e-18	1.30	-2.52	1
hsa-miR-151-5p	FXYD1	-0.63	2.22e-18	1.89	-6.73	1
hsa-miR-200c	AP1S2	-0.62	8.54e-18	1.75	-1.03	2
hsa-miR-192	FHOD3	-0.62	9.08e-18	1.30	-1.29	1
hsa-miR-192	MSN	-0.62	9.36e-18	1.30	1.65	1
hsa-miR-17	SGCA	-0.62	1.09e-17	2.58	-3.67	1
hsa-miR-200c	ZEB1	-0.62	1.11e-17	1.75	-1.30	2
hsa-miR-141	ZEB1	-0.62	2.49e-17	1.99	-1.30	1
hsa-miR-192	OSBPL6	-0.62	2.76e-17	1.30	-1.31	1
hsa-miR-200c	LHFP	-0.62	3.02e-17	1.75	-1.01	1
hsa-miR-200c	MYLK	-0.62	3.26e-17	1.75	-2.03	1
hsa-miR-16	LMOD1	-0.62	3.74e-17	1.75	-5.10	1
hsa-miR-944	GATA6	-0.61	4.60e-17	8.25	-2.24	2
hsa-miR-194	BICD2	-0.60	1.95e-16	1.15	1.61	1
hsa-miR-944	ARHGAP18	-0.60	2.65e-16	8.25	-1.48	1
hsa-miR-141	CCDC80	-0.60	2.76e-16	1.99	-1.98	2
hsa-miR-194	DFNA5	-0.60	4.16e-16	1.15	1.39	1
hsa-miR-193b	MGAT3	-0.60	6.13e-16	-1.03	-1.03	1
hsa-miR-194	MAGEE1	-0.60	6.36e-16	1.15	-2.40	1
hsa-miR-452	REPS2	-0.59	8.80e-16	3.41	-2.62	1
hsa-miR-205	ENPP4	-0.59	1.04e-15	5.41	-2.42	1
hsa-miR-96	ITPR1	-0.59	1.06e-15	2.75	-2.56	2
hsa-miR-944	MGAT4A	-0.59	1.53e-15	8.25	-1.12	1
hsa-miR-944	ICA1	-0.59	1.63e-15	8.25	-1.15	1
hsa-miR-15b	LMOD1	-0.59	2.16e-15	2.40	-5.10	1
hsa-miR-200c	ABCC9	-0.59	2.54e-15	1.75	-2.08	2
hsa-miR-429	MYLK	-0.59	2.59e-15	2.05	-2.03	1
hsa-miR-16	CNN1	-0.58	3.59e-15	1.75	-5.60	1
hsa-miR-200c	KANK2	-0.58	4.45e-15	1.75	-2.16	1
hsa-miR-141	MAP3K3	-0.58	4.48e-15	1.99	-1.11	1
hsa-miR-210	PPAP2A	-0.58	5.52e-15	2.25	-1.28	1
hsa-miR-944	LPIN2	-0.58	6.20e-15	8.25	-1.29	1
hsa-miR-194	TUSC3	-0.58	7.14e-15	1.15	1.37	1
hsa-miR-429	CFL2	-0.58	7.51e-15	2.05	-1.95	1
hsa-miR-194	MTSS1	-0.58	7.81e-15	1.15	-1.27	1
hsa-miR-210	NPR1	-0.58	7.87e-15	2.25	-2.41	1
hsa-miR-192*	OSBPL6	-0.58	7.93e-15	1.32	-1.31	1

Table 9: Top 45 miRNA-mRNA pairs(sorted by adjusted p-value) that have: pval-corrected<0.05 and appear at least 1 times in the following databases: microCosm_v5_18, targetScan_v6.2_18.

6 Functional analysis

6.1 Network analysis

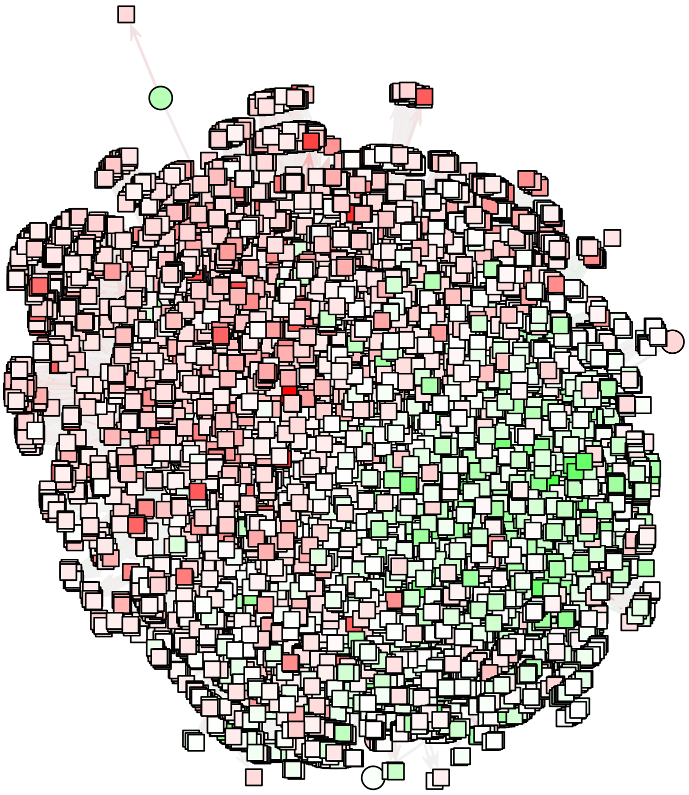

Figure 9: Network for all the miRNA-mRNA pairs that have: pval-corrected<0.05 and appear at least 1 times in the following databases: microCosm_v5_18, targetScan_v6.2_18. Circles represent the miRNAs, and squares the miRNA. Red fill means upregulated miRNAs/mRNAs, while green fill means downregulated mRNA/mRNAs in comparative CvH; lines indicate the miRNA-mRNA pairs, red line means positive score and green line means negative score.

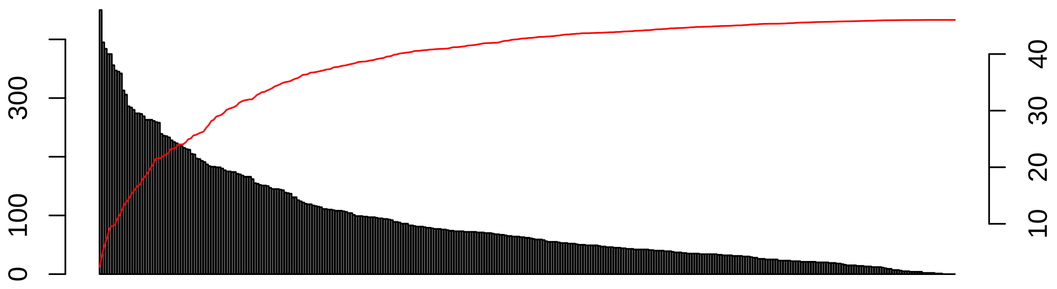

Figure 10: Barplot for miRNAs, pval-corrected<0.05 and Targets=microCosm_v5_18, targetScan_v6.2_18(minimum coincidences between databases:1). Red line (and right axis) represents the percentage of deregulated mRNAs that are targeted by the miRNAs.

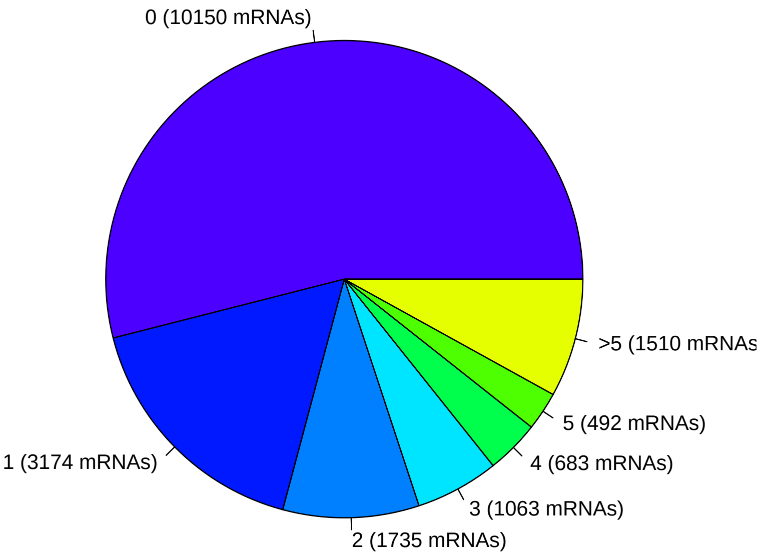

Figure 11: Pie chart representing the number of miRNAs targeting the mRNAs, pval-corrected<0.05 and Targets=microCosm_v5_18, targetScan_v6.2_18(minimum coincidences between databases:1).

miRNA	#targets	cum. %	targets (top 20)
**hsa-miR-27a**	450	2.39	ADCY6, PDK4, SELENBP1, RALGPS1, GCC2, DET1, NFATC2, SH3BGRL2, GPR133, SORBS1, PIP5K1B, EEPD1, PLEKHA6, REPS2, RAB11FIP2, PBXIP1, PTPRB, MAN2A2, NR2F2, SPATA13
**hsa-miR-29c**	395	4.49	EIF2S1, FBXO45, CSE1L, ODF2, REXO4, RCC2, TUBA1B, CDK2, CCNA2, TMEM201, MTHFD1L, YY1, YWHAE, COL11A1, VTA1, C9orf69, GGCT, ULBP2, MAP4K4, C1orf135
**hsa-miR-203**	384	6.23	ANXA6, ZEB1, RBPMS, SLC1A7, GNG2, MEF2C, GPRC5B, C16orf45, FKBP7, PPAP2B, PARD3B, RUNX1T1, FOXP1, DIXDC1, INSR, RGS5, ITGA9, PRKCB, FGL2, COL4A4
**hsa-miR-23a**	375	7.62	ESRRG, TSPAN12, UBL3, PLCXD3, SELENBP1, MYOCD, RAB11FIP2, FAM46C, MAGI1, DAPK1, LRRC31, KLHDC7A, SLC4A4, LRIG1, PTPRB, GPR64, ZC4H2, TOX3, REPS2, AFF3
**hsa-miR-429**	375	9.26	MYLK, CFL2, NFASC, DNAJB5, AP1S2, ABCC9, CLIC4, ZCCHC24, FHL1, LHFP, ZFPM2, TLN1, SYDE1, NPTX1, QKI, OSTM1, KANK2, PBX3, NCS1, FSTL1
**hsa-miR-200c**	356	9.62	ZCCHC24, AP1S2, ZEB1, LHFP, MYLK, ABCC9, KANK2, FHL1, TLN1, CFL2, ZNF423, RECK, RUNX1T1, FSTL1, ZEB2, TIMP2, GPRASP1, ZFPM2, NFASC, DLC1
**hsa-miR-200b**	347	9.76	CFL2, DNAJB5, MYLK, NFASC, AP1S2, SYDE1, LHFP, ZCCHC24, NCS1, QKI, ZFPM2, ABCC9, CLIC4, NPTX1, FHL1, FSTL1, CITED2, OSTM1, TLN1, KANK2
**hsa-miR-96**	345	10.89	ITPR1, FILIP1, LDB3, TNS1, DIXDC1, ZEB1, CACNA1C, POPDC2, CSRNP1, FYCO1, LMOD1, ITM2A, LRCH2, PPP1R12C, MAP3K3, ACVRL1, PDE7B, MYOCD, JAZF1, CELF2
**hsa-miR-27b**	342	11.62	GCC2, FNDC3A, NR5A2, DMXL2, DENND5B, LPIN2, NKTR, RAPGEF2, ACAA2, GFPT1, NLK, PEAK1, SLC30A7, TMED5, CDR2, RAB20, PLEKHH1, ZC3H12D, GCA, GOLM1
**hsa-miR-944**	313	12.6	SLC41A2, HNMT, LONRF3, GATA6, ARHGAP18, MGAT4A, ICA1, LPIN2, VPS13C, SLC12A2, NR3C2, HSD17B11, FOXP1, THRA, C2orf88, PTPRB, TMEM50B, C20orf112, C11orf54, SEPSECS

Table 10: Top 10 miRNA with more targets (each miRNA-mRNA pair has pval-corrected<0.05 and appears at least 1 times in the following databases: microCosm_v5_18, targetScan_v6.2_18). MiRNAs in red are upregulated in CvH, miRNAs in green are downregulated in CvH.

mRNA	#miRNAs	miRNAs (top 20)
**BNC2**	45	hsa-miR-141, hsa-miR-200c, hsa-miR-590-5p, hsa-miR-200c*, hsa-miR-96, hsa-miR-429, hsa-miR-191, hsa-let-7g*, hsa-miR-19a, hsa-miR-183, hsa-miR-592, hsa-miR-17, hsa-miR-3127-5p, hsa-miR-182, hsa-miR-130b, hsa-miR-19b, hsa-miR-196a, hsa-miR-200b, hsa-miR-425, hsa-miR-532-5p
**LPP**	42	hsa-miR-590-3p, hsa-miR-18a, hsa-miR-1976, hsa-miR-141, hsa-miR-96, hsa-miR-31, hsa-miR-19b, hsa-miR-92a, hsa-miR-203, hsa-miR-183, hsa-miR-425, hsa-miR-19a, hsa-miR-16, hsa-miR-424, hsa-miR-32, hsa-miR-942, hsa-miR-182, hsa-miR-142-3p, hsa-miR-330-5p, hsa-miR-200a
**NRP2**	40	hsa-miR-141, hsa-miR-429, hsa-miR-200c, hsa-miR-200b, hsa-miR-16, hsa-miR-200a, hsa-miR-532-5p, hsa-let-7g*, hsa-miR-30e, hsa-miR-19b, hsa-miR-425*, hsa-miR-19a, hsa-miR-577, hsa-miR-30b, hsa-miR-339-5p, hsa-miR-106a, hsa-miR-130b, hsa-miR-30d, hsa-miR-592, hsa-miR-17
**RUNX1T1**	40	hsa-miR-200c, hsa-miR-33a, hsa-miR-203, hsa-miR-455-3p, hsa-miR-16, hsa-miR-92a, hsa-miR-15b, hsa-miR-429, hsa-miR-130b, hsa-miR-19a, hsa-miR-200b, hsa-miR-19b, hsa-miR-584, hsa-miR-15a, hsa-miR-320b, hsa-miR-27a, hsa-miR-103a, hsa-miR-148b, hsa-let-7d, hsa-miR-23a
**FOXP2**	37	hsa-miR-192*, hsa-miR-29a*, hsa-miR-877, hsa-miR-660, hsa-miR-222, hsa-miR-19a, hsa-miR-7, hsa-miR-501-3p, hsa-miR-21, hsa-miR-196a, hsa-miR-15a, hsa-miR-34a, hsa-miR-92a, hsa-miR-577, hsa-miR-7-1*, hsa-miR-671-5p, hsa-miR-186, hsa-miR-503, hsa-miR-502-3p, hsa-miR-19b
**IGF1**	36	hsa-miR-19a, hsa-miR-18a, hsa-miR-942, hsa-miR-196b, hsa-miR-19b, hsa-miR-15b, hsa-miR-16, hsa-miR-222, hsa-miR-130b, hsa-miR-15a, hsa-miR-196a, hsa-miR-576-5p, hsa-miR-1976, hsa-miR-378, hsa-miR-503, hsa-miR-425, hsa-miR-192, hsa-miR-335*, hsa-miR-629, hsa-miR-590-3p
**TSHZ3**	35	hsa-miR-194, hsa-miR-141, hsa-miR-200c, hsa-miR-429, hsa-miR-200b, hsa-miR-577, hsa-miR-200a, hsa-miR-502-3p, hsa-let-7g*, hsa-miR-17*, hsa-miR-19b, hsa-miR-501-3p, hsa-miR-19a, hsa-miR-590-3p, hsa-miR-20a*, hsa-miR-18a, hsa-miR-106b, hsa-miR-106a, hsa-miR-335, hsa-miR-148a*
**KCNMA1**	34	hsa-miR-17, hsa-miR-20a, hsa-miR-20a*, hsa-miR-135b, hsa-miR-335*, hsa-miR-16-2*, hsa-miR-33a, hsa-miR-942, hsa-miR-93, hsa-miR-192*, hsa-miR-106b, hsa-miR-425, hsa-miR-532-5p, hsa-miR-671-5p, hsa-miR-106b*, hsa-miR-21*, hsa-miR-29a*, hsa-miR-339-3p, hsa-miR-34a, hsa-miR-584
**RORA**	34	hsa-miR-20a*, hsa-miR-18a, hsa-miR-92a, hsa-miR-17, hsa-miR-19a, hsa-miR-335*, hsa-miR-16, hsa-miR-20a, hsa-miR-19b, hsa-miR-106b, hsa-miR-107, hsa-miR-3613-5p, hsa-miR-141, hsa-miR-183, hsa-miR-652, hsa-miR-15a, hsa-miR-21*, hsa-miR-92b, hsa-miR-550a, hsa-miR-148b
**DLC1**	32	hsa-miR-141, hsa-miR-200c, hsa-miR-141*, hsa-let-7a*, hsa-miR-200b*, hsa-miR-200a*, hsa-miR-17, hsa-miR-429, hsa-miR-16, hsa-miR-24, hsa-miR-19b-1*, hsa-miR-130b, hsa-miR-151-5p, hsa-miR-106b, hsa-miR-20a, hsa-miR-200b, hsa-miR-19b, hsa-let-7b*, hsa-miR-19a, hsa-miR-93

Table 11: Top 10 mRNA with more miRNAs targeting them (each miRNA-mRNA pair has pval-corrected<0.05 and appears at least 1 times in the following databases: microCosm_v5_18, targetScan_v6.2_18). MRNAs in red are upregulated in CvH, mRNAs in green are downregulated in CvH.

6.2 GO analysis

GOBPID	Term	Count	Size	ExpCount	OddsRatio	fdr	Pvalue
GO:0048731	system development	1968	3416	1664.51	1.59	1.03e-28	9.45e-33
GO:0009653	anatomical structure morphogenesis	1280	2131	1038.37	1.71	2.51e-26	4.60e-30
GO:0007399	nervous system development	1108	1818	885.86	1.76	1.40e-25	3.86e-29
GO:0016043	cellular component organization	2562	4619	2250.70	1.49	1.83e-25	8.81e-29
GO:0023052	signaling	2652	4798	2337.92	1.48	1.83e-25	1.01e-28
GO:0044700	single organism signaling	2652	4798	2337.92	1.48	1.83e-25	1.01e-28
GO:0007154	cell communication	2687	4871	2373.49	1.47	3.14e-25	2.02e-28
GO:0065007	biological regulation	4653	8895	4334.26	1.45	2.20e-24	1.61e-27
GO:0071840	cellular component organization or biogenesis	2599	4715	2297.47	1.46	9.27e-24	7.64e-27
GO:0007165	signal transduction	2404	4333	2111.34	1.47	1.76e-23	1.61e-26

Table 12: Biological Process . Options used: mRNAs that are present in a mRNA-mRNA pair that has adjusted-pval cutoff <0.05; that also appears at least 1 times (databases: microCosm_v5_18, targetScan_v6.2_18); organism: human.

GOCCID	Term	Count	Size	ExpCount	OddsRatio	fdr	Pvalue
GO:0005737	cytoplasm	4858	9342	4302.38	1.73	3.70e-65	2.97e-68
GO:0044424	intracellular part	6105	12258	5645.33	1.79	5.00e-57	8.01e-60
GO:0005622	intracellular	6162	12396	5708.88	1.80	9.20e-57	2.21e-59
GO:0044444	cytoplasmic part	3608	6864	3161.16	1.56	6.63e-43	2.12e-45
GO:0043226	organelle	5573	11215	5164.98	1.57	1.16e-39	4.66e-42
GO:0043227	membrane-bounded organelle	5188	10399	4789.18	1.51	1.63e-35	7.82e-38
GO:0043229	intracellular organelle	5268	10617	4889.58	1.49	1.75e-32	9.80e-35
GO:0043231	intracellular membrane-bounded organelle	4765	9551	4398.64	1.44	9.52e-29	6.10e-31
GO:0005829	cytosol	1436	2545	1172.08	1.64	4.37e-28	3.15e-30
GO:0044464	cell part	6915	14496	6676.02	1.69	9.18e-27	7.35e-29

Table 13: Cellular Component . Options used: mRNAs that are present in a mRNA-mRNA pair that has adjusted-pval cutoff <0.05; that also appears at least 1 times (databases: microCosm_v5_18, targetScan_v6.2_18); organism: human.

GOMFID	Term	Count	Size	ExpCount	OddsRatio	fdr	Pvalue
GO:0005515	protein binding	4239	7904	3713.51	1.76	9.13e-63	2.92e-66
GO:0005488	binding	6009	12075	5673.15	1.76	7.76e-40	4.98e-43
GO:0008092	cytoskeletal protein binding	453	704	330.76	2.11	1.88e-18	1.81e-21
GO:0043168	anion binding	1378	2493	1171.28	1.49	5.65e-17	7.25e-20
GO:0019899	enzyme binding	697	1175	552.05	1.72	4.54e-16	7.27e-19
GO:0019904	protein domain specific binding	347	532	249.95	2.17	2.68e-15	5.16e-18
GO:0004672	protein kinase activity	365	565	265.45	2.12	3.08e-15	6.91e-18
GO:0016773	phosphotransferase activity, alcohol group as acceptor	426	679	319.01	1.96	9.17e-15	2.35e-17
GO:0016301	kinase activity	453	732	343.91	1.89	2.64e-14	7.62e-17
GO:0043167	ion binding	2966	5793	2721.70	1.32	4.87e-14	1.56e-16

Table 14: Molecular Function . Options used: mRNAs that are present in a mRNA-mRNA pair that has adjusted-pval cutoff <0.05; that also appears at least 1 times (databases: microCosm_v5_18, targetScan_v6.2_18); organism: human.

KEGGID	Term	Count	Size	ExpCount	OddsRatio	fdr	Pvalue
04510	Focal adhesion	142	192	92.71	3.15	3.62e-11	1.59e-13
05200	Pathways in cancer	209	314	151.61	2.23	1.63e-09	1.44e-11
04512	ECM-receptor interaction	65	81	39.11	4.43	1.89e-07	2.50e-09
04360	Axon guidance	87	125	60.36	2.50	5.03e-05	8.86e-07
04070	Phosphatidylinositol signaling system	58	78	37.66	3.15	8.53e-05	2.12e-06
05222	Small cell lung cancer	61	83	40.08	3.01	8.53e-05	2.25e-06
04916	Melanogenesis	71	100	48.28	2.67	9.28e-05	2.86e-06
04910	Insulin signaling pathway	88	133	64.22	2.13	5.56e-04	1.96e-05
04666	Fc gamma R-mediated phagocytosis	64	92	44.42	2.48	6.44e-04	2.55e-05
04270	Vascular smooth muscle contraction	74	111	53.60	2.17	1.38e-03	6.10e-05

Table 15: Kegg Pathways . Options used: mRNAs that are present in a mRNA-mRNA pair that has adjusted-pval cutoff <0.05; that also appears at least 1 times (databases: microCosm_v5_18, targetScan_v6.2_18); organism: human.
