# Supplementary material for: MiRComb: An R Package to Analyse miRNA-mRNA Interactions. Examples across Five Digestive Cancers
Source: PLoS One. 2016 Mar 11;11(3):e0151127. doi: 10.1371/journal.pone.0151127 (PMC4788200; doi:10.1371/journal.pone.0151127)
Supplement: S4 File — The report has been made by mkReport function. (PDF) [file pone.0151127.s007.pdf]

Default miRComb output

/home/mvila/Baixades/TCGA/stomach

May 13, 2015

1 Exploratory analysis of miRNA dataset

Number of miRNAs analysed	330
Number of samples	443

Table 1: Basic information of the miRNA dataset.

	group.n	CvH	center	sample	batch
1	NT: 37	Min. :0.0000	BR :138	TCGA-3M-AB46-01: 1	Batch 220: 58
2	TP:406	1st Qu.:1.0000	VQ : 66	TCGA-3M-AB47-01: 1	Batch 427: 58
3		Median :1.0000	CG : 43	TCGA-B7-5816-01: 1	Batch 269: 52
4		Mean :0.9165	HU : 41	TCGA-B7-5818-01: 1	Batch 57 : 31
5		3rd Qu.:1.0000	D7 : 40	TCGA-B7-A5TI-01: 1	Batch 95 : 29
6		Max. :1.0000	CD : 27	TCGA-B7-A5TJ-01: 1	Batch 242: 28
7			(Other): 88	(Other) :437	(Other) :187

Table 2: Summary of the phenotypical information of the miRNA dataset.

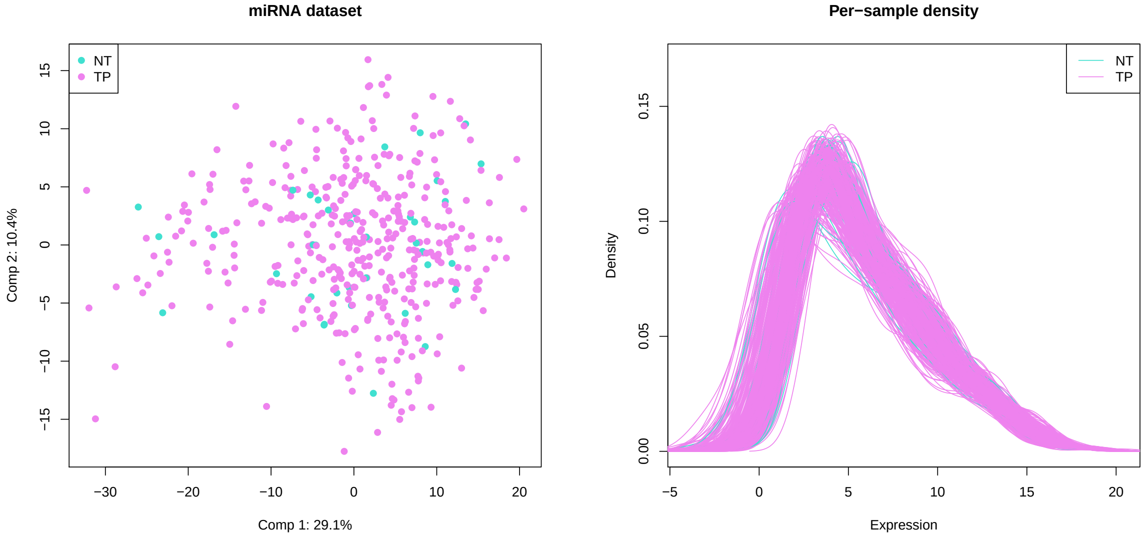

Figure 1: PCA and density plot for miRNAs.

2 Exploratory analysis of mRNA dataset

Number of mRNAs analysed	18565
Number of samples	443

Table 3: Basic information of the mRNA dataset.

	group.n	CvH	center	sample	batch
1	NT: 37	Min. :0.0000	BR :138	TCGA-3M-AB46-01: 1	Batch 220: 58
2	TP:406	1st Qu.:1.0000	VQ : 66	TCGA-3M-AB47-01: 1	Batch 427: 58
3		Median :1.0000	CG : 43	TCGA-B7-5816-01: 1	Batch 269: 52
4		Mean :0.9165	HU : 41	TCGA-B7-5818-01: 1	Batch 57 : 31
5		3rd Qu.:1.0000	D7 : 40	TCGA-B7-A5TI-01: 1	Batch 95 : 29
6		Max. :1.0000	CD : 27	TCGA-B7-A5TJ-01: 1	Batch 242: 28
7			(Other): 88	(Other) :437	(Other) :187

Table 4: Summary of the phenotypical information of the mRNA dataset.

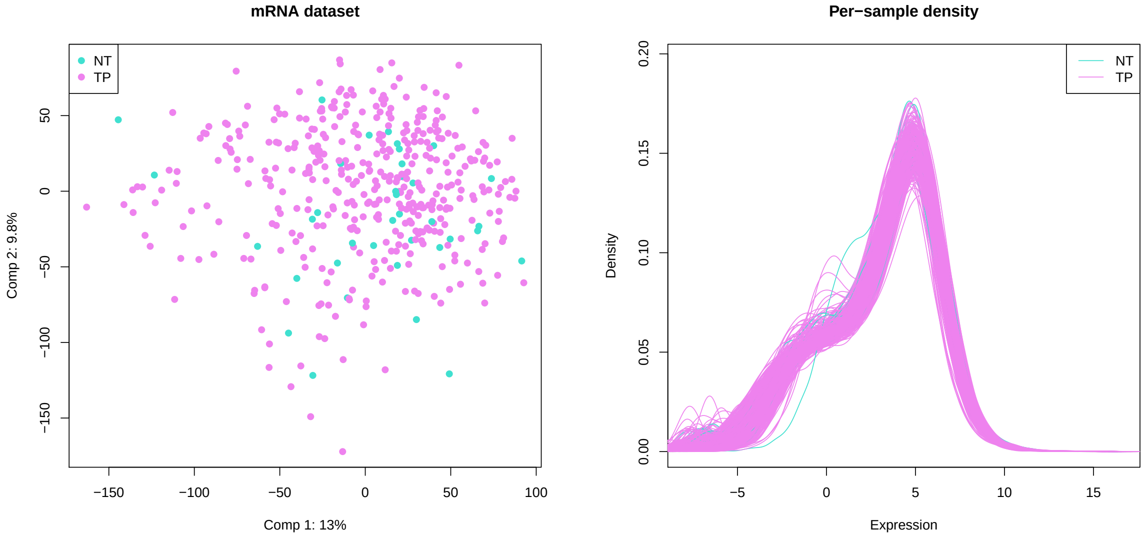

Figure 2: PCA and density plot for mRNAs.

3 Differentially expressed miRNAs

Analysis performed	Comparative used: CvH; method used: limma.
Number of differentially expressed miRNAs	330 (150 upregulated, 180 downregulated)
Number of samples	443
Criteria for selecting miRNAs	adj.pval < 1

Table 5: Basic statistics

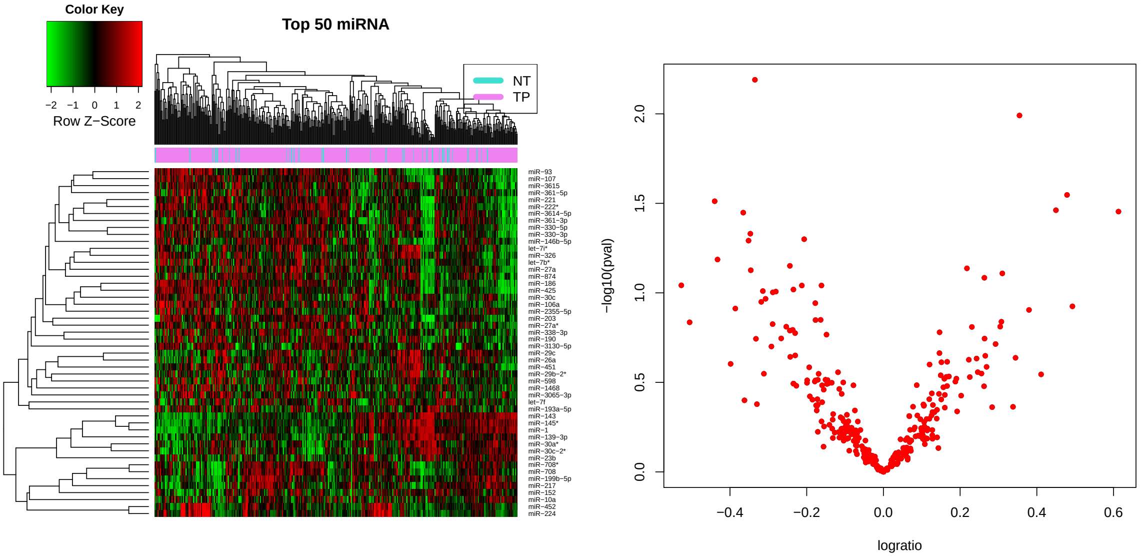

Figure 3: A) Heatmap vith the top 50 most significant miRNAs (sorted by adjusted p-value). B) Volcano plot showing the selected miRNAs.

4 Differentially expressed mRNAs

Analysis performed	Comparative used: CvH; method used: limma.
Number of differentially expressed mRNAs	18565 (10588 upregulated, 7977 downregulated)
Number of samples	443
Criteria for selecting mRNAs	adj.pval < 1

Table 6: Basic statistics

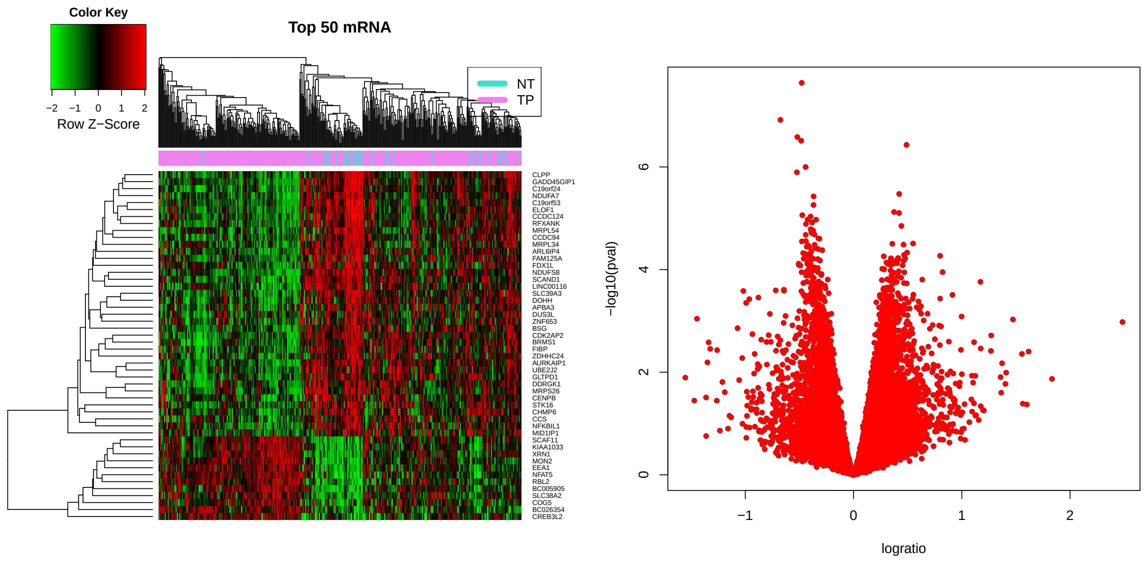

Figure 4: A) Heatmap vith the top 50 most significant mRNAs (sorted by adjusted p-value). B) Volcano plot showing the selected mRNAs.

5 Correlation & intersection with databases

Number of miRNAs	330
Number of mRNAs	18565
Total miRNA-mRNA combinations	6126450
Number of samples	443

Table 7: Number of miRNAs, mRNAs and samples used for correlation.

	Number	%
Total correlations	6126450	100
Total negative correlations	2828695	46.17
Total correlations p<0.05	1730533	28.25
Total correlations p<0.01	1367300	22.32
Total correlations adj.p<0.05	1390596	22.7
Total correlations adj.p<0.01	1108323	18.09

Table 8: Basic statistics for correlation results. Correlation hypothesis: two.sided.

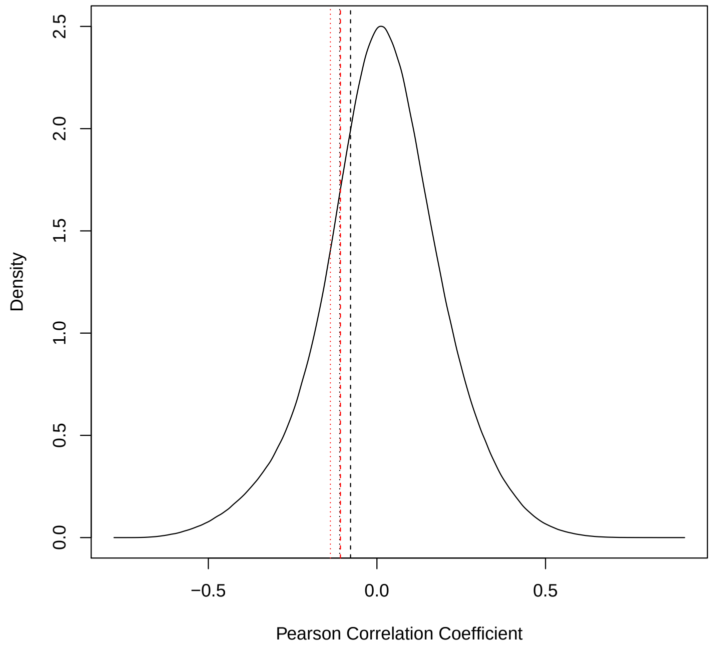

Figure 5: Density of a total of 6126450 miRNA-mRNA pairs. Dashed lines distinguish correlations whose p-value is lower than 0.05, dotted lines for 0.01. Black is for raw p-value and red for adjusted p-value.

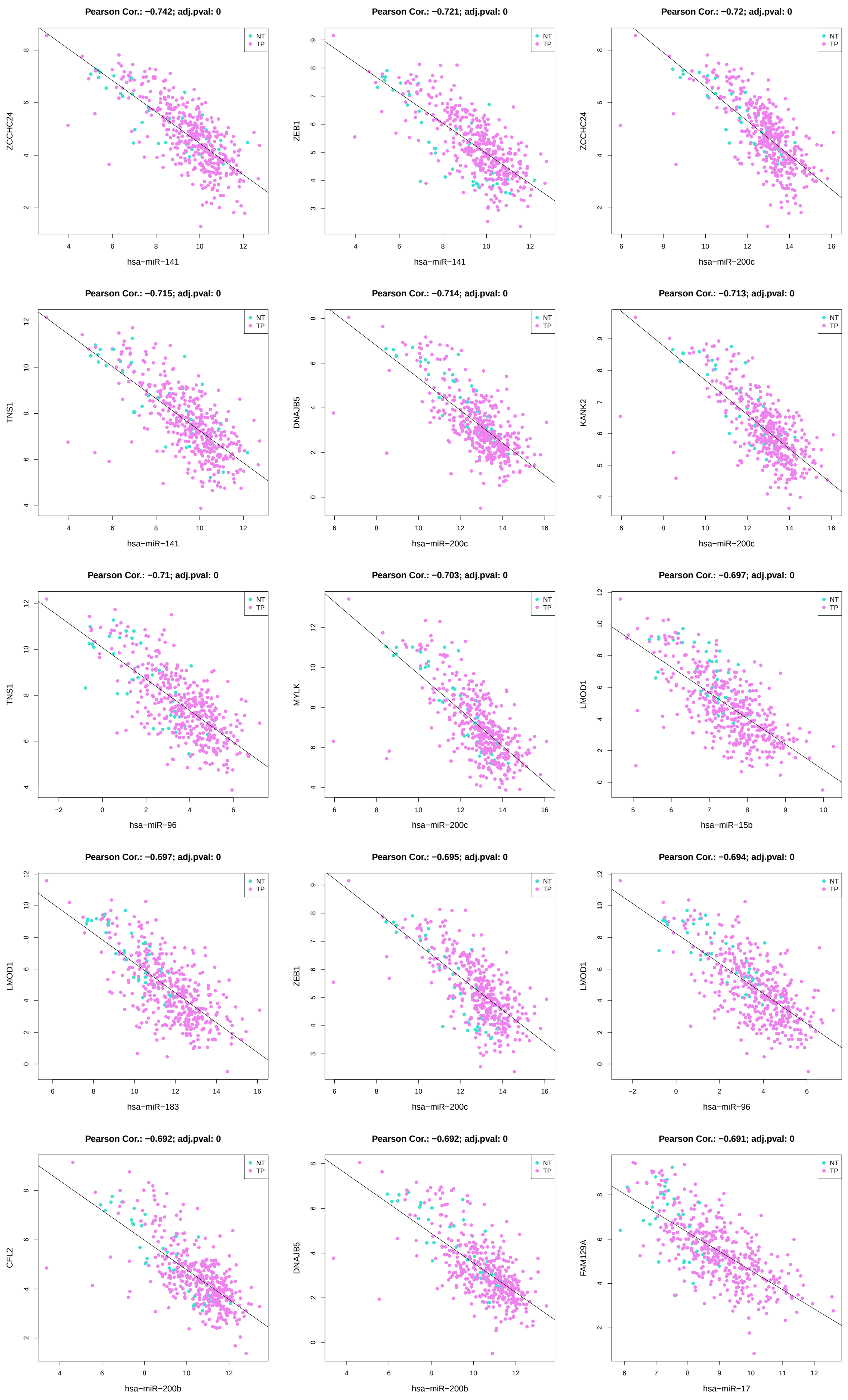

Figure 6: Plot of 15 top correlations, sorted by adjusted p-value. Databases used: microCosm_v5_18, targetScan_v6.2_18 (each miRNA-mRNA pair has to appear at least 1 times).

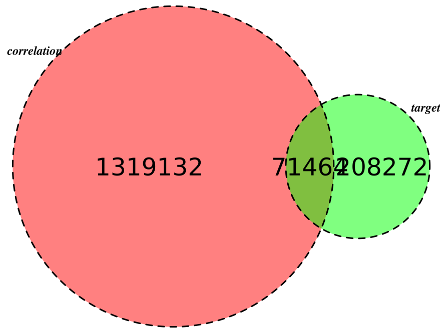

Figure 7: Venn Diagram. Left (red): number of miRNA-mRNA pairs with ajdusted p-value<0.05. Right (green): number of all the theoretical miRNA-mRNA pairs reported at least 1 times in the following databases: microCosm_v5_18, targetScan_v6.2_18. Intersection: miRNA-mRNA pairs that fulfil both conditions.

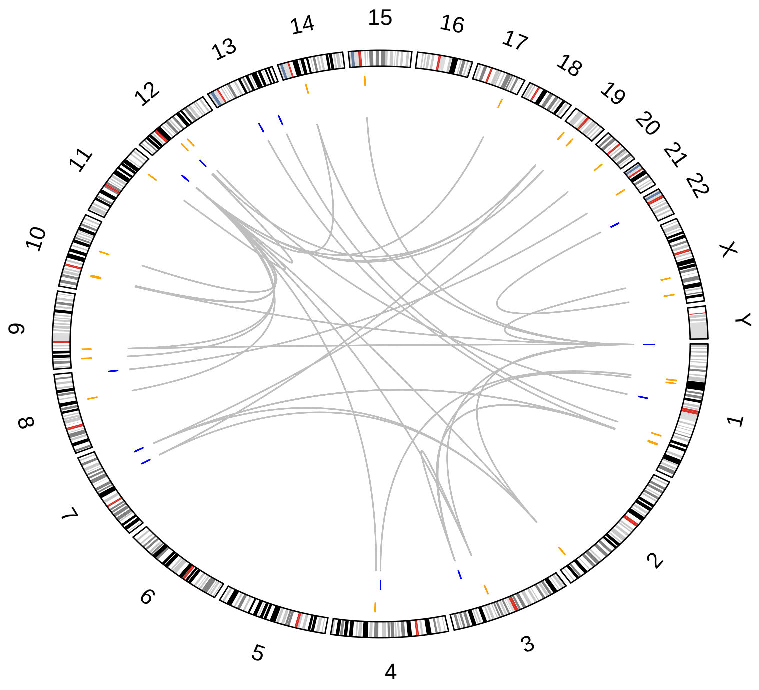

Figure 8: Circos plot for the first 45 miRNA-mRNA pairs (sorted by adjusted p-value) that have: pval-corrected<0.05 and appear at least 1 times in the following databases: microCosm_v5_18, targetScan_v6.2_18. Blue: miRNAs, Orange: target mRNAs

miRNA	mRNA	cor	adj.pval	FC.miRNA	FC.mRNA	dat.sum
hsa-miR-141	ZCCHC24	-0.74	1.01e-72	1.02	1.03	1
hsa-miR-141	ZEB1	-0.72	5.00e-67	1.02	1.24	1
hsa-miR-200c	ZCCHC24	-0.72	1.46e-66	-1.01	1.03	1
hsa-miR-141	TNS1	-0.72	2.39e-65	1.02	1.10	1
hsa-miR-200c	DNAJB5	-0.71	3.50e-65	-1.01	1.03	1
hsa-miR-200c	KANK2	-0.71	8.71e-65	-1.01	1.00	1
hsa-miR-96	TNS1	-0.71	4.03e-64	-1.05	1.10	1
hsa-miR-200c	MYLK	-0.70	1.49e-62	-1.01	1.15	1
hsa-miR-15b	LMOD1	-0.70	5.93e-61	-1.12	-1.08	1
hsa-miR-183	LMOD1	-0.70	5.93e-61	1.03	-1.08	1
hsa-miR-200c	ZEB1	-0.69	1.32e-60	-1.01	1.24	2
hsa-miR-96	LMOD1	-0.69	2.02e-60	-1.05	-1.08	1
hsa-miR-200b	CFL2	-0.69	5.90e-60	-1.01	1.15	1
hsa-miR-200b	DNAJB5	-0.69	7.89e-60	-1.01	1.03	1
hsa-miR-17	FAM129A	-0.69	1.38e-59	1.04	1.00	1
hsa-miR-15b	MYLK	-0.69	7.07e-59	-1.12	1.15	1
hsa-miR-15b*	NEXN	-0.69	1.45e-58	-1.10	1.02	1
hsa-miR-429	CFL2	-0.69	1.80e-58	-1.06	1.15	1
hsa-miR-141	DIXDC1	-0.69	1.94e-58	1.02	1.15	1
hsa-miR-130b*	FHL1	-0.68	1.45e-57	-1.04	1.14	1
hsa-miR-942	MSRB3	-0.68	2.22e-57	-1.13	1.25	1
hsa-miR-151-5p	JAM2	-0.68	6.62e-57	1.04	-1.00	1
hsa-miR-96	MYL9	-0.68	9.62e-57	-1.05	-1.08	1
hsa-miR-141	TSHZ3	-0.68	2.25e-56	1.02	1.13	2
hsa-miR-16	LMOD1	-0.68	2.42e-56	1.03	-1.08	1
hsa-miR-141	SVIL	-0.67	4.26e-56	1.02	1.23	1
hsa-miR-200a	TNS1	-0.67	4.96e-56	-1.01	1.10	1
hsa-miR-200c	CFL2	-0.67	5.07e-56	-1.01	1.15	1
hsa-miR-141	MAP3K3	-0.67	5.13e-56	1.02	1.06	1
hsa-miR-141	SYNPO2	-0.67	5.34e-56	1.02	1.16	1
hsa-miR-141	BNC2	-0.67	5.72e-56	1.02	1.31	1
hsa-miR-15a	LMOD1	-0.67	1.24e-55	1.06	-1.08	1
hsa-miR-106b	TNS1	-0.67	1.34e-55	-1.04	1.10	1
hsa-miR-429	MYLK	-0.67	1.57e-55	-1.06	1.15	1
hsa-miR-141	NECAB1	-0.67	1.87e-55	1.02	1.10	1
hsa-miR-200b*	GPRASP1	-0.67	3.08e-55	-1.11	1.13	1
hsa-miR-429	DNAJB5	-0.67	3.59e-55	-1.06	1.03	1
hsa-miR-200c	POU6F1	-0.67	1.09e-54	-1.01	1.07	1
hsa-miR-200a	SVIL	-0.67	1.14e-54	-1.01	1.23	1
hsa-miR-148b	CNN1	-0.67	1.73e-54	-1.05	-1.15	1
hsa-miR-200b	NDN	-0.67	2.37e-54	-1.01	-1.04	2
hsa-miR-15b	PRELP	-0.67	3.45e-54	-1.12	1.11	1
hsa-miR-576-5p	NEGR1	-0.67	3.77e-54	1.04	1.06	1
hsa-miR-106b	CNN1	-0.66	5.20e-54	-1.04	-1.15	2
hsa-miR-16	MYLK	-0.66	5.89e-54	1.03	1.15	1

Table 9: Top 45 miRNA-mRNA pairs(sorted by adjusted p-value) that have: pval-corrected<0.05 and appear at least 1 times in the following databases: microCosm_v5_18, targetScan_v6.2_18.

6 Functional analysis

6.1 Network analysis

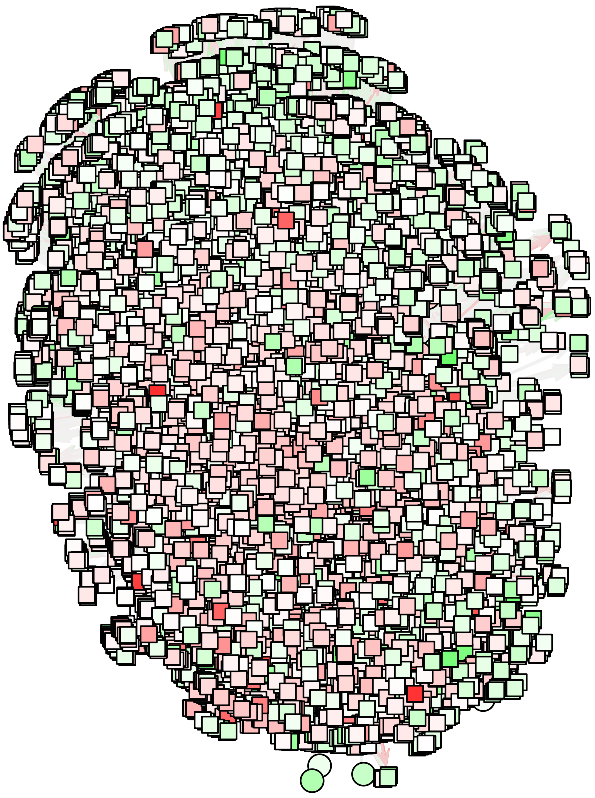

Figure 9: Network for all the miRNA-mRNA pairs that have: pval-corrected<0.05 and appear at least 1 times in the following databases: microCosm_v5_18, targetScan_v6.2_18. Circles represent the miRNAs, and squares the miRNA. Red fill means upregulated miRNAs/mRNAs, while green fill means downregulated mRNA/mRNAs in comparative CvH; lines indicate the miRNA-mRNA pairs, red line means positive score and green line means negative score.

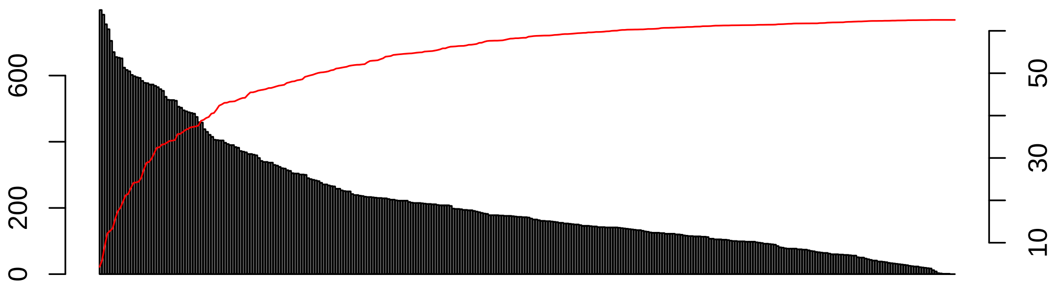

Figure 10: Barplot for miRNAs, pval-corrected<0.05 and Targets=microCosm_v5_18, targetScan_v6.2_18(minimum coincidences between databases:1). Red line (and right axis) represents the percentage of deregulated mRNAs that are targeted by the miRNAs.

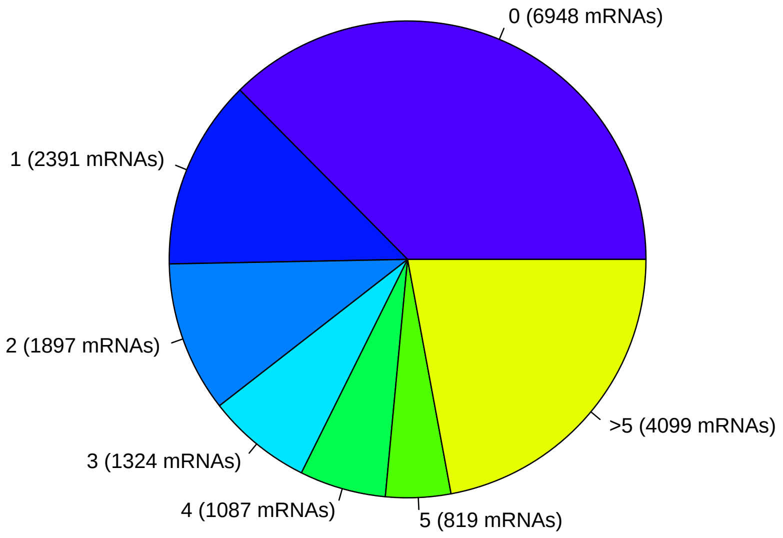

Figure 11: Pie chart representing the number of miRNAs targeting the mRNAs, pval-corrected<0.05 and Targets=microCosm_v5_18, targetScan_v6.2_18(minimum coincidences between databases:1).

miRNA	#targets	cum. %	targets (top 20)
**hsa-miR-29a**	798	4.3	FEM1B, LAMC1, STRN3, FAM168B, PRKAB2, CBX1, ITGB1, KPNA4, TMTC3, ARNT, RBFOX2, ENAH, SESTD1, WDR26, C5orf24, KDELC1, ELF2, DIP2C, BTBD7, JMY
**hsa-miR-29c**	784	5.93	COL11A1, FBXO45, PLEKHA8, KDELC1, CCNJ, CSE1L, LRRC58, MTHFD1L, CALU, KPNA4, USP31, CBX1, BMP1, TRIM37, XPO5, ABCE1, RIOK1, ZC3HAV1L, NKRF, SYNCRIP
**hsa-miR-195**	755	9.28	VMA21, TMEM33, ANLN, LRRC58, ZNF367, HNRNPA2B1, PEX13, SUZ12, NAA25, PSMD14, RAD23B, CLDN12, EPT1, CAPRIN1, FAM199X, SRPK1, GART, TMEM41B, ZNHIT3, KIAA1804
**hsa-miR-30b**	740	12.21	PITPNB, NRP2, SNTB2, MBNL1, HIPK3, FRMD6, DDR2, SSH1, HCFC2, OSTM1, LATS2, VSTM4, QKI, TEAD1, PTGFRN, AFF4, LPP, SGCB, EDNRA, PNMA1
**hsa-miR-29b**	705	12.78	FERMT2, RBFOX2, IQCJ-SCHIP1, LAMC1, IL17RD, PRICKLE2, SVIL, BACE1, GJC1, PRKG1, CRISPLD1, ELOVL4, AKT3, PTRF, WWTR1, TRO, DLG2, BVES, RUNX1T1, DAAM2
**hsa-miR-497**	671	13.36	ZNHIT3, VMA21, C17orf75, NAA25, LRRC58, G2E3, RAD23B, CLDN12, ZNF367, HNRNPA2B1, YWHAQ, PDIK1L, TMEM33, ANLN, SUZ12, IARS, CHORDC1, TBL1XR1, U2SURP, SLC5A3
**hsa-miR-429**	656	15.55	CFL2, MYLK, DNAJB5, KANK2, ZEB1, ZCCHC24, DNAJC18, ABCC9, POU6F1, CLIC4, GPRASP1, NDN, FHL1, DIXDC1, CACNB2, BNC2, AP1S2, GLI3, KCNMB1, NAP1L3
**hsa-miR-26a**	654	17.44	KPNA3, KIAA1737, ARPP19, UBR3, COPS2, KIAA1430, GSK3B, RANBP9, CDK8, FBXO28, TMEM106B, CBLL1, G3BP2, KPNA6, CORO1C, ZNF148, MTPN, STXBP4, IPO7, BTBD7
**hsa-miR-26b**	652	18.24	LPP, ZYG11B, TMEM43, ABCC9, MAP1B, FAM190B, FAM172A, ANKS1A, MAP4, STRN3, KIAA1737, B3GALNT2, NACC2, BBX, PLEKHM3, MMP16, CORO1C, OSTM1, ARPP19, CACNA1C
**hsa-miR-144**	624	19.69	LPP, LMO3, NAV3, PBX3, CDC42EP3, SYT11, ZFHX3, ZEB1, NRP2, CMYA5, MAGI2, DIXDC1, MBNL1, ENOX1, CRISPLD1, SLIT2, NRK, MEF2A, ABI2, NAP1L3

Table 10: Top 10 miRNA with more targets (each miRNA-mRNA pair has pval-corrected<0.05 and appears at least 1 times in the following databases: microCosm_v5_18, targetScan_v6.2_18). MiRNAs in red are upregulated in CvH, miRNAs in green are downregulated in CvH.

mRNA	#miRNAs	miRNAs (top 20)
**FOXP2**	74	hsa-miR-15b, hsa-miR-625, hsa-miR-141, hsa-miR-16, hsa-miR-130b*, hsa-miR-590-3p, hsa-miR-7-1*, hsa-miR-576-5p, hsa-miR-15a, hsa-miR-222, hsa-miR-502-3p, hsa-miR-96, hsa-miR-200a, hsa-miR-590-5p, hsa-let-7d, hsa-miR-671-5p, hsa-miR-7, hsa-miR-34a, hsa-miR-197, hsa-miR-182
**TNRC6B**	67	hsa-miR-130b, hsa-miR-26b, hsa-miR-144, hsa-miR-32, hsa-miR-590-3p, hsa-miR-106b, hsa-miR-16, hsa-miR-7, hsa-miR-3613-5p, hsa-miR-141, hsa-miR-424, hsa-miR-183, hsa-miR-29b, hsa-miR-29a, hsa-miR-590-5p, hsa-miR-455-3p, hsa-miR-3614-5p, hsa-miR-15b, hsa-miR-330-3p, hsa-miR-18a
**QKI**	65	hsa-miR-429, hsa-miR-141, hsa-miR-200a, hsa-miR-200b, hsa-miR-200c, hsa-miR-96, hsa-miR-532-3p, hsa-miR-194, hsa-miR-130b, hsa-miR-19b, hsa-miR-33a, hsa-miR-92a, hsa-miR-106b, hsa-miR-345, hsa-miR-17, hsa-miR-93*, hsa-miR-500b, hsa-miR-148a, hsa-miR-577, hsa-miR-19a
**RORA**	65	hsa-miR-141, hsa-miR-106b, hsa-miR-18a, hsa-miR-17, hsa-miR-200a, hsa-miR-15b, hsa-miR-33a, hsa-miR-3613-5p, hsa-miR-19b, hsa-miR-92a, hsa-miR-576-5p, hsa-miR-590-3p, hsa-miR-502-3p, hsa-miR-671-5p, hsa-miR-652, hsa-miR-15a, hsa-miR-183, hsa-miR-19a, hsa-miR-93, hsa-miR-501-3p
**BNC2**	60	hsa-miR-141, hsa-miR-200c, hsa-miR-130b, hsa-miR-106b, hsa-miR-429, hsa-miR-96, hsa-miR-200a, hsa-miR-592, hsa-miR-200b, hsa-miR-7, hsa-miR-17, hsa-miR-19b, hsa-miR-183, hsa-miR-200c*, hsa-miR-191, hsa-miR-577, hsa-miR-590-5p, hsa-miR-7-1*, hsa-miR-182, hsa-miR-19a
**AFF4**	57	hsa-miR-30b, hsa-miR-374b, hsa-miR-106b, hsa-miR-92a, hsa-miR-29a, hsa-miR-429, hsa-miR-32, hsa-miR-197, hsa-miR-590-3p, hsa-miR-455-5p, hsa-miR-502-3p, hsa-miR-15b, hsa-miR-200c, hsa-miR-17, hsa-miR-25, hsa-miR-505, hsa-miR-374a, hsa-miR-660, hsa-miR-15a, hsa-miR-331-5p
**LPP**	57	hsa-miR-590-3p, hsa-miR-16, hsa-miR-141, hsa-miR-96, hsa-miR-942, hsa-miR-32, hsa-miR-183, hsa-miR-7, hsa-miR-1266, hsa-miR-15b, hsa-miR-182, hsa-miR-424, hsa-miR-1976, hsa-miR-576-5p, hsa-miR-19b, hsa-miR-18a, hsa-miR-425, hsa-miR-330-5p, hsa-miR-577, hsa-miR-200a
**RUNX1T1**	57	hsa-miR-15b, hsa-miR-130b, hsa-miR-16, hsa-miR-200c, hsa-miR-33a, hsa-miR-429, hsa-miR-148b, hsa-miR-455-3p, hsa-miR-32, hsa-miR-200b, hsa-miR-19b, hsa-miR-15a, hsa-miR-92a, hsa-miR-19a, hsa-let-7d, hsa-miR-192, hsa-miR-25, hsa-miR-107, hsa-miR-29b, hsa-miR-186
**CPEB4**	55	hsa-miR-92a, hsa-miR-942, hsa-miR-19b, hsa-miR-130b, hsa-miR-197, hsa-miR-19a, hsa-miR-500b, hsa-miR-27a, hsa-miR-362-5p, hsa-miR-25, hsa-miR-96, hsa-miR-550a, hsa-miR-590-3p, hsa-miR-3647-3p, hsa-miR-128, hsa-let-7d, hsa-miR-26b, hsa-miR-141, hsa-miR-374b, hsa-miR-192
**NFIA**	55	hsa-miR-130b, hsa-miR-590-5p, hsa-miR-141, hsa-miR-32, hsa-miR-942, hsa-miR-93*, hsa-miR-191, hsa-miR-7, hsa-miR-33a, hsa-miR-454, hsa-miR-25, hsa-miR-200c, hsa-miR-429, hsa-miR-19a, hsa-miR-19b, hsa-miR-301a, hsa-miR-330-3p, hsa-miR-577, hsa-miR-200a, hsa-miR-92a

Table 11: Top 10 mRNA with more miRNAs targeting them (each miRNA-mRNA pair has pval-corrected<0.05 and appears at least 1 times in the following databases: microCosm_v5_18, targetScan_v6.2_18). MRNAs in red are upregulated in CvH, mRNAs in green are downregulated in CvH.

6.2 GO analysis

GOBPID	Term	Count	Size	ExpCount	OddsRatio	fdr	Pvalue
GO:0009987	cellular process	8586	12932	8354.90	1.91	6.41e-31	5.53e-35
GO:0006464	cellular protein modification process	1931	2583	1668.78	1.78	1.51e-30	3.90e-34
GO:0036211	protein modification process	1931	2583	1668.78	1.78	1.51e-30	3.90e-34
GO:0016043	cellular component organization	3304	4619	2984.17	1.58	5.28e-30	1.82e-33
GO:0071840	cellular component organization or biogenesis	3365	4715	3046.19	1.57	1.57e-29	6.78e-33
GO:0043412	macromolecule modification	1994	2685	1734.68	1.74	4.11e-29	2.13e-32
GO:0044267	cellular protein metabolic process	2456	3370	2177.24	1.64	3.37e-28	2.03e-31
GO:0006793	phosphorus metabolic process	2061	2809	1814.79	1.65	3.34e-25	2.51e-28
GO:0050794	regulation of cellular process	5447	7941	5130.40	1.46	3.34e-25	2.59e-28
GO:0044237	cellular metabolic process	6031	8855	5720.90	1.47	4.77e-25	4.12e-28

Table 12: Biological Process . Options used: mRNAs that are present in a mRNA-mRNA pair that has adjusted-pval cutoff <0.05; that also appears at least 1 times (databases: microCosm_v5_18, targetScan_v6.2_18); organism: human.

GOCCID	Term	Count	Size	ExpCount	OddsRatio	fdr	Pvalue
GO:0044424	intracellular part	8246	12258	7540.33	2.46	2.62e-137	1.95e-140
GO:0005622	intracellular	8322	12396	7625.22	2.47	7.87e-137	1.17e-139
GO:0005737	cytoplasm	6465	9342	5746.60	2.10	7.01e-115	1.56e-117
GO:0043226	organelle	7566	11215	6898.74	2.13	1.04e-109	3.10e-112
GO:0043227	membrane-bounded organelle	7078	10399	6396.79	2.08	6.74e-108	2.50e-110
GO:0043229	intracellular organelle	7191	10617	6530.89	2.05	9.60e-103	4.28e-105
GO:0043231	intracellular membrane-bounded organelle	6546	9551	5875.16	2.00	5.33e-101	2.78e-103
GO:0044444	cytoplasmic part	4827	6864	4222.29	1.91	2.79e-84	1.66e-86
GO:0044446	intracellular organelle part	4392	6274	3859.36	1.80	1.26e-67	8.44e-70
GO:0044422	organelle part	4502	6449	3967.01	1.80	1.78e-67	1.33e-69

Table 13: Cellular Component . Options used: mRNAs that are present in a mRNA-mRNA pair that has adjusted-pval cutoff <0.05; that also appears at least 1 times (databases: microCosm_v5_18, targetScan_v6.2_18); organism: human.

GOMFID	Term	Count	Size	ExpCount	OddsRatio	fdr	Pvalue
GO:0005515	protein binding	5593	7904	4974.68	2.03	2.25e-93	6.53e-97
GO:0005488	binding	8058	12075	7599.85	2.18	5.07e-77	2.95e-80
GO:0043167	ion binding	3986	5793	3646.04	1.51	1.80e-29	1.57e-32
GO:0043168	anion binding	1821	2493	1569.06	1.74	1.28e-28	1.49e-31
GO:1901265	nucleoside phosphate binding	1660	2255	1419.27	1.78	1.52e-28	2.21e-31
GO:0000166	nucleotide binding	1659	2254	1418.64	1.78	1.53e-28	2.67e-31
GO:0017076	purine nucleotide binding	1348	1819	1144.86	1.80	1.77e-24	3.61e-27
GO:0097367	carbohydrate derivative binding	1548	2115	1331.15	1.73	1.87e-24	4.34e-27
GO:0003824	catalytic activity	3571	5197	3270.93	1.47	2.28e-24	5.96e-27
GO:0032555	purine ribonucleotide binding	1334	1800	1132.90	1.80	2.36e-24	6.84e-27

Table 14: Molecular Function . Options used: mRNAs that are present in a mRNA-mRNA pair that has adjusted-pval cutoff <0.05; that also appears at least 1 times (databases: microCosm_v5_18, targetScan_v6.2_18); organism: human.

KEGGID	Term	Count	Size	ExpCount	OddsRatio	fdr	Pvalue
05200	Pathways in cancer	253	314	201.06	2.43	6.42e-09	3.88e-11
04510	Focal adhesion	163	192	122.94	3.26	6.42e-09	5.65e-11
04722	Neurotrophin signaling pathway	105	125	80.04	3.01	3.89e-05	5.14e-07
04810	Regulation of actin cytoskeleton	161	203	129.99	2.21	5.85e-05	1.14e-06
04141	Protein processing in endoplasmic reticulum	132	163	104.37	2.44	5.85e-05	1.29e-06
05100	Bacterial invasion of epithelial cells	61	69	44.18	4.34	1.58e-04	4.18e-06
04666	Fc gamma R-mediated phagocytosis	78	92	58.91	3.18	2.45e-04	7.57e-06
05222	Small cell lung cancer	71	83	53.15	3.37	2.51e-04	1.01e-05
05215	Prostate cancer	74	87	55.71	3.24	2.51e-04	1.02e-05
04360	Axon guidance	102	125	80.04	2.53	2.51e-04	1.11e-05

Table 15: Kegg Pathways . Options used: mRNAs that are present in a mRNA-mRNA pair that has adjusted-pval cutoff <0.05; that also appears at least 1 times (databases: microCosm_v5_18, targetScan_v6.2_18); organism: human.
