# Supplementary material for: MiRComb: An R Package to Analyse miRNA-mRNA Interactions. Examples across Five Digestive Cancers
Source: PLoS One. 2016 Mar 11;11(3):e0151127. doi: 10.1371/journal.pone.0151127 (PMC4788200; doi:10.1371/journal.pone.0151127)
Supplement: S5 File — The report has been made by mkReport function. (PDF) [file pone.0151127.s008.pdf]

Default miRComb output

/home/mvila/Baixades/TCGA/liver

June 4, 2015

1 Exploratory analysis of miRNA dataset

Number of miRNAs analysed	343
Number of samples	407

Table 1: Basic information of the miRNA dataset.

	group.n	CvH	center	sample	batch
1	NT: 50	Min. :0.0000	DD :172	TCGA-2Y-A9GS-01: 1	Batch 425: 83
2	TP:357	1st Qu.:1.0000	G3 : 33	TCGA-2Y-A9GT-01: 1	Batch 100: 62
3		Median :1.0000	CC : 32	TCGA-2Y-A9GU-01: 1	Batch 203: 33
4		Mean :0.8771	BC : 31	TCGA-2Y-A9GV-01: 1	Batch 399: 32
5		3rd Qu.:1.0000	2Y : 20	TCGA-2Y-A9GW-01: 1	Batch 231: 24
6		Max. :1.0000	ED : 15	TCGA-2Y-A9GX-01: 1	Batch 384: 21
7			(Other):104	(Other) :401	(Other) :152

Table 2: Summary of the phenotypical information of the miRNA dataset.

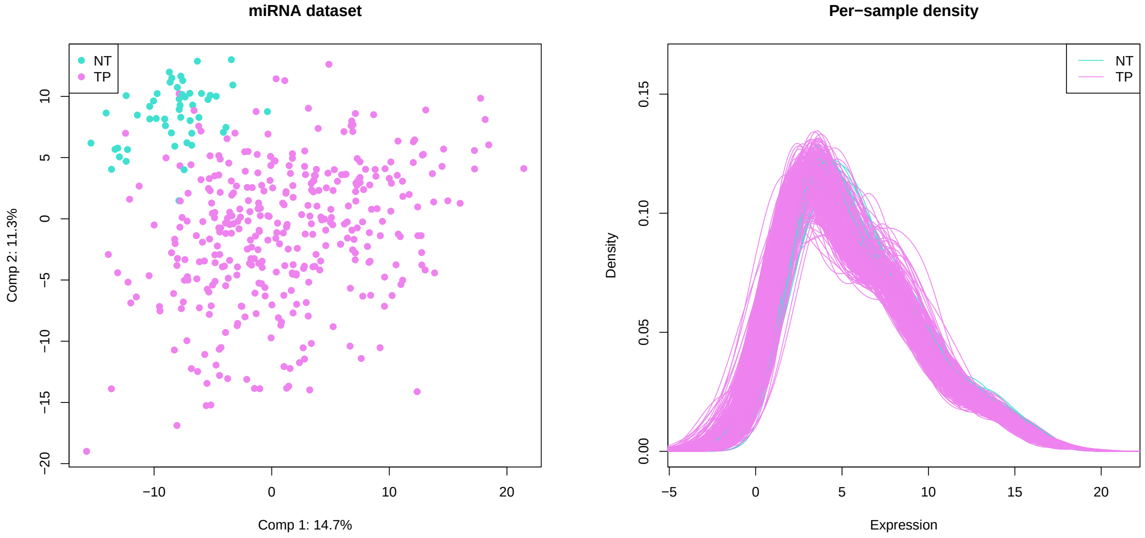

Figure 1: PCA and density plot for miRNAs.

2 Exploratory analysis of mRNA dataset

Number of mRNAs analysed	14428
Number of samples	407

Table 3: Basic information of the mRNA dataset.

	group.n	CvH	center	sample	batch
1	NT: 50	Min. :0.0000	DD :172	TCGA-2Y-A9GS-01: 1	Batch 425: 83
2	TP:357	1st Qu.:1.0000	G3 : 33	TCGA-2Y-A9GT-01: 1	Batch 100: 62
3		Median :1.0000	CC : 32	TCGA-2Y-A9GU-01: 1	Batch 203: 33
4		Mean :0.8771	BC : 31	TCGA-2Y-A9GV-01: 1	Batch 399: 32
5		3rd Qu.:1.0000	2Y : 20	TCGA-2Y-A9GW-01: 1	Batch 231: 24
6		Max. :1.0000	ED : 15	TCGA-2Y-A9GX-01: 1	Batch 384: 21
7			(Other):104	(Other) :401	(Other) :152

Table 4: Summary of the phenotypical information of the mRNA dataset.

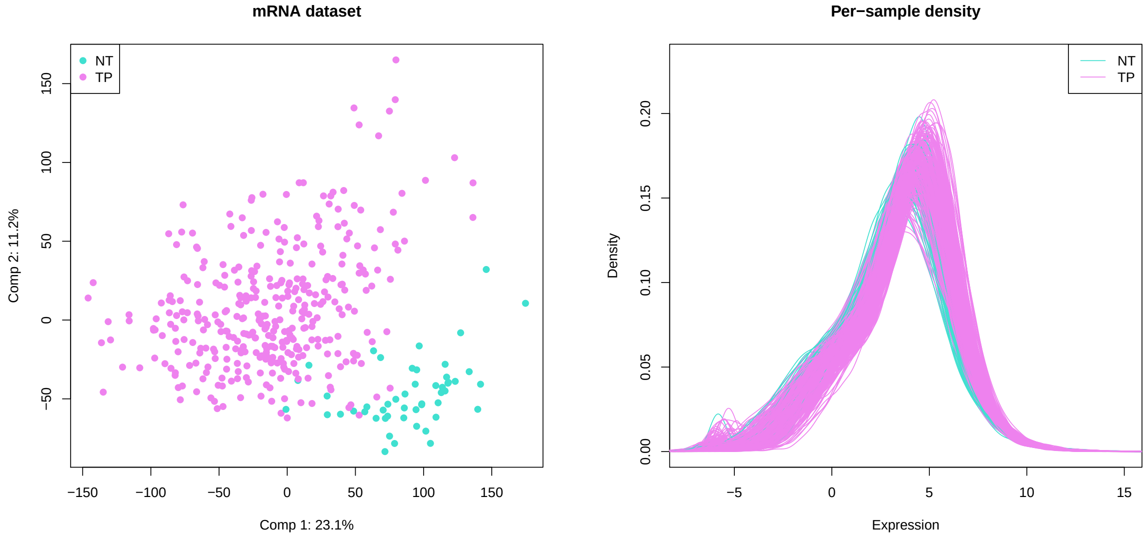

Figure 2: PCA and density plot for mRNAs.

3 Differentially expressed miRNAs

Analysis performed	Comparative used: CvH; method used: limma.
Number of differentially expressed miRNAs	343 (111 upregulated, 232 downregulated)
Number of samples	407
Criteria for selecting miRNAs	adj.pval < 1

Table 5: Basic statistics

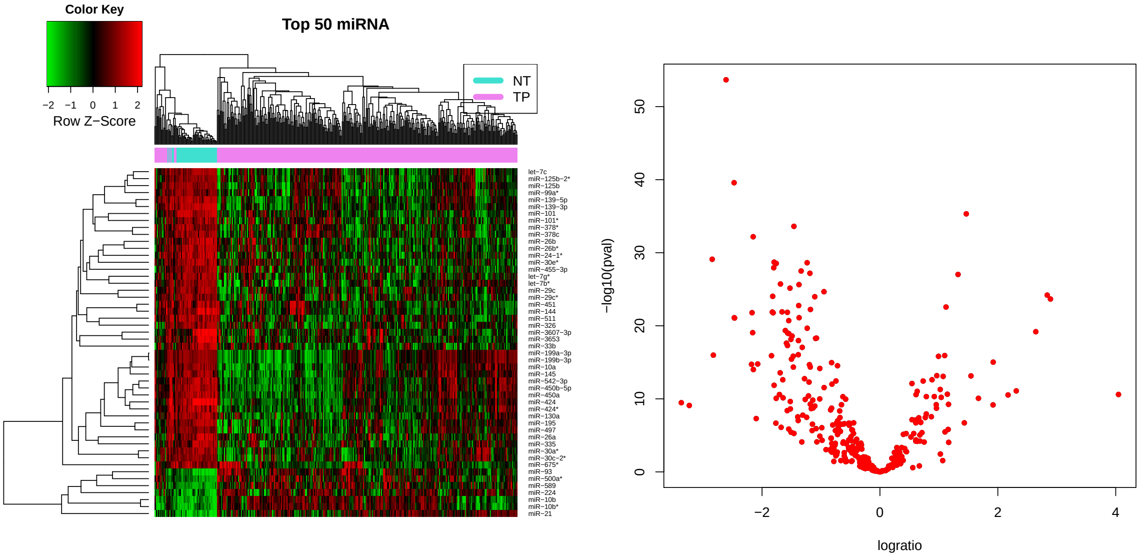

Figure 3: A) Heatmap vith the top 50 most significant miRNAs (sorted by adjusted p-value). B) Volcano plot showing the selected miRNAs.

4 Differentially expressed mRNAs

Analysis performed	Comparative used: CvH; method used: limma.
Number of differentially expressed mRNAs	14428 (11134 upregulated, 3294 downregulated)
Number of samples	407
Criteria for selecting mRNAs	adj.pval < 1

Table 6: Basic statistics

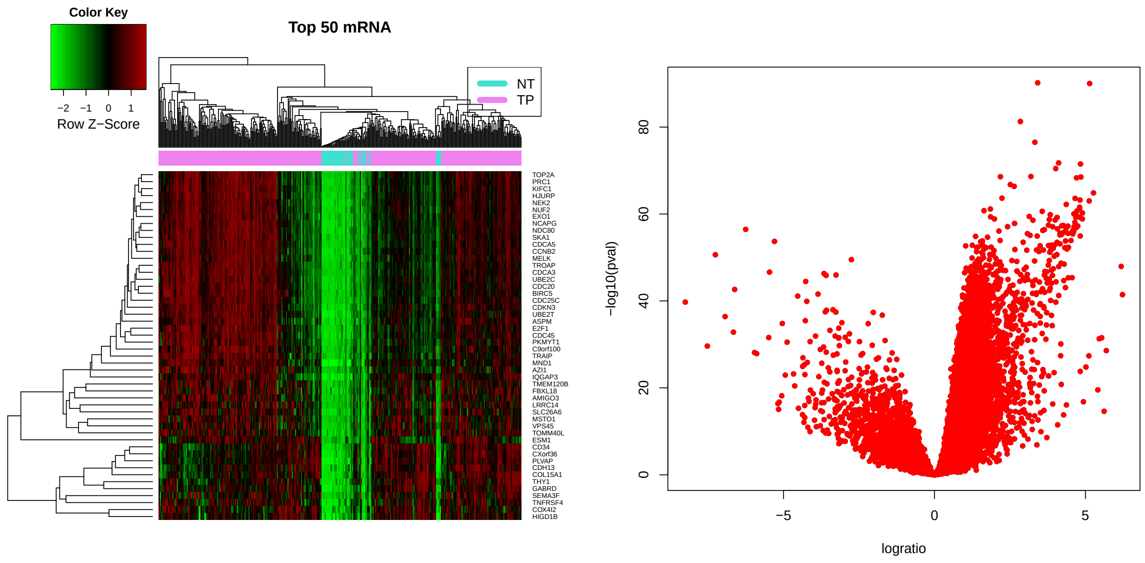

Figure 4: A) Heatmap vith the top 50 most significant mRNAs (sorted by adjusted p-value). B) Volcano plot showing the selected mRNAs.

5 Correlation & intersection with databases

Number of miRNAs	343
Number of mRNAs	14428
Total miRNA-mRNA combinations	4948804
Number of samples	407

Table 7: Number of miRNAs, mRNAs and samples used for correlation.

	Number	%
Total correlations	4948804	100
Total negative correlations	2424205	48.99
Total correlations p<0.05	1460936	29.52
Total correlations p<0.01	1130070	22.84
Total correlations adj.p<0.05	1156839	23.38
Total correlations adj.p<0.01	889513	17.97

Table 8: Basic statistics for correlation results. Correlation hypothesis: two.sided.

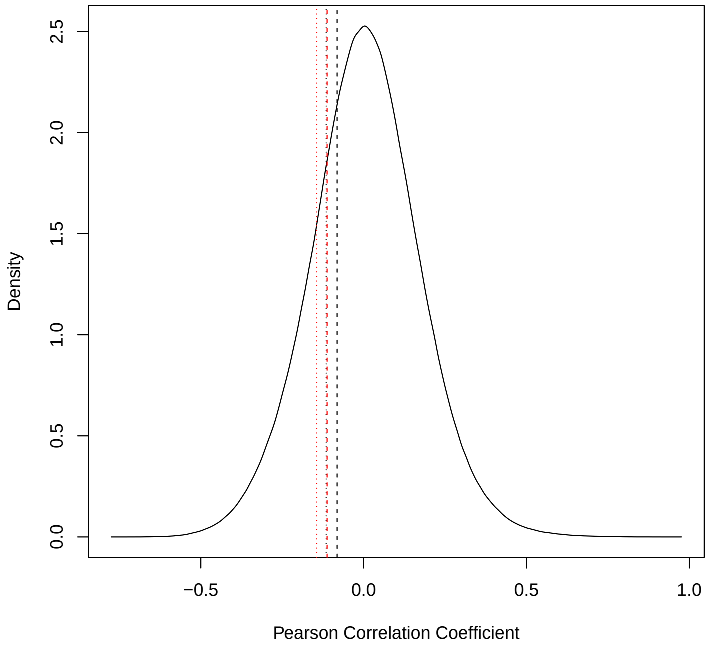

Figure 5: Density of a total of 4948804 miRNA-mRNA pairs. Dashed lines distinguish correlations whose p-value is lower than 0.05, dotted lines for 0.01. Black is for raw p-value and red for adjusted p-value.

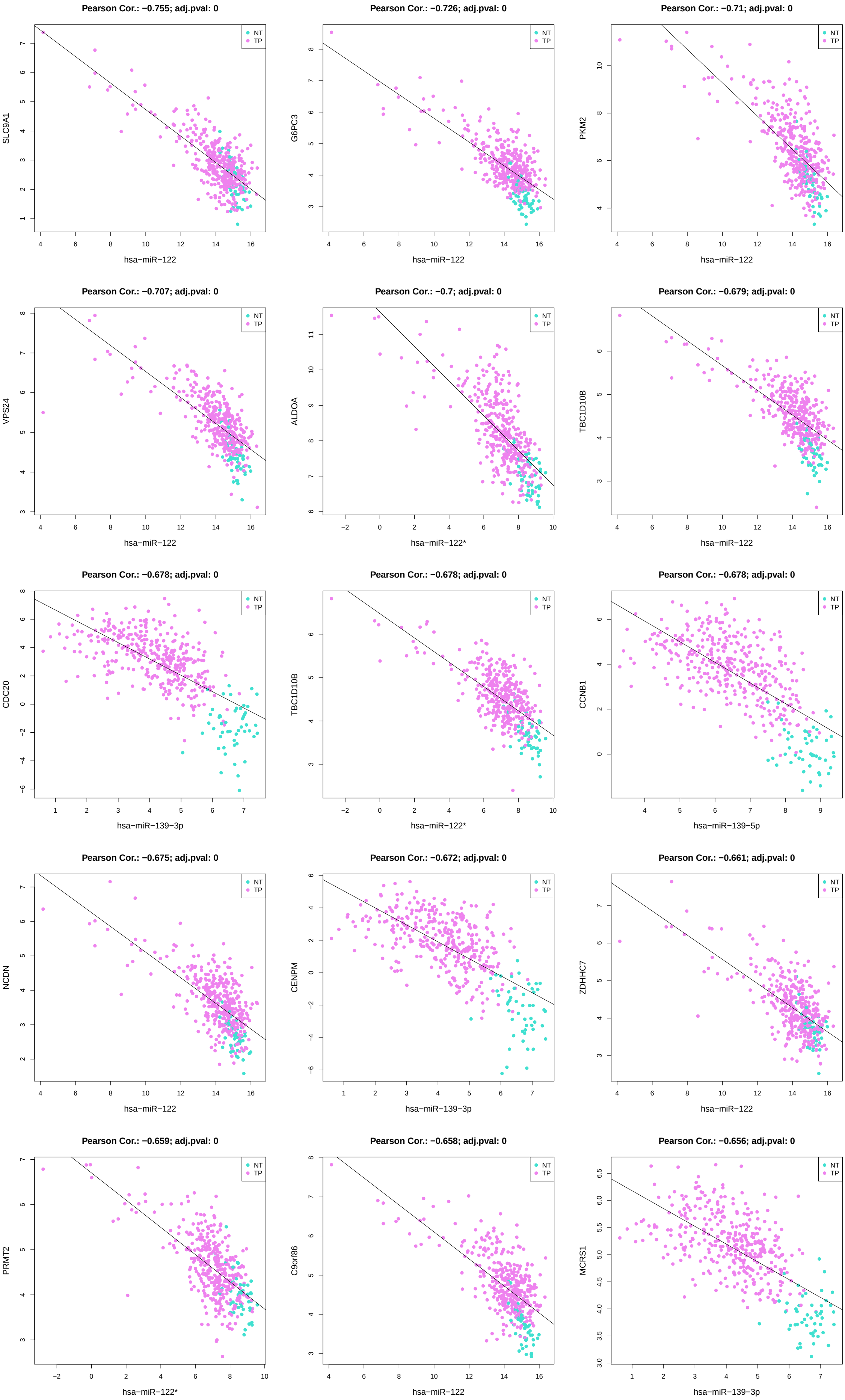

Figure 6: Plot of 15 top correlations, sorted by adjusted p-value. Databases used: microCosm_v5_18, targetScan_v6.2_18 (each miRNA-mRNA pair has to appear at least 1 times).

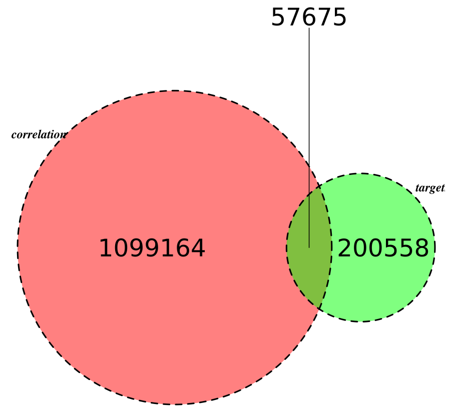

Figure 7: Venn Diagram. Left (red): number of miRNA-mRNA pairs with ajdusted p-value<0.05. Right (green): number of all the theoretical miRNA-mRNA pairs reported at least 1 times in the following databases: microCosm_v5_18, targetScan_v6.2_18. Intersection: miRNA-mRNA pairs that fulfil both conditions.

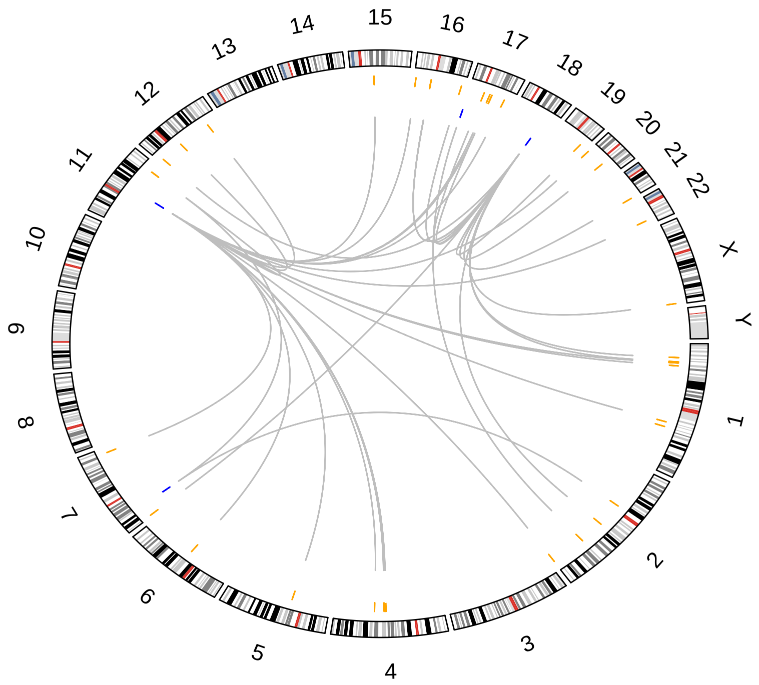

Figure 8: Circos plot for the first 45 miRNA-mRNA pairs (sorted by adjusted p-value) that have: pval-corrected<0.05 and appear at least 1 times in the following databases: microCosm_v5_18, targetScan_v6.2_18. Blue: miRNAs, Orange: target mRNAs

miRNA	mRNA	cor	adj.pval	FC.miRNA	FC.mRNA	dat.sum
hsa-miR-122	SLC9A1	-0.75	5.07e-70	-2.12	1.59	1
hsa-miR-122	G6PC3	-0.73	3.65e-62	-2.12	2.12	2
hsa-miR-122	PKM2	-0.71	4.30e-58	-2.12	2.94	1
hsa-miR-122	VPS24	-0.71	2.03e-57	-2.12	1.84	1
hsa-miR-122*	ALDOA	-0.70	5.94e-56	-3.23	2.52	1
hsa-miR-122	TBC1D10B	-0.68	2.71e-51	-2.12	1.92	2
hsa-miR-139-3p	CDC20	-0.68	3.66e-51	-5.56	29.84	1
hsa-miR-122*	TBC1D10B	-0.68	4.09e-51	-3.23	1.92	1
hsa-miR-139-5p	CCNB1	-0.68	4.09e-51	-4.44	12.46	1
hsa-miR-122	NCDN	-0.67	1.52e-50	-2.12	2.05	1
hsa-miR-139-3p	CENPM	-0.67	5.07e-50	-5.56	20.25	1
hsa-miR-122	ZDHHC7	-0.66	5.75e-48	-2.12	1.52	1
hsa-miR-122*	PRMT2	-0.66	1.44e-47	-3.23	1.55	1
hsa-miR-122	C9orf86	-0.66	1.90e-47	-2.12	2.19	2
hsa-miR-139-3p	MCRS1	-0.66	6.67e-47	-5.56	2.54	1
hsa-miR-122	GYS1	-0.65	1.76e-46	-2.12	1.86	1
hsa-miR-122	CHST12	-0.65	4.10e-46	-2.12	1.64	1
hsa-miR-139-5p	CDCA8	-0.65	1.13e-45	-4.44	12.19	1
hsa-miR-122	GIT1	-0.65	2.87e-45	-2.12	2.75	1
hsa-miR-139-3p	RBCK1	-0.64	5.54e-45	-5.56	2.42	1
hsa-miR-139-3p	CDC6	-0.64	2.52e-44	-5.56	14.70	1
hsa-miR-122	DULLARD	-0.64	6.06e-44	-2.12	1.52	1
hsa-miR-139-3p	DTYMK	-0.64	1.41e-43	-5.56	3.30	1
hsa-miR-139-5p	H2AFZ	-0.63	6.18e-43	-4.44	2.93	1
hsa-miR-122	ALDOA	-0.63	9.34e-43	-2.12	2.52	2
hsa-miR-148a	TMSB10	-0.63	2.82e-42	-1.61	2.61	2
hsa-miR-139-5p	TTK	-0.63	3.11e-42	-4.44	28.38	1
hsa-miR-139-5p	BUB1B	-0.63	8.04e-42	-4.44	19.62	1
hsa-miR-139-5p	MAD2L1	-0.62	1.91e-41	-4.44	5.89	1
hsa-miR-148a	TRAPPC4	-0.62	4.16e-41	-1.61	1.86	1
hsa-miR-139-3p	PKMYT1	-0.62	1.31e-40	-5.56	15.09	1
hsa-miR-139-5p	KPNA2	-0.62	1.64e-40	-4.44	3.79	1
hsa-miR-101	C20orf20	-0.62	2.02e-40	-2.75	2.64	2
hsa-miR-139-5p	EZH2	-0.62	4.06e-40	-4.44	8.44	1
hsa-miR-122	PLEKHB2	-0.62	5.51e-40	-2.12	1.44	1
hsa-miR-122	ATN1	-0.61	1.52e-39	-2.12	1.92	1
hsa-miR-125b	UCK2	-0.61	2.59e-39	-2.59	3.36	1
hsa-miR-139-5p	CENPE	-0.61	2.98e-39	-4.44	13.96	1
hsa-miR-122*	NDRG3	-0.61	4.91e-39	-3.23	2.71	1
hsa-miR-139-5p	CCT3	-0.61	6.23e-39	-4.44	3.09	1
hsa-miR-139-3p	H2AFX	-0.61	6.39e-39	-5.56	3.40	1
hsa-miR-122	SLC10A3	-0.61	1.02e-38	-2.12	1.79	1
hsa-miR-139-3p	SLC25A39	-0.61	2.13e-38	-5.56	2.42	1
hsa-miR-139-5p	ANAPC7	-0.60	7.30e-38	-4.44	2.53	1
hsa-miR-22	HNRNPA3	-0.60	8.53e-38	-1.50	1.90	1

Table 9: Top 45 miRNA-mRNA pairs(sorted by adjusted p-value) that have: pval-corrected<0.05 and appear at least 1 times in the following databases: microCosm_v5_18, targetScan_v6.2_18.

6 Functional analysis

6.1 Network analysis

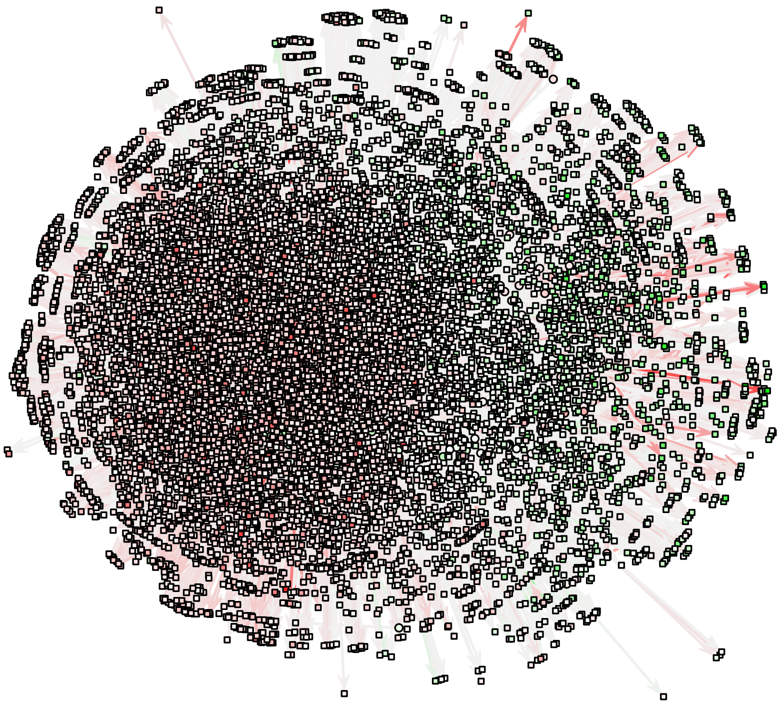

Figure 9: Network for all the miRNA-mRNA pairs that have: pval-corrected<0.05 and appear at least 1 times in the following databases: microCosm_v5_18, targetScan_v6.2_18. Circles represent the miRNAs, and squares the miRNA. Red fill means upregulated miRNAs/mRNAs, while green fill means downregulated mRNA/mRNAs in comparative CvH; lines indicate the miRNA-mRNA pairs, red line means positive score and green line means negative score.

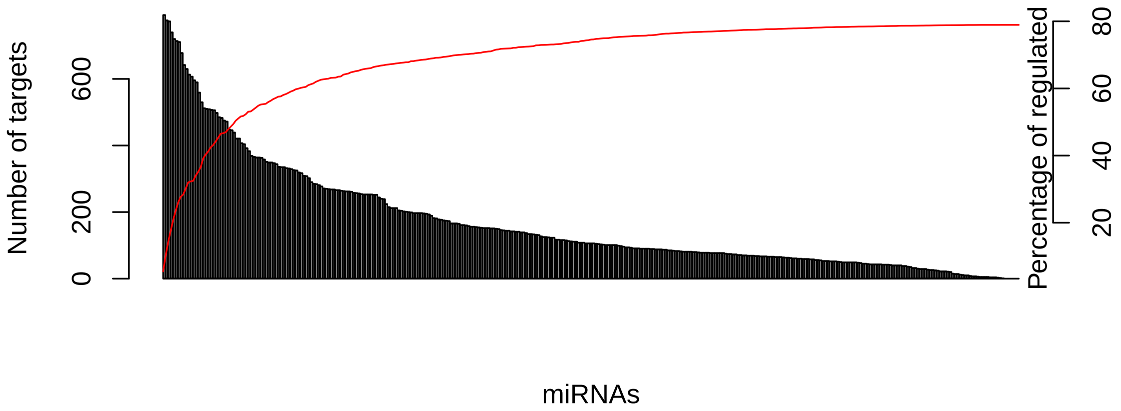

Figure 10: Barplot for miRNAs, pval-corrected<0.05 and Targets=microCosm_v5_18, targetScan_v6.2_18(minimum coincidences between databases:1). Red line (and right axis) represents the percentage of deregulated mRNAs that are targeted by the miRNAs.

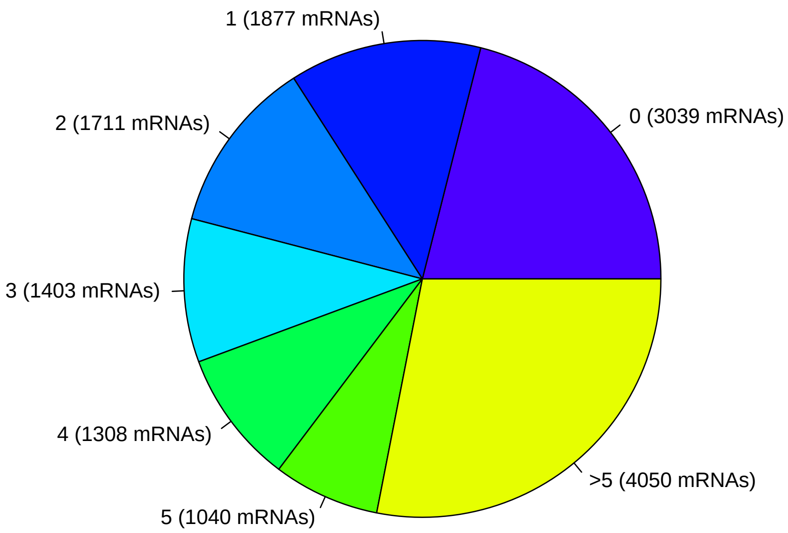

Figure 11: Pie chart representing the number of miRNAs targeting the mRNAs, pval-corrected<0.05 and Targets=microCosm_v5_18, targetScan_v6.2_18(minimum coincidences between databases:1).

miRNA	#targets	cum. %	targets (top 20)
**hsa-miR-27b**	792	5.49	LPCAT1, PSMD7, B3GNTL1, AAK1, KIAA0513, MRPS23, C1orf9, MSTO1, TPM3, HM13, RPN2, EFNA3, WDR45, KIF21B, LIMK1, GNS, DAP3, ACCN2, PSMA1, SLC7A11
**hsa-miR-29c**	776	10.24	FAM136A, MYBL2, DNMT3A, CCNA2, SUV420H2, CCDC99, ADSL, DDX49, C5orf13, XPO5, SOX12, TAF11, CENPE, TET1, PPP1CC, CSE1L, GSG2, LSM11, RTKN2, C20orf11
**hsa-let-7c**	773	14.24	AURKB, NME6, HMGA1, EIF2S2, CCT3, TIMM17B, EPS8L3, UFC1, ARID3A, UBE2T, SNRPD1, CENPA, ATP6V1F, EME1, SLC35B1, CCNF, C18orf21, RRM2, NME1, TTLL4
**hsa-miR-497**	740	17.54	APLN, SLC26A6, SRPK1, ACACA, GGA3, ZN-HIT3, SNRPC, NRBP1, PPIL1, EHMT2, PDCD11, HMGA1, XRCC6, ZBTB9, ZSCAN2, FAM189B, TOMM20, BTF3, SERPINI1, RECQL5
**hsa-miR-148a**	720	20.83	TMSB10, TRAPPC4, ZDHHC7, TUBB6, PCNXL2, VPS24, STX3, TAGLN2, CD151, CFL1, C9orf86, DNAJC18, MTHFD1L, ITGB4, FMNL3, SULF1, CTTNBP2NL, CHMP4B, JAG2, TMSL3
**hsa-miR-125b**	714	23.54	UCK2, HM13, SLC26A6, COPZ1, C9orf100, ELOVL1, SMG5, SRPRB, TMEM120B, TOMM40, PIGU, KIAA1522, SNRPB, BUB1B, E2F2, TMEM201, STX6, BAK1, MCRS1, UBE2I
**hsa-miR-101**	711	26.03	C20orf20, LASS5, C8orf76, C2orf29, DENR, TRIM11, PLXNA1, ANKRD52, DNMT3A, C8orf33, CCT4, IQ-GAP3, TUBA1C, NAP1L1, EZH2, ENAH, RIT1, UCK2, TCEB1, MELK
**hsa-miR-144**	678	27.71	SNRPE, PIGC, C1orf77, RAB11FIP4, SF3B4, DSTYK, RUSC1, ANKZF1, MEF2D, NOL12, GGPS1, ORMDL2, MRPS23, SNRPB, BCL9, LASS5, RASD2, DCTN2, TAF6, B3GALNT2
**hsa-miR-424**	642	28.4	APLN, AMIGO3, RECQL5, FAM189B, UBE2Q1, MXD3, SNRPC, BAT4, ZNHIT3, NSMCE2, TOMM20, MTX1, BCAP31, PUF60, E4F1, CDKN2A, DUS1L, PSKH1, NFKBIL1, TARBP1
**hsa-miR-30e**	630	30.12	C8orf76, YWHAZ, MTHFD1L, ZNF706, RASL12, NPC2, FKBP1A, MICAL1, CTHRC1, FBXO32, DTX2, P4HA2, C19orf50, NME6, FAP, PSMB5, STK39, STOML1, DGKZ, TMC7

Table 10: Top 10 miRNA with more targets (each miRNA-mRNA pair has pval-corrected<0.05 and appears at least 1 times in the following databases: microCosm_v5_18, targetScan_v6.2_18). MiRNAs in red are upregulated in CvH, miRNAs in green are downregulated in CvH.

mRNA	#miRNAs	miRNAs (top 20)
RORA	41	hsa-miR-18a, hsa-miR-324-3p, hsa-miR-17, hsa-miR-181b, hsa-miR-93, hsa-miR-92b, hsa-miR-20a, hsa-miR-183, hsa-miR-106b, hsa-miR-652, hsa-miR-421, hsa-miR-92a, hsa-miR-501-3p, hsa-miR-181a, hsa-miR-19a, hsa-miR-132, hsa-miR-155, hsa-miR-671-5p, hsa-miR-19b, hsa-miR-224
SAP30L	38	hsa-miR-511, hsa-miR-33b, hsa-miR-450a, hsa-miR-142-3p, hsa-miR-142-5p, hsa-miR-582-5p, hsa-miR-145, hsa-miR-450b-5p, hsa-miR-144, hsa-miR-203, hsa-miR-29c, hsa-miR-337-3p, hsa-miR-27a*, hsa-miR-136*, hsa-miR-29b, hsa-miR-26b, hsa-miR-378*, hsa-miR-493, hsa-let-7b, hsa-let-7c
ANKRD52	37	hsa-miR-101, hsa-miR-378c, hsa-let-7c, hsa-miR-29c, hsa-miR-22, hsa-miR-26b, hsa-miR-144, hsa-miR-378, hsa-miR-3607-3p, hsa-miR-33b, hsa-miR-148a, hsa-miR-130a, hsa-miR-328, hsa-miR-30e, hsa-miR-29a, hsa-miR-26a, hsa-miR-542-3p, hsa-miR-145, hsa-miR-885-3p, hsa-miR-326
CASK	36	hsa-miR-19b-1*, hsa-miR-885-5p, hsa-miR-92a, hsa-miR-101, hsa-miR-375, hsa-miR-497, hsa-miR-203, hsa-miR-483-3p, hsa-miR-424, hsa-miR-24-1*, hsa-miR-144, hsa-miR-758, hsa-miR-19b, hsa-miR-424*, hsa-miR-142-3p, hsa-miR-125b-2*, hsa-miR-411, hsa-miR-195, hsa-miR-142-5p, hsa-miR-194
NFIA	36	hsa-miR-21, hsa-miR-410, hsa-miR-301a, hsa-miR-889, hsa-miR-485-3p, hsa-miR-92b, hsa-miR-92a, hsa-miR-382, hsa-miR-200b, hsa-miR-19a, hsa-miR-155, hsa-miR-338-3p, hsa-miR-200a, hsa-miR-369-3p, hsa-miR-429, hsa-miR-19b, hsa-miR-421, hsa-miR-370, hsa-miR-501-3p, hsa-miR-134
MRPL43	34	hsa-let-7b*, hsa-miR-378*, hsa-miR-26a, hsa-miR-10a, hsa-miR-26b, hsa-miR-542-3p, hsa-miR-378, hsa-miR-22, hsa-miR-194*, hsa-miR-451, hsa-miR-342-5p, hsa-miR-22*, hsa-miR-142-3p, hsa-miR-885-5p, hsa-miR-214*, hsa-miR-99a, hsa-miR-152, hsa-miR-126, hsa-miR-215, hsa-miR-122
ATXN7L1	33	hsa-miR-375, hsa-miR-18a, hsa-miR-17, hsa-miR-92a, hsa-miR-20a*, hsa-miR-20a, hsa-miR-19b, hsa-miR-33b, hsa-miR-19a, hsa-miR-106a, hsa-miR-889, hsa-miR-148b, hsa-miR-495, hsa-miR-338-5p, hsa-miR-337-3p, hsa-miR-744*, hsa-miR-130a, hsa-miR-379, hsa-miR-338-3p, hsa-miR-199b-5p
HLF	33	hsa-miR-183, hsa-miR-181b, hsa-miR-331-5p, hsa-miR-181d, hsa-miR-18a, hsa-miR-181a, hsa-miR-199b-5p, hsa-miR-181c, hsa-miR-218, hsa-miR-200c, hsa-miR-338-5p, hsa-miR-652, hsa-let-7i, hsa-miR-141, hsa-miR-148b, hsa-miR-17, hsa-miR-425, hsa-miR-106b, hsa-miR-223, hsa-miR-200a
NFIB	33	hsa-miR-324-3p, hsa-miR-17, hsa-miR-192, hsa-miR-103a, hsa-miR-421, hsa-miR-20a, hsa-miR-92a, hsa-miR-539, hsa-miR-185, hsa-miR-19a, hsa-miR-410, hsa-miR-19b, hsa-miR-582-5p, hsa-miR-194, hsa-miR-382, hsa-miR-136*, hsa-miR-409-3p, hsa-miR-136, hsa-miR-301a, hsa-miR-495
HDAC8	32	hsa-miR-139-5p, hsa-miR-375, hsa-miR-483-3p, hsa-miR-199a-3p, hsa-miR-10a, hsa-let-7b*, hsa-miR-214*, hsa-miR-654-3p, hsa-miR-30e*, hsa-miR-125b, hsa-miR-20a*, hsa-miR-378*, hsa-miR-22, hsa-miR-30e, hsa-miR-326, hsa-miR-376c, hsa-let-7a*, hsa-miR-381, hsa-let-7e*, hsa-miR-21*

Table 11: Top 10 mRNA with more miRNAs targeting them (each miRNA-mRNA pair has pval-corrected<0.05 and appears at least 1 times in the following databases: microCosm_v5_18, targetScan_v6.2_18). MRNAs in red are upregulated in CvH, mRNAs in green are downregulated in CvH.

6.2 GO analysis

GOBPID	Term	Count	Size	ExpCount	OddsRatio	fdr	Pvalue
GO:0044237	cellular metabolic process	6098	8855	5531.80	1.99	7.67e-83	6.67e-87
GO:0008152	metabolic process	6625	9835	6144.01	1.87	2.48e-64	4.32e-68
GO:0071704	organic substance metabolic process	6270	9266	5788.55	1.82	1.85e-61	4.82e-65
GO:0044238	primary metabolic process	6092	8977	5608.01	1.81	4.81e-61	1.67e-64
GO:0044267	cellular protein metabolic process	2503	3370	2105.27	2.01	1.21e-57	5.24e-61
GO:0044260	cellular macromolecule metabolic process	4680	6758	4221.78	1.72	7.61e-53	3.97e-56
GO:0044248	cellular catabolic process	1485	1936	1209.44	2.17	2.26e-43	1.38e-46
GO:0044710	single-organism metabolic process	3289	4649	2904.27	1.71	3.76e-43	2.61e-46
GO:0071840	cellular component organization or biogenesis	3325	4715	2945.50	1.69	1.21e-41	9.44e-45
GO:0009056	catabolic process	1724	2296	1434.33	2.00	2.97e-41	2.59e-44

Table 12: Biological Process . Options used: mRNAs that are present in a mRNA-mRNA pair that has adjusted-pval cutoff <0.05; that also appears at least 1 times (databases: microCosm_v5_18, targetScan_v6.2_18); organism: human.

GOCCID	Term	Count	Size	ExpCount	OddsRatio	fdr	Pvalue
GO:0044424	intracellular part	8224	12258	7224.25	3.58	2.84e-273	2.17e-276
GO:0005622	intracellular	8285	12396	7305.58	3.57	7.34e-268	1.12e-270
GO:0005737	cytoplasm	6561	9342	5505.71	2.94	2.44e-244	5.58e-247
GO:0043227	membrane-bounded organelle	7129	10399	6128.65	2.89	5.62e-229	1.72e-231
GO:0043226	organelle	7565	11215	6609.56	2.93	9.15e-222	3.49e-224
GO:0043231	intracellular membrane-bounded organelle	6621	9551	5628.88	2.76	3.47e-217	1.59e-219
GO:0043229	intracellular organelle	7207	10617	6257.13	2.77	2.31e-209	1.23e-211
GO:0044444	cytoplasmic part	4974	6864	4045.30	2.69	4.43e-196	2.71e-198
GO:0044446	intracellular organelle part	4542	6274	3697.58	2.54	8.61e-168	5.91e-170
GO:0044422	organelle part	4627	6449	3800.72	2.45	1.29e-158	9.83e-161

Table 13: Cellular Component . Options used: mRNAs that are present in a mRNA-mRNA pair that has adjusted-pval cutoff <0.05; that also appears at least 1 times (databases: microCosm_v5_18, targetScan_v6.2_18); organism: human.

GOMFID	Term	Count	Size	ExpCount	OddsRatio	fdr	Pvalue
GO:0005515	protein binding	5539	7904	4816.90	2.25	1.84e-125	5.30e-129
GO:0005488	binding	7861	12075	7358.81	2.33	2.12e-91	1.22e-94
GO:0003824	catalytic activity	3646	5197	3167.18	1.84	1.20e-61	1.04e-64
GO:0044822	poly(A) RNA binding	886	1107	674.63	2.74	1.87e-42	2.16e-45
GO:0019899	enzyme binding	923	1175	716.08	2.50	3.92e-38	5.66e-41
GO:0003723	RNA binding	1121	1477	900.12	2.16	9.63e-35	1.67e-37
GO:1901363	heterocyclic compound binding	3758	5568	3393.28	1.56	2.60e-34	5.24e-37
GO:0097159	organic cyclic compound binding	3800	5641	3437.77	1.55	1.08e-33	2.50e-36
GO:0043168	anion binding	1785	2493	1519.30	1.76	1.61e-31	4.17e-34
GO:1901265	nucleoside phosphate binding	1618	2255	1374.25	1.76	8.25e-29	2.38e-31

Table 14: Molecular Function . Options used: mRNAs that are present in a mRNA-mRNA pair that has adjusted-pval cutoff <0.05; that also appears at least 1 times (databases: microCosm_v5_18, targetScan_v6.2_18); organism: human.

KEGGID	Term	Count	Size	ExpCount	OddsRatio	fdr	Pvalue
01100	Metabolic pathways	831	1116	729.73	1.70	6.50e-11	2.86e-13
05215	Prostate cancer	82	87	56.89	8.85	1.36e-08	1.20e-10
05200	Pathways in cancer	254	314	205.32	2.33	2.90e-08	3.90e-10
04120	Ubiquitin mediated proteolysis	114	128	83.70	4.42	2.90e-08	5.12e-10
04141	Protein processing in endoplasmic reticulum	140	163	106.58	3.31	8.94e-08	1.97e-09
05210	Colorectal cancer	59	61	39.89	15.85	9.98e-08	2.64e-09
05212	Pancreatic cancer	66	70	45.77	8.87	2.72e-07	8.38e-09
04110	Cell cycle	108	124	81.08	3.65	7.55e-07	2.81e-08
05220	Chronic myeloid leukemia	67	72	47.08	7.20	7.55e-07	2.99e-08
05211	Renal cell carcinoma	62	66	43.16	8.33	8.42e-07	3.71e-08

Table 15: Kegg Pathways . Options used: mRNAs that are present in a mRNA-mRNA pair that has adjusted-pval cutoff <0.05; that also appears at least 1 times (databases: microCosm_v5_18, targetScan_v6.2_18); organism: human.
